# The identification of the *Rosa S*-locus provides new insights into the breeding and wild origins of continuous-flowering roses

**DOI:** 10.1093/hr/uhac155

**Published:** 2022-10-01

**Authors:** Koji Kawamura, Yoshihiro Ueda, Shogo Matsumoto, Takanori Horibe, Shungo Otagaki, Li Wang, Guoliang Wang, Laurence Hibrand-Saint Oyant, Fabrice Foucher, Marcus Linde, Thomas Debener

**Affiliations:** Department of Environmental Engineering, Osaka Institute of Technology, Japan; Gifu International Academy of Horticulture, Japan; Gifu World Rose Garden, Japan; Graduate School of Bioagricultural Sciences, Nagoya University, Japan; Graduate School of Bioagricultural Sciences, Nagoya University, Japan; College of Bioscience and Biotechnology, Chubu University, Japan; Graduate School of Bioagricultural Sciences, Nagoya University, Japan; College of Life Sciences, Sichuan University, China; Jiangsu Provincial Department of Agriculture and Rural Affairs, China; Agricultural University of Nanjing, China; Univ Angers, INRAE, Institut Agro, IRHS, SFR QUASAV, F-49000 Angers, France; Univ Angers, INRAE, Institut Agro, IRHS, SFR QUASAV, F-49000 Angers, France; Leibniz Universität, Hannover, Germany; Leibniz Universität, Hannover, Germany

## Abstract

This study aims to: (i) identify the *Rosa S*-locus controlling self-incompatibility (SI); (ii) test the genetic linkage of the *S*-locus with other loci controlling important ornamental traits, such as the continuous-flowering (CF) characteristic; (iii) identify the *S*-alleles (*S_C_*) of old Chinese CF cultivars (e.g, Old Blush, Slater’s Crimson China) and examine the changes in the frequency of cultivars with *Sc* through the history of breeding; (iv) identify wild species carrying the *Sc*-alleles to infer wild origins of CF cultivars. We identified a new *S*-*RNase* (*S_C2_*) of *Rosa chinensis* in a contig from a genome database that has not been integrated into one of the seven chromosomes yet. Genetic mapping indicated that *S_C2_* is allelic to the previously-identified *S-RNase* (*S_C1_*) in chromosome 3. Pollination experiments with half-compatible pairs of roses confirmed that they are the pistil-determinant of SI. The segregation analysis of an *F_1_*-population indicated genetic linkage between the *S*-locus and the floral repressor gene *KSN.* The non-functional allele *ksn* is responsible for the CF characteristic. A total of five *S*-alleles (*S_C1–5_*) were identified from old CF cultivars. The frequency of cultivars with *S_C_* dramatically increased after the introgression of *ksn* from Chinese to European cultivars and remains high (80%) in modern cultivars, suggesting that *S*-genotyping is helpful for effective breeding. Wild individuals carrying *S_C_* were found in *Rosa multiflora* (*S_C1_*), *Rosa chinensis* var*. spontanea* (*S_C3_*), and *Rosa gigantea* (*S_C2_*, *S_C4_*), supporting the hypothesis of hybrid origins of CF cultivars and providing a new evidence for the involvement of *Rosa multiflora*.

## Introduction

The rose is one of the globally most popular ornamental plants, with a long history of breeding and cultivation. It is important not only economically, but also culturally. More than 30 000 rose cultivars have been developed [1] mainly by cross breeding. Patterns of inheritance are quite difficult to predict for most traits, as roses are predominantly outcrossing and highly heterozygous plants [2]. As a consequence, the success of cross breeding in the rose has largely depended on chance and the experience of breeders, requiring enormous efforts to make new cultivars with desirable traits [3]. Advances in scientific knowledge on rose genetics have been much awaited. Researchers have developed molecular markers to construct genetic linkage maps of the rose, clarifying the underlying genetic mechanisms controlling ornamental traits [4] and, genome sequencing finished in 2018 [5–7]. Key genetic factors controlling important ornamental traits, such as scent production [8, 9], continuous-flowering (CF) [10], and double-flower (DF) [5, 11] have been identified.

Although identification of the *S*-locus controlling self-incompatibility (SI) in roses is essential for improving breeding at the diploid level, it has not been fully elucidated. The gametophytic SI is controlled by a single *S*-locus with multiple alleles, and when one of the two *S*-alleles of the pistil matches those of pollen, the pollen is recognized as self and is rejected [12]. The pistil *S* gene of Rosaceae encodes an extracellular ribonuclease called S-RNase, which acts as a cytotoxin in self-pollen tubes. The pollen *S* gene is involved in detoxifying non-self-S-RNases and encodes an *F-box* gene [12]. The “collaborative non-self-recognition” model was proposed for SI in the Solanaceous plant, *Petunia* [13], where multiple *F-box* genes are involved in pollen specificity, and each targets a subset of non-self-S-RNases for detoxification. The SI of Rosaceae may adopt this “non-self-recognition by a multiple factors” system [12], except for the self-recognition by a single *F-box* gene system in *Prunus* [14]. In the genome databases of *R. chinensis* “Old Blush”, two different regions have been proposed as the *S*-locus [5, 15], both of which are located on the same chromosome 3 within a few Mbp of each other. Vieira *et al*. [15] concluded that their candidate region was the true *S*-locus because its *S-RNase* (hereafter called as *S_C1_ S-RNase*) has a stronger similarity to the *Prunus S-RNase* than the *S-RNase36* identified by Hibrand-Saint Oyant *et al.* [5]. Chen *et al*. [16] identified an *S*-locus-like region in the genome of *Rosa rugosa*, whose the *S-RNase* is orthologous to the *S_C1_ S-RNase* of Old Blush. Du *et al*. [17] identified the *S-RNase* controlling SI in *Fragaria*, which is also more similar to the *S_C1_ S-RNase* rather than *S-RNase36*. These results indicate that the *S_C1_ S-RNase* is the true *S* gene controlling SI in Old Blush. However, validation of the *Rosa S*-locus by pollination experiments has been conducted with only a few individuals [15]. Pollination tests with many individuals are required to confirm the result. Furthermore, Vieira *et al*. [15] identified the *S_C1_ S-RNase* of Old Blush from the genome database of Raymond *et al*. [6] but failed to identify its allelic gene in the other genome database of Hibrand-Saint Oyant *et al.* [5]. The *S-RNase36* identified by Hibrand-Saint Oyant *et al.* [5] has a single intron and weak homology to the *S_C1_ S-RNase*, which has two introns. As the two genome databases of Old Blush represent different haplotypes of chromosome 3 [18], there should be unidentified *S-RNase* in the genome database of Hibrand-Saint Oyant *et al.* [5]. Thus, the first objective of this study is to (i) identify the other allele of *S-RNases* in Old Blush and to confirm the *Rosa S*-locus with strong evidence from a number of pollination experiments.

The second objective of this study is to (ii) test the genetic linkages of the *S*-locus with other loci controlling important ornamental traits. In the chromosome 3 where the *Rosa S*-locus is assumed to be located, there are other important loci controlling valuable traits for ornamental plants, such as CF [19], double flower [20], thornlessness [21], and resistance against black spot disease [22]. These previous studies found strong skewness in the segregation of these traits and hypothesized that their underlying genes are genetically linked with the *S*-locus. The genetic linkage of the *S*-locus and other loci may have important consequences for rose breeding. For example, the CF characteristic is controlled by a single recessive locus on the chromosome 3, and the underlying gene is the non-functional allele (*ksn*) of *RoKSN* [5, 10], a floral repressor gene in roses [23]. Roses homozygous at the non-functional allele *ksn* will be targets in the cross breeding of CF roses. But, if the *ksn* alleles of parental roses are strongly linked with the same *S*-allele, breeding of CF roses with the *ksn*-homozygote will be hampered by SI, where the pistil rejects the pollen with the same *S*-allele linked to *ksn*.

The third objective of this study is to (iii) determine the *S*-allele (*S_C_*) of old Chinese CF cultivars and to clarify the changes in the frequency of rose cultivars with *S_C_* during the history of rose breeding. The *ksn* allele conferring the CF characteristic in modern rose cultivars originated from old Chinese cultivars around 200 years ago [10, 24]. The original Chinese cultivars introducing the CF characteristic into modern roses are thought to be the four China roses, i.e. Slater’s Crimson China, Parsons’ Pink China (Old Blush), Hume’s Blush Tea-scented China, and Parks’ Yellow Tea-scented China [25]. After the introgression of *ksn* from China to Europe, the frequency of rose cultivars carrying the *Sc* should increase but may progressively decrease during the 200 years’ history of rose breeding due to the hybridizations with European cultivars. However, if *Sc* is genetically linked with *ksn*, the strong artificial selection on *ksn* [24] might result in a *ksn*-associated increase in the frequency of *Sc* during the history of rose breeding.

The fourth objective of this study is to (iv) identify candidate genotypes for wild ancestors of old Chinese CF cultivars by using *S_C_* as markers. The introduction of Chinese CF cultivars is one of the most revolutionary events throughout the history of rose breeding [24, 25], and the Chinese cultivars have profound effects on the genetic bases of modern rose cultivars [26]. However, the wild ancestral origin of the old Chinese CF cultivars have not been completely elucidated. Molecular phylogenetic studies have indicated their complex hybrid origins [27–32]. The whole genome sequencing will play a critical role in the elucidation of the hybrid origin of old Chinese cultivars but is too complicated to analyze the massive data for many candidate species. There is also a large intraspecific genetic variation within the species [33, 34], and there might be also natural hybrids between the species. Due to the high genetic divergences of the plant *S*-locus [35], we expect that the *S*-allele specific marker is useful to pinpoint the candidate wild ancestors of old cultivars.

**Figure 1:**
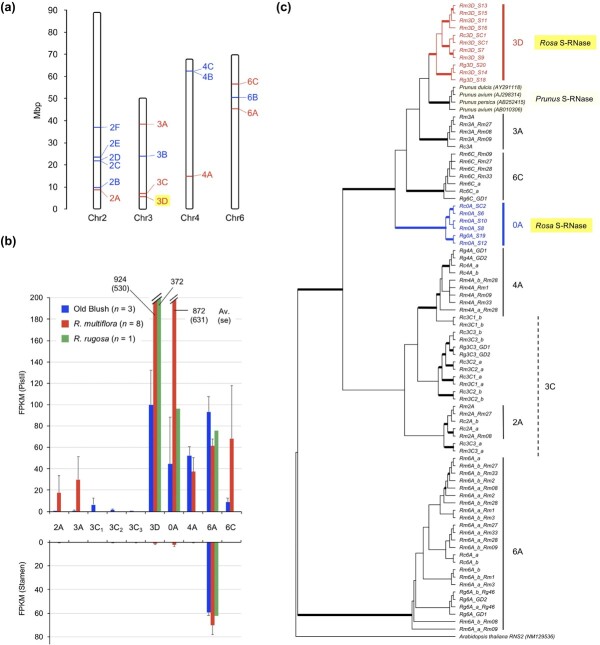
Genome-wide identification and genetic analyses of candidate *S-RNase* genes in the rose. (a) Distribution of the candidate genes in *R. chinensis* “Old Blush” genome. Numbers and genomic positions are shown based on the genome database of Raymond *et al*. [6]. Pistil-expressed genes are shown in red. (b) Pistil and stamen expression levels of the candidate genes in roses. FPKM values were calculated from RNA-seq data of the pistil and stamen. Averages and standard errors of three individuals are shown for *R. chinensis* “Old Blush”. From the transcriptome data of eight *R. multiflora* plants, genes orthologous to the reference genes of Old Blush were identified, and their averages and standard errors of the FPKM values are shown for *R. multiflora*. (c) Molecular phylogenetic tree of the pistil-expressed, candidate genes, including *Prunus S-RNases* as references. The tree was rooted with *T2-RNase* of *Arabidopsis thaliana RNS2* (NM129536) [36]. Bold branches have FastTree support values [37, 38] above 0.95 based on 1000 resamples.

## Results

### Genome-wide identification of candidate S-RNase

We performed a genome-wide search for candidate *S-RNase* genes in the genome databases of Old Blush [5, 6], *R. multiflora* [7], and *R. rugosa* [16, 39] (Supplementary data Table D2) and identified 16 genes located on four chromosomes (Fig. 1a). Of these, seven genes were expressed in the pistil according to the pistil transcriptomes of Old Blush, *R. multiflora,* and *R. rugosa* (Fig. 1b). These candidate genes were named by chromosome number and letter, e.g. 3D and 6A (Fig. 1a). The specific genes of Old Blush, *R. multiflora*, and *R. rugosa* were named with Rc, Rm, and Rg, respectively (Fig. 1c). The putative amino acid sequences were obtained from the cDNA sequences, and a molecular phylogenetic tree of the pistil-expressed, *S-RNase*-like genes was constructed, including the *Prunus* S-RNase (Fig. 1c). The results show that the 3D and 0A genes are the primary candidates for *S-RNase* controlling SI in the rose due to their (i) high expression levels in the pistil (Fig. 1b), (ii) similarity to *Prunus* S-RNase (Fig. 1c), and (iii) high genetic divergences (Fig. 1c).

The 3D *S-RNase* gene in Old Blush named as “*Rc3D_S_C1_*” (Fig. 1c) is the same gene previously identified by Vieira *et al*. [15] as the candidate *S-RNase* in roses. We identified nine additional 3D *S-RNase*-like genes from the genome databases and pistil transcriptomes of *R. multiflora* and *R. rugosa* (Fig. 1c). One of them, named as “*Rg3D_S_18_*^”^, is the same gene previously reported as *S-RNase* (Chr4.718) in the *S*-locus of *R. rugosa* [16]. The high genetic divergence (64.8%) in the 3D *S-RNase* gene agrees well with the assumption that this gene encodes the true *S-RNase* controlling SI in roses (Table 1). One of the 3D genes of *R. multiflora,* named as “*Rm3D_S_C1_*”, is identical to the *Rc3D_S_C1_* of Old Blush, indicating that the *R. multiflora* and Old Blush share the same *S*-alleles (*S_C1_*).

**Table 1:** The identification of candidate genes for the *Rosa S-RNase*. According to the phylogenetic tree (Fig. 1c), four genes (3D, 0A, 3A, 6C) with high similarities to *Prunus S-RNase*s are considered to be candidate *S-RNase* genes

		Candidate *S-RNase* genes^a^			
Species	Data source^b^	3D	0A	3A	6C
*R. chinensis* “Old Blush”	Genome database [5]	*×*	*Rc0A_S_C2_*	*Rc3A*	*Rc6C_a*
	Genome database [6]	*Rc3D_S_C1_*	*×*	*Rc3A*	*Rc6C_a*
*R. rugosa*	Genome database [16]	*Rg3D_S_18_*	*×*	*×*	*Rg6C_GD1*
	Genome database [39]	*Rg3D_S_20_*	*×*	*×*	*×*
	PT of the plant Rg46	*Rg3D_S_18_*	*Rg0A_S_19_*	*×*	*×*
*R. multiflora*	Genome database [7]	*Rm3D_S_15_*	*×*	*Rm3A*	*Rm6C*
		*Rm3D_S_16_*			
	PT of the plant Rm08	*Rm3D_S_7_*	*Rm0A_S_6_*	*Rm3A_Rm08*	*×*
	PT of the plant Rm09	*Rm3D_S_7_*	*Rm0A_S_8_*	*Rm3A_Rm09*	*Rm6C_Rm09*
	PT of the plant Rm27	*Rm3D_S_9_*	*×*	*Rm3A_Rm27*	*Rm6C_Rm27*
		*Rm3D_S_11_*			
	PT of the plant Rm28	*Rm3D_S_13_*	*Rm0A_S_10_*	*×*	*Rm6C_Rm28*
	PT of the plant Rm33	*Rm3D_S_9_*	*Rm0A_S_12_*	*×*	*Rm6C_Rm33*
	PT of the plant Rm3 (tetraploid)	*Rm3D_S_C1_*	*×*	*×*	*×*
		*Rm3D_S_15_*			
	PT of the plant Rm2	*Rm3D_S_C1_*	*×*	*×*	*×*
		*Rm3D_S_15_*			
	PT of the plant Rm1	*Rm3D_S_C1_*	*×*	*×*	*×*
		*Rm3D_S_14_*			
Average pairwise identity of protein sequences		**64.8%**	**62.9%**	97.8%	94.9%
Expression levels in pistil^c^		^***^	^***^	^*^	^**^

a Genes identified in genome databases and pistil transcriptomes. × = unidentified.

b PT = Pistil transcriptome (RNAseq).

c The average FPKM values of *R. multiflora* (Fig. 1b): ^***^, >100; ^**^, 50–100; ^*^10–50.

In the same clade of the *Prunus S-RNase* and the *Rosa* 3D *S-RNase*, there were three other genes (6C, 3A, 0A; Fig. 1c). The 6C and 3A genes are located in different genomic positions from the 3D *S-RNase* (Fig. 1a). Therefore, they are not allelic genes to 3D genes. The 6C and 3A genes have low or undetectable expression levels in pistils (Fig. 1b) and low genetic divergences between alleles (>94% identity; Table 1). One allele of the 6C gene of Old Blush [5] and that of *R. rugosa* [39] are pseudogenes due to frameshift mutations (Supplementary Data Table D2). The 3A gene is homozygous in Old Blush and cannot be identified in two *R. rugosa* genomes. These results indicate that the 6C and 3A genes are not functional *S-RNase* genes.

**Figure 2:**
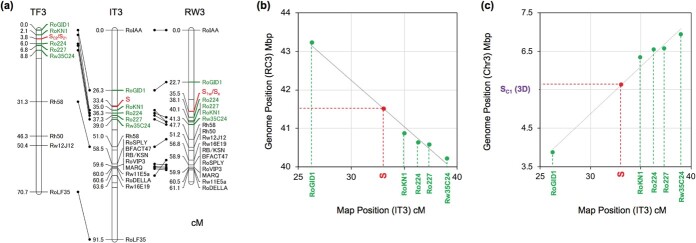
Genetic mapping of candidate *S-RNase* genes identified from the genome database of *R. chinensis* “Old Blush”. (a) Genetic linkage maps of a diploid population FW [19]: The chromosome 3 of the female parent map TF3, the male parent map RW3, and their integrated map IT3. (b)-(c) Estimations of genomic positions of the *S* gene from the map cM of IT3. By using regression lines made by surrounding markers (green), the genomic positions of the *S* gene (red) were estimated in the genome database of (b) Hibrand-Saint Oyant *et al*. [5] and (c) Raymond *et al*. [6].

**Figure 3:**
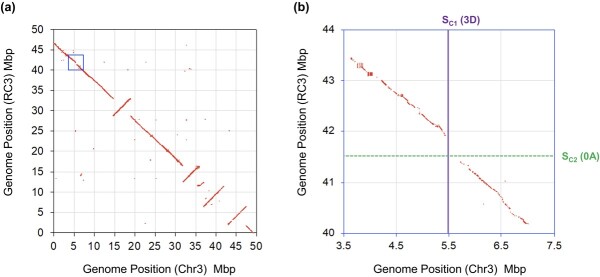
Synteny of the two genome databases of *R. chinensis* “Old Blush” chromosome 3 and the estimated position of *S-RNase*. Dot plots of orthologous genes of two genome databases: Chr3 [6] and RC3 [5] are made using SynMap of CoGe [40]. The CDS of the two genome databases were used to generate the dot plots. (a) Whole chromosome scale, and (b) the focus of the region marked by a blue rectangle. The *S_C1_* (3D) *S-RNase* is located on the 5.5 Mbp position of Chr3, and there is a 500 kbp gap in the synteny at 41.4–41.9 Mbp of RC3. The *S_C2_* (0A) *S-RNase* is estimated to be located on this gap region.

The 0A gene is supposed to be the allelic gene to 3D *S-RNase* in roses. The 0A gene of Old Blush, named as “*Rc0A_S_C2_*”, was identified in the chromosome 0 (i.e. a contig not assigned to one of the seven chromosomes) of the genome database of Hibrand-Saint Oyant *et al.* [5]. We identified five 0A *S-RNase*-like genes from the genome databases and pistil transcriptomes of *R. multiflora* and *R. rugosa* (Fig. 1c). These 0A *S-RNase*-like genes are strongly expressed in pistils (Fig. 1b) and exhibit high genetic divergence (62.9%; Table 1), like the 3D *S-RNase* gene. According to the qPCR analyses of different tissues and developmental stages (Supplementary information 2), the 0A and 3D *S-RNase* genes show pistil-specific expressions. The analyses of pistil transcriptomes of seven diploid *R. multiflora* plants showed that four plants (Rm08, Rm09, Rm28, Rm33) expressed one 3D gene and one 0A gene, while the other three plants (Rm27, Rm2, Rm1) expressed no 0A genes but two 3D genes (Table 1). This also suggests that the 0A and 3D genes are allelic.

To test the hypothesis that the 0A *S-RNase* is allelic to the 3D *S-RNase*, we genetically mapped these genes using a diploid mapping population. The female parent of the diploid mapping population (FW), “The Fairy” (TF), has two 0A genes, one of which is identical to the *Rc0A_S_C2_* (hereafter called as *S_C2_* for simplicity) of Old Blush and the other called *S_21_* (Supplementary information 4). The TF has an *S_C2_* / *S_21_* genotype of the 0A *S-RNase*. In the male parent, RW, we identified one 3D *S-RNase*-like gene and named it *S_1w_* (Supplementary information 4); it has a sequence similar to the *Rc3D_S_C1_* of Old Blush (hereafter called as *S_C1_* for simplicity). The RW has an *S_1w_* / *S_x_* genotype of the 3D *S-RNase* (*S_x_* is an anonymous allele). The 0A and 3D *S-RNase* genes are mapped to homologous positions of chromosome 3 of the female (TF3) and male (RW3) genetic linkage maps, respectively (Fig. 2a). Estimation of genomic position of the *S* gene from map cM of the integrated map (IT3) indicates that the *S* gene is located on 41.5 Mbp of the chromosome 3 (RC3) in the genome database of Hibrand-Saint Oyant *et al*. [5] (Fig. 2b) and is located on 5.6 Mbp of the chromosome 3 (Chr3) of the other genome database of Raymond *et al*. [6] (Fig. 2c). In the latter genome database, the *S_C1_ S-RNase* is located at 5.5 Mbp of the Chr3 close to the estimated position (Fig. 2c).

The two haploid genome databases of *R. chinensis* “Old Blush” are inversely oriented (Fig. 3a). The estimated position of the *S* gene in RC3 (41.5 Mbp) is in an orthologous position to the *S_C1_ S-RNase* position in Chr3 (5.5 Mbp) and is located in a gap of synteny between the two genome databases (Fig. 3b).

### The identification of the S-locus F-box genes

There should be pollen-expressed *F*-box genes flanking the *S-RNase*. This prediction was confirmed by the genome database search and transcriptomes of the stamens of Old Blush (Table 2). From 5.3 to 5.8 Mbp region in Chr3, we identified 12 *F*-box genes and one *S-RNase* (*S_C1_*). Homologous *F*-box genes were identified in 41.3–41.9 Mbp region in RC3, and three other *F*-box genes were also identified in the contig RC0 containing the *S_C2_ S-RNase* (Table 2). All *F-box* genes were expressed in the stamen but not in the pistil. According to the nomenclature of Kubo *et al*. [13], these *F-box* genes were termed as *SLF* or *FBX* (Supplementary Table S7-2). We also confirmed that the similar number of *SLF*s were identified in 500–600 kbp regions flanking to the 3D *S-RNase*-like genes of *R. multiflora* (*S_15_ S-RNase*, *S_16_ S-RNase*) and *R. rugosa* (*S_18_ S-RNase*, *S_20_ S-RNase*) genome databases, and they were expressed in the stamens but not in the pistils (Table S7-1a,b).

**Table 2:** List of candidate *S*-locus *F-box* genes identified by in silico blast search on *R.chinensis* “Old Blush” genome databases

			Genomic position^a^					FPKM^b^	
Type^c^	Gene name	S-genotype	Chromosome	Start	End	Length	Annotation^d^	Stamen	Pistil
FBX12	S_C1__FBX12_pseudo	*S_C1_*	Chr3	5 391 421	5 390 102	1320	NA	Truncated	
SLF1	S_C1__SLF1	*S_C1_*	Chr3	5 434 392	5 433 145	1248	Chr3g0455861	22 (1.6)	1 (0.4)
SLF2	S_C1__SLF2	*S_C1_*	Chr3	5 475 946	5 477 154	1209	Chr3g0455891m	18 (2.9)	1 (0.3)
**S-RNase**	**S**_**C1**_ **_SRNase**	***S***_***C1***_	**Chr3**	**5 498 387**	**5 488 716**	**9672**	**Chr3g0455911m**	**0 (0)**	**100 (32)**
SLF3	S_C1__SLF3	*S_C1_*	Chr3	5 559 472	5 558 219	1254	Chr3g0455931m	6 (1.9)	1 (0.1)
SLF4	S_C1__SLF4	*S_C1_*	Chr3	5 618 754	5 619 992	1239	Chr3g0455991	18 (2.8)	1 (0.2)
FBX1	S_C1__FBX1	*S_C1_*	Chr3	5 722 023	5 720 695	1329	Chr3g0456091	8 (0.8)	0 (0.2)
SLF5	S_C1__SLF5	*S_C1_*	Chr3	5 735 463	5 734 183	1281	Chr3g0456131	13 (2.5)	1 (0.2)
SLF6	S_C1__SLF6	*S_C1_*	Chr3	5 751 338	5 752 606	1269	Chr3g0456171	11 (0.5)	1 (0.2)
SLF8	S_C1__SLF8	*S_C1_*	Chr3	5 803 902	5 805 158	1257	Chr3g0456241	8 (2.4)	1 (0.3)
SLF9	S_C1__SLF9	*S_C1_*	Chr3	5 809 247	5 808 021	1227	Chr3g0456251	20 (2.6)	1 (0.4)
SLF10	S_C1__SLF10	*S_C1_*	Chr3	5 857 662	5 856 391	1272	Chr3g0456291	7 (0.6)	0 (0.3)
SLF11	S_C1__SLF11	*S_C1_*	Chr3	5 880 743	5 879 523	1221	Chr3g0456311	5 (2)	1 (0.2)
FBX4	S_C1__FBX4_pseudo	*S_C1_*	Chr3	5 890 721	5 889 444	1278	NA	Truncated	
FBX2	S_C2__FBX2	*S_C2_*	RC0	27 704 970	27 703 705	1266	NA	5 (0.6)	1 (0.2)
**S-RNase**	**S**_**C2**_ **_SRNase**	***S***_***C2***_	**RC0**	**27 779 508**	**27 755 726**	**23 783**	**NA**	**0 (0)**	**44 (44)**
FBX3	S_C2__FBX3	*S_C2_*	RC0	27 852 918	27 854 177	1260	RC0G0207600	17 (1)	1 (0.4)
SLF7	S_C2__SLF7	*S_C2_*	RC0	27 963 602	27 962 373	1230	RC0G0208100m	4 (0.7)	0 (0.1)
FBX4	S_C2__FBX4	*S_C2_*	RC3	41 290 222	41 291 499	1278	RC3G0342300	16 (2.4)	1 (0.5)
SLF11	S_C2__SLF11	*S_C2_*	RC3	41 310 056	41 311 276	1221	RC3G0342500	5 (0.9)	0 (0.1)
SLF10	S_C2__SLF10	*S_C2_*	RC3	41 344 314	41 345 582	1269	RC3G0343000	5 (0.5)	0 (0.2)
SLF9	S_C2__SLF9	*S_C2_*	RC3	41 348 095	41 349 321	1227	RC3G0343100	18 (3.1)	1 (0.5)
SLF8	S_C2__SLF8	*S_C2_*	RC3	41 352 234	41 350 978	1257	RC3G0343200	9 (2.6)	1 (0.2)
SLF6	S_C2__SLF6_pseudo	*S_C2_*	RC3	41 369 680	41 368 410	1271	RC3G0343500m	Truncated	
SLF5	S_C2__SLF5	*S_C2_*	RC3	41 380 179	41 381 417	1239	RC3G0343800m	16 (2.8)	1 (0.3)
SLF4	S_C2__SLF4_1	*S_C2_*	RC3	41 646 962	41 648 194	1233	RC3G0344900m	4 (0.5)	0 (0)
	S_C2__SLF4_2	*S_C2_*	RC3	41 695 111	41 696 343	1233	RC3G0345200	4 (1.7)	0 (0.1)
SLF3	S_C2__SLF3	*S_C2_*	RC3	41 876 741	41 877 979	1239	RC3G0346100	10 (2.8)	1 (0.2)
SLF2	S_C2__SLF2	*S_C2_*	RC3	41 896 100	41 897 320	1221	RC3G0346200m	13 (2.2)	1 (0.2)
SLF1	S_C2__SLF1	*S_C2_*	RC3	41 907 657	41 908 904	1248	RC3G0346300	15 (3.1)	1 (0.2)
FBX5	S_C2__FBX5	*S_C2_*	RC3	41 932 562	41 931 243	1320	RC3G0346700	4 (1.6)	1 (0.2)
FBX12	S_C2__FBX12	*S_C2_*	RC3	41 965 102	41 966 437	1336	RC3G0347000m	Truncated	

a Genome data source is Raymond *et al*. [6] for *S_C1_*, Hibrand-Saint Oyant *et al*. [5] for *S_C2_*.

b FPKM (Fragments per kilobase of exon per million reads mapped) was calculated from the RNA-seq data of of three individuals of *R. chinensis* “Old Blush” (OB15, OB20, OB75), and averages with standard errors in parenthesis are shown.

c Type was defined as either SLF (*S*-locus linked *F*-box) or FBX, with numbers indicating groups with homologous protein sequences. We categorized the type as *SLF* when at least four *S*-haplotypes out of six (*S_C1_, S_C2_, S_15_, S_16_, S_18_, S_20_*) had the *F-box* genes, whereas we called them FBX only when less than three *S*- haplotypes had the *F-box* genes (Table S7-2).

d NA = No Annotation in original database. The sufflex “m” indicates “modified” annotation of original one.

**Figure 4:**
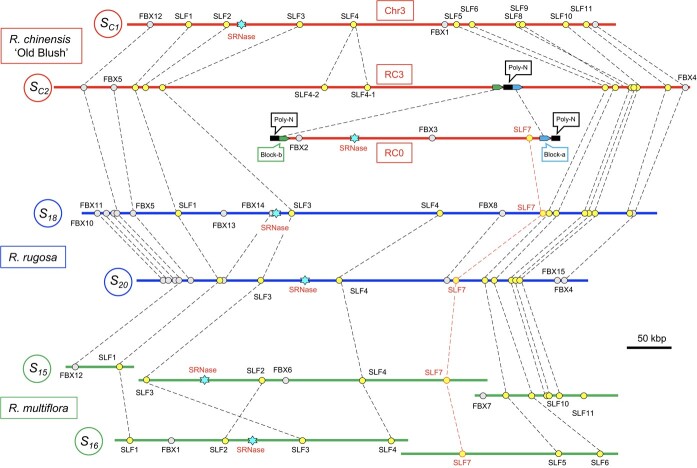
The structure of the *Rosa S*-locus. The putative *S*-locus genomic regions were extracted from genome databases, and the positions of the *S-RNase* and *F*-box (*SLF*, *FBX*) genes were plotted. Homologous *F*-box genes are connected by dotted lines. Data are available in Table 1 for *R. chinensis* “Old Blush” and Supplementary Table S7-1a,b for *R. rugosa* and *R. multiflora.*

Figure 4 shows the structures of the putative *S*-locus regions identified in the *Rosa* genome databases. In the *S*-locus of Old Blush chromosome RC3, there is no *S-RNase* but there is a poly-N region, and its adjacent 10kbp regions (Block-a, Block-b) show close sequence identities with the end regions of the contig RC0. Block-*a* is 11 220 bp with a 98.8% identity between the RC3 and RC0. Block-*b* is 11 382 bp with a 97.5% identity. The *S_C2_ S-RNase* in the contig RC0 appears to be integrated into the poly-N region of RC3. Furthermore, *SLF7* is present in the contig RC0, and its orthologous genes were identified in the *S*-locus regions of *R. rugosa* and *R. multiflora* (*See* also Fig.S7-2 for the alignment). Further, the co-segregations of *SLF5* in RC3 and *S_C2_ S-RNase* in RC0 were tested using the FW mapping population (Supplementary information 8) and found to be perfectly co-segregated (*n* = 97).

### Validation of the S-RNase based S-genotyping by pollination experiments

In order to validate these *S-RNase* genes as the pistil-determinant of SI, pollination experiments on the pairs of diploid roses that share the same *S*-alleles were performed (Table 3). For example, plants with the *S*-genotype *S_C1_* / *S_x_* (where *S_x_* is an anonymous genotype that includes several different *S-RNase* genotypes) were selected and pollinated by pollen collected from the plants with *S_C1_* / *S_y_* (where *S_y_* is another anonymous genotype). After seed maturation, the *S*-genotypes of the seeds were determined, and the genotypic frequency of pollen fertilizing the ovule were calculated. A total 111 seeds were analyzed, and results show that no *S_C1_* pollen fertilized the ovules (i.e. all seeds are derived from pollen with *S_y_* genotypes). These pollination tests with half-compatible pairs were performed for *R. multiflora* plants sharing *S_C1_ S-RNase*-like genes, i.e. Rm3D genes, *S_7_*, *S_9_*, *S_11_*, or *S_13_ S-RNase* (Fig. 1c), providing the same results with *S_C1_* by analyzing total 174 seeds (Table 3). Furthermore, the pollination experiment of TF (*S_C2_* / *S_21_*) with the pollen of Old Blush (*S_C1_* / *S_C2_*) supported the prediction that the *S_C2_* pollen of Old Blush was rejected by the pistil of TF by analyzing 60 seeds (Table 3). The cross pollinations with *R. multiflora* plants sharing *S_C2_ S-RNase*-like genes, i.e. Rm0A genes, *S_6_*, *S_10_*, *S_12_ S-RNase* (Fig. 1c), providing the consistently same results for total 124 seeds (Table 3). These data strongly support the hypothesis that the 3D and 0A *S-RNase* genes are the pistil-determinant of SI in roses.

**Table 3:** Validation of the *S-RNase*-based genotyping by pollination experiments with half-compatible pairs. *See*Supplementary information 5 for full details of the pollination experiments

Recipient plant (♀)			Pollen donor (♂)			Number of seeds (*n*) by *S*-genotype								Number of Pollen Genotypes fertilizing the ovule	
Plant ID^a^	*S*-genotype^b^		Plant ID^a^	*S*-genotype^b^		*S*-genotype	*n*	*S*-genotype	*n*	*S*-genotype	*n*	*S*-genotype	*n*		
M	*S_x_*	*S* _ *C1* _	M	*S* _ *C1* _	*S_y_*	*S_x_ /* *S*_*C1*_	0	*S* _ *C1* _ */ S*_ *C1* _	0	*S_x_ / S_y_*	67	*S* _ *C1* _ */ S_y_*	44	*S* _ *C1* _: *S_y_*	0: 111
Rm09	*S_8_*	*S* _ *7* _	Rm08	*S* _ *7* _	*S_6_*	*S_8_ /* *S*_*7*_	0	*S* _ *7* _ */ S*_ *7* _	0	*S_8_ / S_6_*	10	*S* _ *7* _ */ S_6_*	16	*S* _ *7* _ *: S_6_*	0: 26
M	*S_x_*	*S* _ *9* _	M	*S* _ *9* _	*S_y_*	*S_x_ /* *S*_*9*_	0	*S* _ *9* _ */ S*_ *9* _	0	*S_x_ / S_y_*	26	*S* _ *9* _ */ S_y_*	24	*S* _ *9* _ *: S_y_*	0: 50
M	*S_x_*	*S* _ *11* _	M	*S* _ *11* _	*S_y_*	*S_x_ /* *S*_*11*_	0	*S* _ *11* _ */ S*_ *11* _	0	*S_x_ / S_y_*	27	*S* _ *11* _ */ S_y_*	21	*S* _ *11* _ *: S_y_*	0: 48
M	*S_x_*	*S* _ *13* _	M	*S* _ *13* _	*S_y_*	*S_x_ /* *S*_*13*_	0	*S* _ *13* _ */ S*_ *13* _	0	*S_x_ / S_y_*	23	*S* _ *13* _ */ S_y_*	27	*S* _ *13* _ *: S_y_*	0: 50
TF	*S_21_*	*S* _ *C2* _	OB	*S* _ *C2* _	*S_C1_*	*S_21_ /* *S*_*C2*_	0	*S* _ *C2* _ */ S*_ *C2* _	0	*S_21_ / S_C1_*	35	*S* _ *C2* _ */ S_C1_*	25	*S* _ *C2* _ *: S_C1_*	0: 60
M	*S_x_*	*S* _ *6* _	M	*S* _ *6* _	*S_y_*	*S_x_ /* *S*_*6*_	0	*S* _ *6* _ */ S*_ *6* _	0	*S_x_ / S_y_*	26	*S* _ *6* _ */ S_y_*	23	*S* _ *6* _ *: S_y_*	0: 49
M	*S_x_*	*S* _ *10* _	M	*S* _ *10* _	*S_y_*	*S_x_ /* *S*_*10*_	0	*S* _ *10* _ */ S*_ *10* _	0	*S_x_ / S_y_*	30	*S* _ *10* _ */ S_y_*	17	*S* _ *10* _ *: S_y_*	0: 47
Rm32	*S_13_*	*S* _ *12* _	Rm33	*S* _ *12* _	*S_9_*	*S_13_ /* *S*_*12*_	0	*S* _ *12* _ */ S*_ *12* _	0	*S_13_ / S_9_*	13	*S* _ *12* _ */ S_9_*	15	*S* _ *12* _ *: S_9_*	0: 28

a M = Multiple plants with different *S*-alleles of *S_x_* and *S_y_* were used for experiments. TF = The Fairy, OB = *Rosa chinensis* “Old Blush”. Rm = *R. multiflora*.

b The common *S*-alleles shared by parents are underlined. *S_C1_* is *S-RNase* in Chr3 of the Old Blush genome database [6], and *S_C2_* is the new *S-RNase* identified in RC0 of the Old Blush genome database [5]*.* Other *S*-alleles are *S-RNase* genes identified in *R. multiflora*; *S_7_, S_9_, S_11_,* and *S_13_* are orthologous to *S_C1_*, and *S_6_*, *S_10_*, and *S_12_* are orthologous to *S_C2_* (Fig. 1c). *S_x_* and *S_y_* indicate anonymous *S*-alleles (*S_x_* ≠ *S_y_* in the same row).

### Linkage between the S-locus and important ornamental traits

To consider the effect of SI on the breeding of roses, the degree of genetic linkage between the *S*-locus and the genes underlying important ornamental characteristics (CF and DF) was tested. The *KSN* gene (controlling CF) has a 13.5Mbp distance from the *S*-locus, and the *AP2*-like gene (controlling DF) has a 9.0 Mbp distance from the *S*-locus (Table 4). By using two diploid *F_1_* hybrid populations [19, 41], the recombinant frequencies between the *S*-locus and these genes were estimated. The recombinant frequency between *S* and *KSN* is 20% in RW, and those between *S* and *AP2*-like is 13% in RW and 40% in TF in the FW population. The recombinant frequency between *S* and *AP2*-like is 22% in 93 / 1–119 in the 94 / 1 population. Except for the high recombination between the *S* and *AP2*-like loci in TF, there are significant genetic linkages between the *S* and the *KSN* loci and the *S* and the *AP2*-like loci. This may act as an internal constraint on rose breeding, which is discussed later.

**Table 4:** Linkage between the *S*-locus and important ornamental traits in the rose

		*S*-locus	*AP2-like* (Double-flower)	*KSN* (Continuous-flowering)
Genomic position (bp)^a^		5 488 716 - 5 498 387	14 492 546 - 14 506 765	18 979 892 - 18 989 895
Gene annotation		Chr3g0455911	Chr3g0468481 - Chr3g0468491	Chr3g0473011 Chr3g0473021
Physical distance (Mbp)		0	9.0	13.5
Recombination frequency (%)^b^	RW	–	13% (13 / 97)	20% (19 × 97)
	TF	–	40% (39 / 97)	NA
	93 / 1–119	–	22% (11 / 50)	NA

a Chr3 in the homozygous genome of *R. chinensis* “Old Blush” [6]

b Recombination frequencies in *S* − *AP2* and *S* − *KSN* were estimated in two diploid *F_1_*-hybrid populations: the FW population consists of 97 individuals derived from the pollen donor RW and the seed parent TF [19]. 94 / 1 population consists of 50 individuals derived from the pollen donor 93 / 1–117 and the seed parent 93 / 1–119 [41]. For the two populations, genotypes of *S*, *AP2*, and *KSN* were determined and used to calculate the recombination frequencies. *See*Supplementary information 9 for detailed methods for genotyping.

### Sc-alleles of old Chinese CF cultivars

To identify the Chinese *S*-alleles (*S_C_*) introduced into Europe in the 18^th^ century and examine their fate during the past 200 years’ history of rose breeding, we first analysed the *S*-genotypes of old Chinese CF cultivars (Table 5). The *S*-genotyping showed that the old Chinese cultivars frequently shared *S_C1_* and *S_C2_* alleles with Old Blush (Table 5). Hume’s Blush Tea-scented China has the same *S*-genotype (*S_C1_* / *S_C2_*) as Old Blush. The other CF cultivars, such as Slater’s Crimson China, *R. chinensis* “Mutabilis”, and *R. chinensis*, have either *S_C1_* or *S_C2_*, indicating that they have unidentified *S*-alleles. These unidentified *S_C_*-alleles were amplified with RT-PCR using mRNA prepared from their pistils with degenerate primers for *S-RNase* in roses and sequenced (Fig. S10-1). Partial (*S_C3_*, *S_C4_*) and full (*S_C5_*) *S-RNase* cDNA sequences were obtained and analysed via BLASTX searches against a local protein database of the *Rosa* S-RNase and S-RNase-like proteins, shown in Figure 1c. The BLAST search confirmed that the *S*_*C3*–*5*_ sequences are closest to the 0A S-RNase proteins (*See*Fig. S10–2 for the alignment). As a consequence, *S*-genotypes were estimated as follows: Mutabilis = *S_C1_* / *S_C5_*, *R. chinensis* = *S_C1_* / *S_C4_*, Slater’s Crimson China = *S_C1_* / *S_C3_* or *S_C1_* / *S_C2_* / *S_C3_* (triploid type), and Sanguinea = *S_C2_* / *S_C3_* (Table 5).

**Table 5:** Genotypes of genes controlling important ornamental characteristics in old Chinese rose cultivars. The presence (●) or absence (×) of each gene was determined by PCR. Roses are classified into five types based on the *KSN* genotypes: Type 1 = *ksn^copia^* / *ksn^null^*; Type 2 = *ksn^copia^* / *ksn^copia^*; Type 3 = *ksn^null^* / *ksn^null^*; Type4 = *ksn^copia^* / *KSN^W^*; Type 5 = *KSN^w^* / *KSN^w^*. Roses in Types 1–3 are continuous-flowering (CF), and those in Types 4–5 are once-flowering (OF).

		*S*-allele					*KSN*			*AP2-like*	Phenotype^†^	
Type	Name	*S_C1_*	*S_C2_*	*S_C3_*	*S_C4_*	*S_C5_*	*ksn^copia^*	*ksn^null^*	*KSN^w^*	*ap2*	Bloom	Flower
1	*Rosa chinensis* “Old Blush”	●	●	×	×	×	●	●	×	●	CF	D
	Hume’s Blush Tea-scented China	●	●	×	×	×	●	●	×	●	CF	D
	*Rosa chinensis* “Mutabilis”	●	×	×	×	●	●	●	×	×	CF	S
2	*Rosa chinensis*	●	×	×	●	×	●	×	×	●	CF	D
	Slater’s Crimson China (×2)	●	×	●	×	×	●	×	×	●	CF	D
	Slater’s Crimson China (×3)	●	●	●	×	×	●	×	×	●	CF	D
3	*Rosa chinensis* “Sanguinea”	×	●	●	×	×	×	●	×	×	CF	S
4	*Rosa chinensis* “Single white-eye”	●	×	×	×	×	●	×	●	×	OF	S
	*Rosa chinensis* “Narrow-leaflet”	●	×	×	×	×	●	×	●	●	OF	D
	*Rosa chinensis* “Major”	×	×	●	●	×	●	×	●	●	OF	D
5	Fortune’s Double Yellow	●	×	●	×	×	×	×	●	●	OF	D
	*Rosa odorata* var. *erubescens*	●	×	●	×	×	×	×	●	●	OF	D
	Parks’ Yellow Tea-scented China	×	×	×	×	×	×	×	●	●	OF	D
	*Rosa multiflora* “Carnea”	●	×	×	×	×	×	×	●	●	OF	D
	*Rosa odorata* “Double Light Yellow”	×	●	×	×	×	×	×	●	●	OF	D

†, CF = Continuous-flowering, OF = Once-flowering, D = double-flower, S = single-flower.

The genotyping of the *KSN* gene responsible for the CF characteristic identified five *KSN* genotypes (Table 5). Three *KSN* alleles, i.e. *KSN^W^* (wild allele), *ksn^copia^* (the copia-retrotransposon inserted, non-functional allele) [10], and *ksn^null^* (the deletion, non-functional allele) [5] were distinguished by PCR. New primers were designed to amplify the *ksn^null^* allele (Fig.S10-3). Old Blush, Hume’s Blush Tea-scented China, and Mutabilis are heterozygous for the two non-functional alleles (*ksn^copia^* / *ksn^null^*), whereas Slater’s Crimson China and *R. chinensis* are homozygous for one non-functional allele (*ksn^copia^* / *ksn^copia^*), and Sanguinea is homozygous for the other non-functional allele (*ksn^null^* / *ksn^null^*). Other old Chinese cultivars with once-flowering (OF) behaviour all have the wild *KSN* allele (*KSN^W^*). The *ap2* allele (a transposon-inserted allele) of *AP2*-*like* [5, 11] exists in all old Chinese cultivars with a double flower phenotype (Table 5).

### Introgression of Chinese S-alleles into European roses

**Figure 5:**
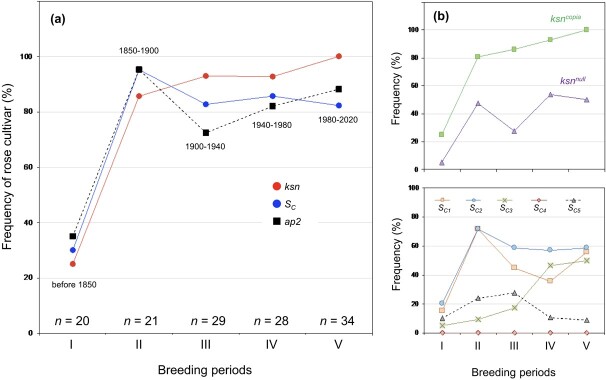
Frequency of rose cultivars with genes that originated from old Chinese cultivars. (a) Frequency of roses carrying CF genes (*ksn*), associated *S* genes (*S_C_*), and double-flower gene (*ap2*). *ksn* indicates the frequency with which roses have *ksn^copia^* and/or *ksn^null^*; *S_C_* indicates the frequency with which roses have *S_C1_*, *S_C2_*, *S_C3_*, *S_C4_*, and/or *S_C5_*. The number of rose cultivars studied are shown above breeding periods. (b) Individual frequency of roses that have *ksn^copia^* or *ksn^null^*, and those roses that have *S_C1_*, *S_C2_*, *S_C3_*, *S_C4_*, or *S_C5_*. Breeding periods: I = before 1850, II = 1850–1900, III = 1900–1940, IV = 1940–1980, V = 1980–2020. The data used to calculate frequencies are available in Supplementary data Table D6.

153 rose cultivars with a variety of breeding histories were genotyped to test for the presence of *ksn* and *ap2* and their associated *S_C_* (*See*Supplementary data Table D6 for the list of cultivars)*_._* The frequencies of rose cultivars with these genes were calculated, along with their breeding periods (Fig. 5). Only 25% of European roses bred before 1850 had either *ksn^copia^* or *ksn^null^*. In particular, roses in the Gallica, Damask, Centifolia, and Alba groups have neither *ksn^copia^* nor *ksn^null^* and show no signs of introgression from Chinese roses (Table S10-1). In contrast, some old roses in the Moss group, such as Mousseline (bred in 1855), had *ksn* alleles, indicating the onset of introgression from Chinese CF roses in this period. These roses also had *S_C1_* or *S_C2_*, demonstrating the parallel introgression of *S_C_* into European roses.

The frequency of roses carrying *ksn* alleles dramatically increased to 86% of roses bred from 1850–1900, gradually increasing to 100% in roses bred from 1980–2020 (Fig. 5). On the other hand, the frequency of roses with *S_C_* reached a peak (95%) in roses bred from 1850–1900, slightly decreasing to 83% in roses bred from 1900–1940, but not changing much until recently (Fig. 5). Due to the small number of samples, these changes in the frequency of rose cultivars after the period II are not statistically significant. The frequency of rose cultivars with *ksn* in the period II (86%) is not significantly different from that in the period V (100%) (Fisher’s exact test, *p* = 0.0507). The frequency of rose cultivars with *Sc* in the period II (95%) is not significantly different from that in the period V (82%) (Fisher’s exact test, *p* = 0.2316).

The *S_C1_* and *S_C2_* are major *S_C_* alleles, and the frequency of roses with the *S_C3_* increased during the 19^th^ century (Fig. 5c). In contrast, roses with the *S_C4_* and *S_C5_* were found with low frequency (<10%) in modern cultivars.

The frequency of roses with *ap2* showed the same trend of frequency changes with breeding periods as *ksn* (Fig. 5), suggesting that the *ap2* allele also originated from Chinese roses. Old European roses with the DF phenotype, such as *Rosa gallica officinalis*, Quatre Saisons, and Chapeau de Napoleon, had no *ap2* allele (Table S10-1), indicating that there is another genetic factor for DF phenotype.

### The wild origin of S_C_-alleles

By screening a total of 95 plants from 25 wild *Rosa* species (Table S11-1) with *S_C_*-specific PCRs, putative wild ancestors of *S*_*C*1–4_ were identified (Table 6). Positive PCR amplifications with *S_C1_-*specific primers were observed for two species, *R. multiflora* and *Rosa brunonii* (Table S11-2). The sequencing of the PCR products shows that *R. multiflora* sequences are 100% identical to the *S_C1_*–sequence of Old Blush, while *R. brunonii* has a 98.4% identity with *S_C1_* (8 SNPs per 500 bp). In the RNA-seq analysis of pistil-expressed genes, it was already found that three *R. multiflora* plants (Rm1, Rm2, Rm3) have *S_C1_ S-RNase* (*= Rm3D_S_C1_* in Fig. 1c), with 100% identical amino acid sequences to Old Blush *S_C1_* (= *Rc3D_S_C1_*). The cDNA sequence of *Rm3D_S_C1_* is also 100% identical to *Rc3D_S_C1_*(Supplementary data Table D4).

**Table 6:** A survey of Chinese wild *Rosa* species with *S_C_*-specific PCRs to identify candidate ancestral species of old continuous-flowering roses

Section	Species	No. of individuals studied^a^	PCR tests for the presence/absence of specific *S-RNase* alleles^b^				
			*S_C1_*	*S_C2_*	*S_C3_*	*S_C4_*	*S_C5_*
Chinenses	*Rosa chinensis* var. *spontanea*	34			★5		
	*Rosa gigantea* ^c^	8		★1	△2	★1	
	*Rosa lucidissima*	2					
Synstylae	*Rosa multiflora*	10	★1				
	*Rosa multiflora* var. *cathayensis*	9			△2		
	*Rosa brunonii*	4	△2				
	*Rosa helenae*	2				△2	
	*Rosa soulieana*	2				△1	
	*Rosa rubus*	4					△1
	*Rosa luciae*	1					
	*Rosa moschata*	2					
	*Rosa anemoneflora*	1					

a Information of sample source is available in Supplementary data Table D6.

b ★ = no. of individuals with 100% identical sequences to CF cultivars, △ = no. of individuals with positive PCR amplifications, while the sequences were not identical to those of CF cultivars. Empty cells = no PCR amplifications for all individuals studied.

c Results of one individual is based on a genome data (SRR6175515).

**Figure 6:**
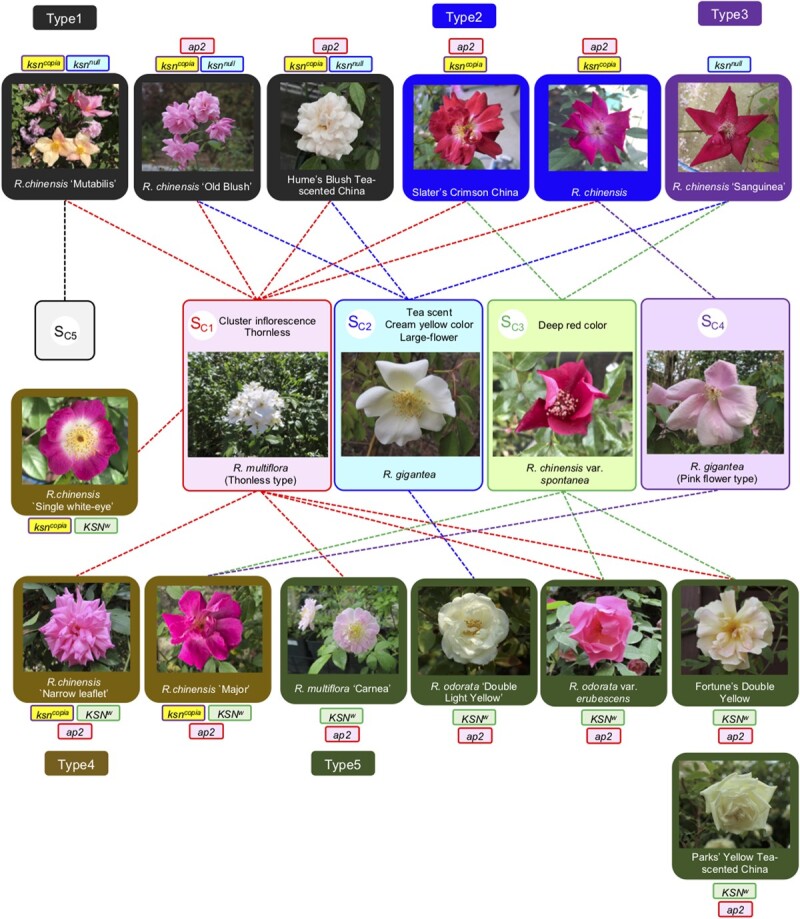
Hypothetical wild ancestral species of old Chinese cultivars inferred from the shares of *S*-alleles. Five *S-*alleles (*S_C1_*,*S_C2_*, *S_C3_*, *S_C4_*, and *S_C5_*) were identified from old Chinese CF cultivars and can be traced to four wild ancestors. The uppermost roses are CF cultivars, and the others are once-flowering cultivars and species. The shares of *S*-alleles are shown by dotted line connections. Cultivars are classified into five types based on their *KSN* genotypes (Table 5), indicated by different colored boxes.

No wild roses were found with positive PCR amplifications from *S_C2_*-specific primers (Supplementary data Table D6). However, it was found that the genome re-sequencing individual of *R. gigantea* (SRR6175515) has 100% identical sequences to *S_C2_ S-RNase* of Old Blush (Fig. S11-2). For the *S_C3_ S-RNase* isolated from Slater’s Crimson China, PCR amplifications with *S_C3_*-specific primers were found for three species, *R. chinensis* var. *spontanea*, *R. gigantea*, and *R. multiflora* var. *cathayensis* (Table 6). Sequencing of the PCR products showed that only *R. chinensis* var. *spontanea* has a 100% identical sequence with *S_C3_* of Slater’s Crimson China (Table S11-3). For the *S_C4_ S-RNase* isolated from *R. chinensis*, PCR amplifications with *S_C4_*-specific primers were found for three species, *R. gigantea*, *Rosa soulieana*, and *Rosa helenae* (Table 6). Sequencing of the PCR products showed that only one plant (pink flower type) of *R. gigantea* has a 100% identical sequence to the *S_C4_* (Table S11-4). For the*S_C5_ S-RNase* isolated from Mutabilis, a positive PCR amplification with *S_C5_*-specific primers was found only in a wild individual of *R. rubus* (Table 6), while the sequencing showed that the PCR product from *Rosa rubus* is not identical to *S_C5_* of Mutabilis (4 SNPs per 221 bp; Fig. S11-3). The putative genetic connections inferred from the shares of *S*-alleles are summarized in Figure 6.

## Discussion

### Identification and validation of the Rosa S-locus

The *Rosa S*-locus was identified and confirmed by clarifying some unresolved issues in previous studies. The genome sequencing of *R. chinensis* “Old Blush” proposed a candidate region of the *S*-locus [5]. This region corresponds to the region of 3C gene (Fig. 1a). We concluded that the 3C gene is not the true *S-RNase* because of (i) the low or undetectable expressions of 3C gene in the pistil (Fig. 1b) and (ii) the low level of sequence divergence between alleles and among individuals (Fig. 1c). Vieira *et al*. [15] analyzed another genome of Old Blush [6] and firstly reported that the 3D gene is the *Rosa S-RNase*. The *S-RNase* “Rchinensis1_3-Rchinensis2_27” in Vieira *et al*. [15] corresponds to the 3D gene (= *S_C1_ S-RNase*) in this study. Vieira *et al*. [15] validated the *S*-*RNase* based on the results of no fruit set of a few individuals pollinated by other individuals with the same *S*-genotypes. This cannot exclude the possibility that inbreeding depression results in no fruit set. We provide strong evidence that the 3D *S-RNase* is the pistil determinant of the SI in roses by genotyping more than 400 seeds produced by a number of half-compatible pairs of roses (Table 3).

This study also identified the 0A gene (= *S_C2_ S-RNase*) in the contig RC0 of the Old Blush genome database (Fig. 1c) and suggested via the mapping approach (Fig. 2, 3) that it is an unidentified allele of the *S_C1_ S-RNase* of Old Blush. The sequence analysis of the *Rosa S*-locus suggested that the contig RC0 can be integrated into a poly-N region of the *S*-locus of RC3 (Fig. 4). The perfect co-segregation of *SLF5* in RC3 and of *S_C2_ S-RNase* in RC0 in a mapping population (*n* = 97; *See*Supplementary information 8) supports this hypothesis. Furthermore, we identified *S_C2_ S-RNase*-like genes from the pistil transcriptomes of wild individuals of *R. multiflora* and *R. rugosa* (*Rm0A* and *Rg0A* genes; Table 1). The pollination experiments using half-compatible pairs of roses that share *S_C2_* or *S_C2_ S-RNase*-like genes indicated that these genes are the pistil-determinants of SI in the rose (Table 3). To determine the precise location of *S_C2_ S-RNase* in the *S*-locus of Old Blush, a BAC library will need to be constructed and sequenced, as shown by Liang *et al*. [42] in their analyses of the *S*-locus structure of *Citrus*.

Based on the identification of *SLF*s flanking to the *S-RNase*, we estimated that the *S*-locus of Old Blush spans approximately 500 kbp (Fig. 4). Chen *et al*. [16] reported that the *S*-locus of *R. rugosa* spanned 667kbp, including one *S-RNase* and 19 *F*-box genes (Table S7-1b). In order to confirm the physical size of the *Rosa S*-locus, further co-segregation analyses of *SLF*s and *S-RNase* are required. The evolutionary divergence analysis of *SLF*s in comparison with *S-RNase* (Fig.S7-3) suggest that the SI of *Rosa* is controlled by the non-self recognition system as previously reported by Vieira *et al.* [15]. In the non-self recognition system, polyploidization will break down the SI [12, 13]. In support to the prediction, we found that the colchicine-induced chromosome doubling of a diploid rose resulted in self-compatible tetraploid (Table S6-1).

### Insights into the breeding of CF roses

The estimations of the degree of genetic linkage between the *S*-locus and the *ksn* controlling CF and the *ap2* controlling DF indicate weak but significant genetic linkages between them. Since the CF is a recessive characteristic, the CF rose (*ksn*-homozygote) cannot be created if *ksn* remains to link with a same *S*-allele. The 20% of recombination frequency (Table 4) suggests that when we cross roses carrying the *ksn* linked with a same *S*-allele, only 20% seedlings are expected to be CF. Therefore, information of *S*-alleles linked with *ksn* is helpful to make the CF rose breeding effective. The SI constraint on breeding would be lower in DF than in CF. Since DF is a dominant characteristic, one *ap2*-allele is enough to make DF roses [5, 11]. Furthermore, we suggest that there had been already other genes or alleles for DF in European roses before the introgression of *ap2* from China (Table S10-1).

Further studies are necessary to confirm and assess the degrees of genetic linkages between the *S*-locus and the loci controlling important ornamental traits, such as CF, DF, thornlessness, and resistance against black spot disease. Our estimation of the degree of genetic linkage depends on a few hybrid populations (Table 4), which cannot represent diverse rose cultivars used for breeding. In addition, the low recombination rates associated with regions adjacent to breakpoints in inversion heterozygotes [43] might result in a stronger genetic linkage of the *S*-locus with *ksn^null^* than with *ksn^copia^*.

As we expected, *S_C_* alleles were introduced into European roses in the 18^th^ century with the CF allele *ksn*. However, the frequency of rose cultivars having *S_C_* remains high (> 80%) in modern cultivars, although it tended to decrease from 95% of cultivars from1850–1900 (Fig. 5a). Due to the fact that many rose cultivars possess the same *Sc* alleles, *S*-genotyping in advance of breeding is helpful for making the diploid rose breeding effective. Furthermore, due to the fact that the two *Sc* alleles *S_C4_* and *S_C5_* are not common (< 10%) in modern cultivars (Fig. 5c), original Chinese CF cultivars of *S_C4_* (*R. chinensis*) and *S_C5_* (Mutabilis) are still useful breeding materials to introduce these rare *S*-alleles into CF cultivars.

As the SI can break down with polyploidization (Table S6-1), breeding between polyploid cultivars may not necessarily consider SI constraints. However, substantial portion (22–35%) of modern cultivars are estimated to be diploid [44, 45], and most wild species and Chinese old cultivars are diploid. Therefore, diploid rose breeding is still an important part of rose breeding.

### Insights into the wild origin of CF roses

We identified the putative ancestors of the *S_C_*-alleles of old Chinese cultivars by screening wild roses with the *S_C_*-specific primers (Table 6). The results confirmed the hybrid origin of the old Chinese cultivars with *R. chinensis* var. *spontanea* and *R. gigantea* in the section *Chinenses* (*Indicae*) and the introgression from *R. multiflora* in the section *Synstylae* (Fig. 6). The genome sequencing of Old Blush reported a sign of introgression from the section S*ynstylae* [6], but which species of the section is involved in the formation of the Old Blush genome is not clarified. Yang *et al*. [30] discussed that *R. multiflora* is a candidate species contributing to the introgression, but there are many other candidates in the section. We investigated nine candidate species in the section *Synstylae* in southwestern China (Table 6) and demonstrated that only *R. multiflora* has an identical *S_C1_*-allele with Old Blush, providing a new evidence for the genetic link between *R. multiflora* and old Chinese cultivars.

## Materials and methods

### Genome-wide identification of candidate S-RNase

By using *S-RNase* of other Rosaceae crops, including *Malus domestica* (AAA79841.1), *Prunus dulcis* (AAL35960.2), *Prunus avium* (BAA36389.1, CAC27788.1), and *Prunus persica* (BAF42768.1) as queries, the Old Blush genome databases [5, 6] were searched using TBLASTN to identify candidate *S-RNase*. All genomic regions with significant hits (E-value <10^–10^), including the regions without any annotations, were listed and manually annotated to infer the coding regions. The other genome databases of *Rosa multiflora* [7] and *R. rugosa* [16, 39] were then searched using the candidate *S-RNase* genes of Old Blush as queries to identify their orthologous genes. For the phylogenetic reconstruction of *S-RNase*-like genes in roses, deduced amino acid sequences were aligned using MUSCLE and then converted back to DNA sequences. The maximum-likelihood phylogenetic tree was constructed from the nucleotide protein-coding sequence alignment by FastTree [37, 38] using the Jukes-Cantor model of nucleotide evolution.

### RNA-seq analysis

To confirm the expression of candidate *S-RNase* in the pistil and to isolate new *S-RNase* alleles, RNA sequencing (RNA-seq) was used. Flower buds one or a few days before anthesis were collected from three individuals of Old Blush, eight individuals of *R. multiflora*, and one individual of *R. rugosa*. Pistils and stamens were collected from the buds and immediately frozen with liquid nitrogen. Total RNAs were extracted using a commercial kit according to the protocol described in Dubois *et al.* [46], and RNA-seq data was obtained through poly-A purification and the 150 bp paired-end method. The RNA-seq reads were mapped to the CDSs of specific genes and whole genome data to calculate FPKM (Fragments per kilobase of exon per million reads mapped) values. To analyze the data of wild *R. multiflora* and *R. rugosa* plants without any specific reference genome databases, the RNA-seq reads were assembled first, followed by the construction of a local database of mRNA sequences, and the database was blasted using the Old Blush genes as queries to identify orthologous genes. The assembly of RNA-seq data was conducted by using the velvet and tadpole algorithm, with default parameters in Geneious Prime 2020.

### Genetic mapping of the new S-RNase in chromosome zero

Genetic mapping of a newly-identified *S-RNase* in the contig that is not assigned to seven chromosomes was performed by using an *F_1_* diploid mapping population (FW) [19, 47, 48]. Three new genetic markers linked to the candidate *S*-*RNase* were added to the previous map to estimate the genomic position of the *S*-gene (Supplementary information 4).

### SLF identification

ORFs longer than 1kbp were extracted from the 1Mbp genomic regions surrounding the *S-RNase* in the Old Blush, *R. multiflora*, and *R. rugosa* genomes, and the *F-box* genes were identified by a Blast search of the ORFs.

### Validation of the S-RNase based S-genotyping by pollination experiments

A total of 20 pairs of diploid roses that share one *S*-allele (i.e. half-compatible) were used for pollination experiments to validate the candidate *S-RNase* gene. Old Blush (*S_C1_* / *S_C2_*), The Fairy (*S_C2_* / *S_21_*), *R. chinensis* “Single white-eye” (*S_C1_* / *S_12_*), and 11 wild individuals of *R. multiflora* were selected based on their *S*-genotypes. Before anthesis, flower buds were bagged to prevent open pollination. Petals and anthers were removed at the balloon stage, and outcross pollen grains were put on the exposed stigma. Pollinations were carried out from April to May in 2018 and 2020, and matured fruits were collected from September to October of the same years. The fruits were opened in the laboratory, the achenes (seeds) were collected, and the *S*-genotypes of the seeds were determined by PCR (Supplementary information 5).

### Linkage between the S-locus and important ornamental traits

Two *F_1_*-diploid mapping populations (FW [19] and 94 / 1 [41]) were used to estimate the recombination frequencies between the *S*-locus and the genes controlling CF (*KSN*) and DF (*AP2-like*). The genotyping of the *S*-locus, *KSN*, and *AP2-like* was performed by PCR (Supplementary information 9), and recombination frequencies were calculated.

### Introgression of the Chinese S-alleles into modern roses

A total of 153 rose cultivars were selected from a wide range of breeding ages and classifications, and young leaves were collected from 20 rose gardens and nurseries in Japan from 2014–2020 (Supplementary data Table D6). DNA was extracted from the young leaves by using the Nucleospin Plant II kit (Macherey-Nagel) according to the manufacturer’s protocol. A PCR was performed to test whether the roses have specific *S*-alleles, mutated-alleles *ksn,* wild-allele *KSN^W^*, or the mutated-allele *ap2* by using EmeraldAmp PCR Master Mix (TaKaRa) with thermal cycling: (1) 2 min of 95°C; (2) 30 s of 95°C; (3) 30 s of T_m_°C; (4) 20–50 s of 72°C; and (5) go back to step (2) 29 times. Information on primer sequence and annealing temperature (T_m_) for PCR is available from Supplementary data Table D1. To identify other Chinese *S*-alleles originally linked with *ksn*, we used three old Chinese CF cultivars, Slater’s Crimson China, Mutabilis, and *Rosa chinensis*, extracted RNAs from their pistils, and performed RT-PCR with primer sets designed on conserved sites of *S-RNase*. Partial or full sequences of three new *S-*alleles were determined and named as *S_C3_*, *S_C4_*, and *S_C5_,* and specific primers were designed for each (Supplementary information 10).

### Wild roses carrying the same S-alleles as old cultivars in China

By screening wild rose species carrying the same *S*-alleles as the old Chinese CF cultivars, the wild ancestral species were inferred. A total of 25 *Rosa* species were surveyed (Table S11-1), with a focus on 13 species in southwestern China (Sichuan and Yunnan Provinces; Fig. S11-1), where a wild type of *R. chinensis*, named *R. chinensis* var. *spontanea*, is naturally distributed. Field collections of wild plants in southwestern China were performed during the flowering seasons of 2018 and 2019. Forty wild plants of seven species were sampled, and *S*-genotyping was performed by PCR. The nucleotide sequences of the PCR products were determined to confirm the results.

## Acknowledgements

This study was supported by the JSPS KAKENHI No. 24688004 and 17H04616. We would like to express our appreciation for Cheng Tingting and Jin Tang in Sichuan University, and Ryosuke Sakamoto in Osaka Institute of Technology, for their helpful support during the fieldwork in China. We are also appreciative of the support from The Rose Culture Institute, Japan, and Drs. Masaki Ochiai and Kunio Yamada at Gifu University, for providing rose materials.

## Contributions

K.K. designed the project and wrote the manuscript. K.K., Y.U., W.L., and G.W. conducted fieldwork in China. K.K., S.M., M.L., and T.D. performed pollination experiments. K.K., T.H., and S.O. analyzed candidate genes, L.H.O., F.F., M.L., and T.D. provided mapping populations. All authors are involved in final manuscript editing.

## Data availability

Sequence data are available in Supplementary data Tables. NGS read data of RNA-seq are available from DDBJ, BioProject PRJDB12320, with accession numbers from DRR321244 to DRR321267.

## Conflict of interests

The authors declare no conflicts of interest associated with this manuscript.

## Supplementary data

Supplementary data is available at *Horticulture Research * online.

## Supplementary Material

Web_Material_uhac155
