# Supplementary material for: The identification of the Rosa S-locus provides new insights into the breeding and wild origins of continuous-flowering roses
Source: Hortic Res. 2022 Oct 1;9:uhac155. doi: 10.1093/hr/uhac155 (PMC9527601; doi:10.1093/hr/uhac155)
Supplement: Web_Material_uhac155 [file web_material_uhac155.zip › certificate_8610560.pdf]

EDITORIAL CERTIFICATE

Authors:

Koji Kawamura et al.

Document title:

The identification of the Rosa S-locus provides new insights into the breeding and wild origins of continuous-flowering roses

Date Issued:

28 May 2022

Cambridge Proofreading LLC

This document certifies that the above manuscript was proofread and edited by Cambridge Proofreading LLC.

This document certifies that the above manuscript was proofread and edited by Cambridge Proofreading Worldwide LLC. The document was edited for proper English language, grammar, punctuation, spelling, and overall style by one or more of our academic editors. The editor endeavoured to ensure that the author's intended meaning was not altered during the review. All amendments were tracked with the Microsoft Word 'Track Changes' feature. Therefore, the authors had the option to reject or accept each change individually.

Kind regards,
Cambridge Proofreading

CAMBRIDGE PROOFREADING LTD
EDITORIAL CERTIFICATE

Cambridge Proofreading Worldwide LLC is a registered company headquartered in Chicago, Illinois, USA with a global presence. All of our editors are native speakers from USA and the UK. Our Certificate of Good Standing can be found in the Illnois state business database by searching our name here.
