# Supplementary material for: The identification of the Rosa S-locus provides new insights into the breeding and wild origins of continuous-flowering roses
Source: Hortic Res. 2022 Oct 1;9:uhac155. doi: 10.1093/hr/uhac155 (PMC9527601; doi:10.1093/hr/uhac155)
Supplement: Web_Material_uhac155 [file web_material_uhac155.zip › Supplementary Information 1.docx]

**Supplementary information 1**

**The identification of the *Rosa* *S*-locus provides new insights into the breeding and wild origins of continuous-flowering roses**

Koji Kawamura^1*^, Yoshihiro Ueda^2,3^, Shogo Matsumoto^4^, Takanori Horibe^4,5^, Shungo Otagaki^4^, Li Wang^6^, Guoliang Wang^7,8^, Laurence Hibrad-Saint Oyant^9^, Fabrice Foucher^9^, Marcus Linde^10^, Thomas Debener^10^

^1^, Department of Environmental Engineering, Osaka Institute of Technology, Japan

^2^, Gifu International Academy of Horticulture, Japan

^3^, Gifu World Rose Garden, Japan

^4^, Graduate School of Bioagricultural Sciences, Nagoya University, Japan

^5^, College of Bioscience and Biotechnology, Chubu University, Japan

^6^, College of Life Sciences, Sichuan University, China

^7^, Jiangsu Provincial Department of Agriculture and Rural Affairs, China

^8^, Agricultural University of Nanjing, China.

^9^, Univ Angers, INRAE, Institut Agro, IRHS, SFR QUASAV, F-49000 Angers, France

^10^, Leibniz Universität, Hannover, Germany

^*^Corresponding author: Koji Kawamura

E-mail: [koji.kawamura@oit.ac.jp](mailto:koji.kawamura@oit.ac.jp)

Tel: +81-(0)6-4300-6848

Affiliation: Department of Environmental Engineering, Osaka Institute of Technology

Address: 5-16-1 Ohmiya, Asahi-ku, Osaka, 535-8585 JAPAN

**Pollination test for self incompatibility and observation of pollen tube growth**

*The pollination experiments and microscopic observations of pollen tube growth confirmed the gametophytic self-incompatibility in two diploid Rosa species, R. chinensis ‘Old Blush’ and R. multiflora.*

**Materials & Methods**

*Pollination test and observation of pollen tube growth*

Self incompatibility (SI) was tested in *R. multiflora* and *R. chinensis* ‘Old Blush’ by pollination experiment and observation of pollen tube growth. Nine wild individuals of *R. multiflora* from a field along the Yodo River in Osaka, Japan, and four pot-cultivated plants of *R. chinensis* ‘Old Blush’ were used for the experiments. Inflorescence shoots were bagged before anthesis to prevent pollination by insects. Anthers were removed from open flowers, and self-pollen was put on the stigmas. More than 20 and eight flowers per individual were pollinated in *R. multiflora* and Old Blush, respectively. Fruit sets and number of seeds (achenes) per fruit were recorded in October 2020.

For observation of pollen tube growth, three wild *R. multiflora* plants were selected. Inflorescence shoots were bagged, and anthers were removed from ten flowers before anthesis at the balloon stage. Self, half-compatible, or full-compatible pollen grains were put on the stigmas of three flowers. The pollinated flowers were sampled 4 days after pollination. For *R. chinensis* ‘Old Blush’, 12 floral buds were randomly selected from four pot-cultivated plants in a laboratory, and anthers were removed from the flowers before anthesis at the balloon stage. Self- or fully compatible pollen grains were put on the stigma of six flowers, and they were sampled 4 and 6 days after pollinations. The pistils of the sampled flowers were immediately fixed, and pollen tubes were observed using fluorescence microscopy. At least three pistils were observed for each flower, and the positions of the pollen tubes in the styles were recorded.

*Protocol for fixation and observation of pollen tubes in the style*

The flowers of *R. multiflora* and *R. chinensis* ‘Old Blush’ were hand pollinated with self, half-compatible, or fully-compatible pollen grains and sampled a few days after the pollination, as described above. The pistils of the sampled flowers were immediately fixed in a FAA solution of formalin : acetic acid : 80% ethanol (1:1:8) for 24 hours at laboratory temperature, transferred to a 50% ethanol solution, and stored at 4℃. The sample was washed with distilled water and placed in 8N NaOH for 24 hours at 65℃ to soften the tissue. The sample was again washed with distilled water and left in 0.1% aniline blue solution (aniline blue/0.1 N K_3_PO_4_) for 4 hours at laboratory temperature to stain callose depositions during pollen tube growth. The stained sample was placed in a petri dish filled with 0.1 N K_3_PO_4_ and dissected with tweezers to take a style with the stigma and the ovary. The trichomes around the ovary were removed to get better visualization of the pollen tubes. Four styles were extracted from each pistil, placed on a glass slide, and compressed by a cover glass. The pollen tubes were observed with a microscope (Nikon Ni-U) with UV-2A filter unit, using UV epifluorescence (Ex 330-380) and long pass filters (DM 400, BA410).

**Results**

The pollination experiments and microscopic observations of pollen tube growth confirmed the gametophytic SI in two diploid *Rosa* species. Self-pollination resulted in 10% and 0% fruit sets in *R. multiflora* (*n* = 219 flowers) and *R. chinensis* ‘Old Blush’ (*n* = 34 flowers), respectively. In 60% of pistils after 4 days of outcross pollination in *R. multiflora*, pollen tubes reached ovules while with self-pollination, pollen tubes reached the ovules of only 1% of pistils (**Table S1-1**). In *R. chinensis* ‘Old Blush’, pollen tubes reached basal parts in 88% of outcrossing pistils after 4 days and fertilized the ovules in 79% of pistils after 6 days, but no pollen tubes reached the ovules in pistils with self-pollination (**Table S1-1**).


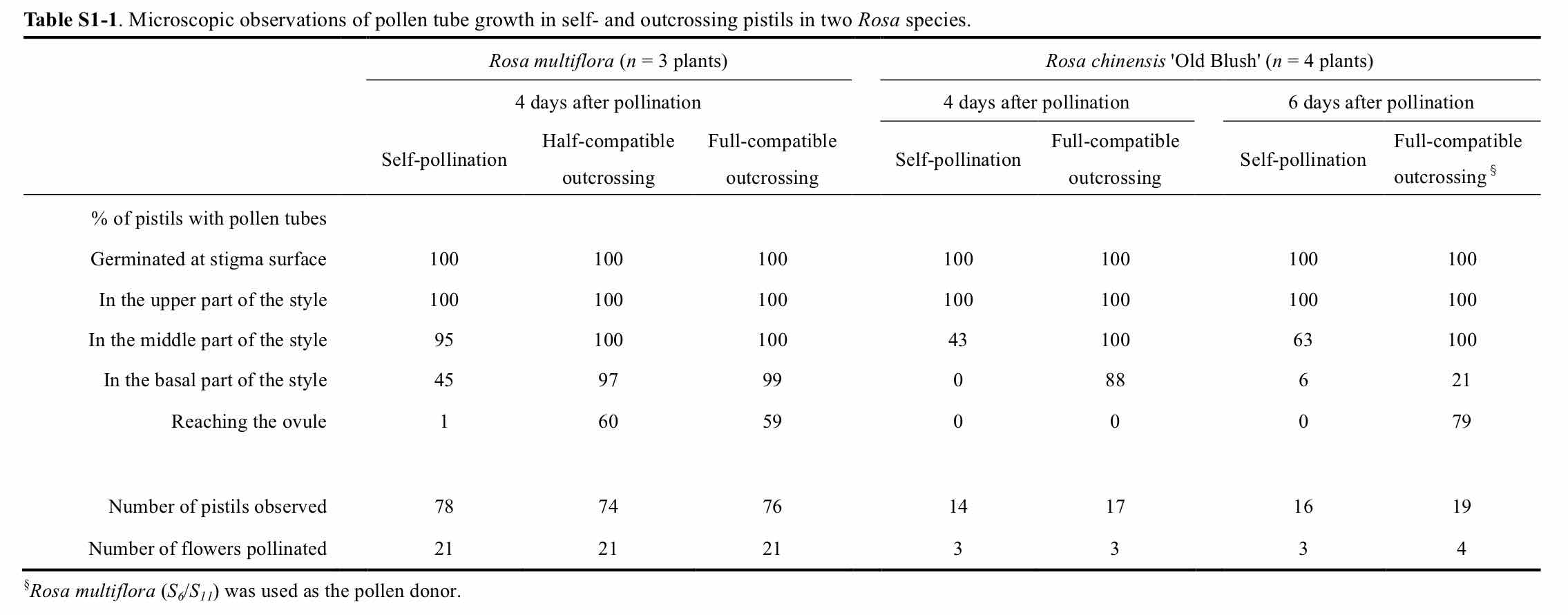


Typical examples of microscopic image of pollen tube in the pistil are shown below.


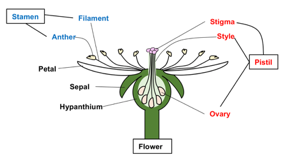


**Figure S1-1.** Structure of rose flower.

**
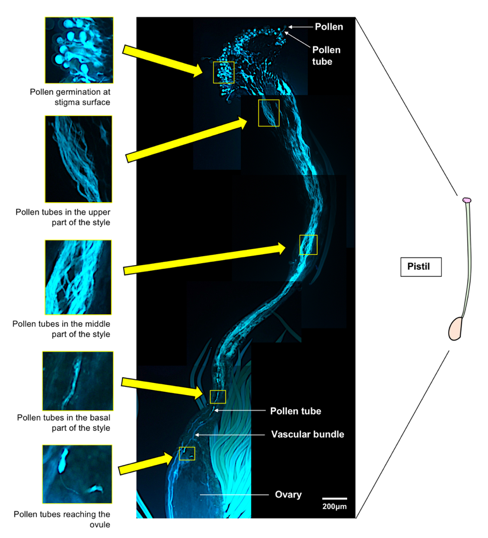
**

**Figure S1-2.** An example of a fluorescence microscopic image of a pistil, collected from a flower of *Rosa multiflora*, Rm13 (*S_6_*/*S_11_*), pollinated by the pollen grains of Rm08 (*S_6_*/*S_7_*).

**
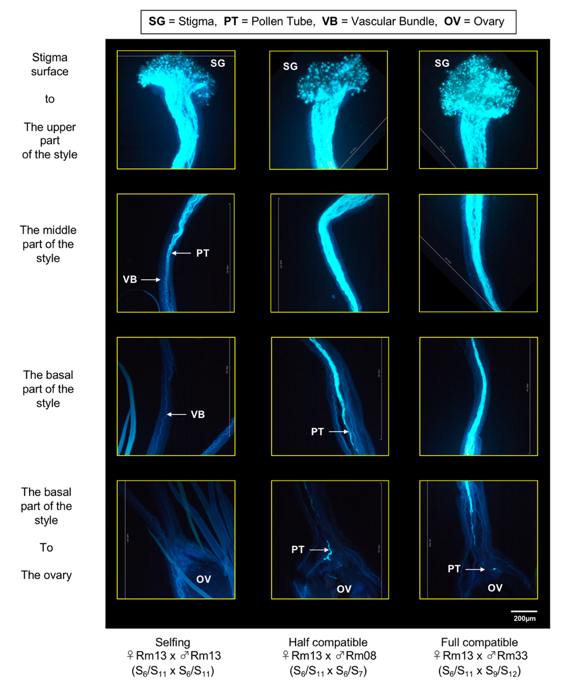
**

**Figure S1-3.** Typical images of the pistils of from individual of *R. multiflora* (Rm13) flowers pollinated by self, half-compatible, or full-compatible pollen grains.

**
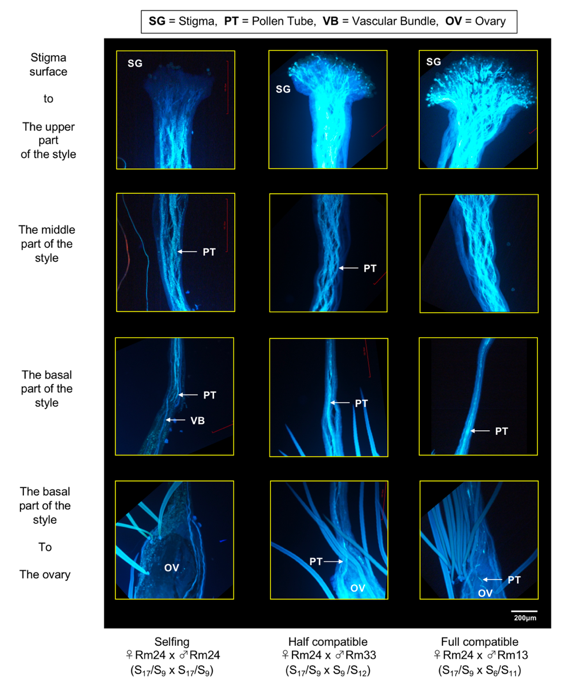
**

**Figure S1-4.** Typical images of the pistils from individual of *R. multiflora* (Rm24) flowers pollinated by self, half-compatible, or full-compatible pollen grains.

**
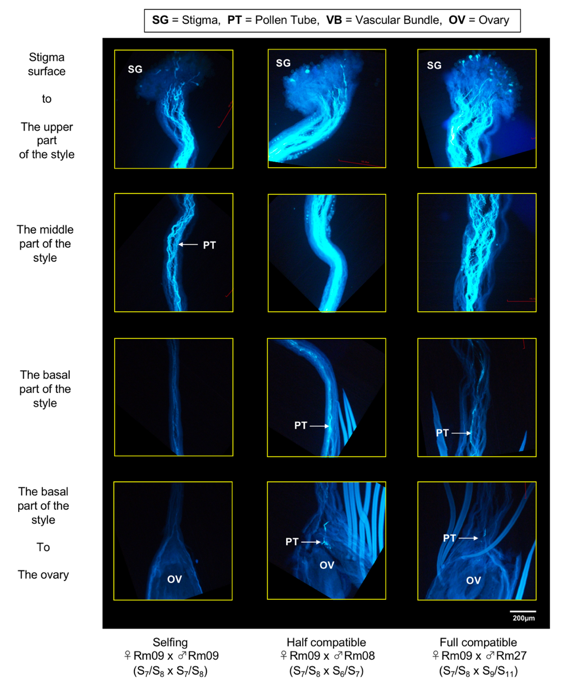
**

**Figure S1-5.** Typical images of the pistils from individual *R. multiflora* (Rm09) flowers pollinated by self, half-compatible, or full-compatible pollen grains.

**
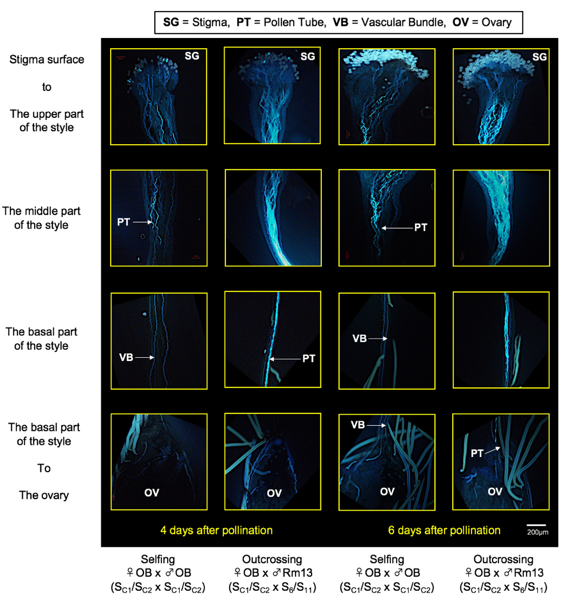
**

**Figure S1-6.** Typical images of *R. chinensis* ‘Old Blush’ flowers pollinated by self or full-compatible pollen grains. OB = Old Blush.
