# Supplementary material for: The identification of the Rosa S-locus provides new insights into the breeding and wild origins of continuous-flowering roses
Source: Hortic Res. 2022 Oct 1;9:uhac155. doi: 10.1093/hr/uhac155 (PMC9527601; doi:10.1093/hr/uhac155)
Supplement: Web_Material_uhac155 [file web_material_uhac155.zip › Supplementary Information 10.docx]

**Supplementary information 10**

**The identification of the *Rosa* *S*-locus provides new insights into the breeding and wild origins of continuous-flowering roses**

Koji Kawamura^1*^, Yoshihiro Ueda^2,3^, Shogo Matsumoto^4^, Takanori Horibe^4,5^, Shungo Otagaki^4^, Li Wang^6^, Guoliang Wang^7,8^, Laurence Hibrad-Saint Oyant^9^, Fabrice Foucher^9^, Marcus Linde^10^, Thomas Debener^10^

^1^, Department of Environmental Engineering, Osaka Institute of Technology, Japan

^2^, Gifu International Academy of Horticulture, Japan

^3^, Gifu World Rose Garden, Japan

^4^, Graduate School of Bioagricultural Sciences, Nagoya University, Japan

^5^, College of Bioscience and Biotechnology, Chubu University, Japan

^6^, College of Life Sciences, Sichuan University, China

^7^, Jiangsu Provincial Department of Agriculture and Rural Affairs, China

^8^, Agricultural University of Nanjing, China.

^9^, Univ Angers, INRAE, Institut Agro, IRHS, SFR QUASAV, F-49000 Angers, France

^10^, Leibniz Universität, Hannover, Germany

^*^Corresponding author: Koji Kawamura

E-mail: [koji.kawamura@oit.ac.jp](mailto:koji.kawamura@oit.ac.jp)

Tel: +81-(0)6-4300-6848

Affiliation: Department of Environmental Engineering, Osaka Institute of Technology

Address: 5-16-1 Ohmiya, Asahi-ku, Osaka, 535-8585 JAPAN

***Introgression of Chinese S_C_-alleles into modern roses***

*A list of studied rose cultivars with phenotypic information is shown. The methods for primer design, PCR, and ploidy-level estimation are described. Genotyping results are explained in detail with the name of rose cultivars.*

**Materials & Methods**

*Primer design for specific amplification of S_C3_, S_C4_, and S_C5_*

Degenerate primers designed on conserved sites (S2_e2_F2, S2_e3_R3, and S2_e2_F1, S2_e3_R2) were used to amplify *S_C3_ and S_C4_* from cDNA prepared from RNA extracted from the pistils of the roses. Specific primers were designed inside the degenerate primers. The full cDNA sequence of the *S_C5_* *S-RNase* of *R. chinensis* ‘Mutabilis’ was first constructed from genome re-sequencing data (SRR6175518), and subsequently confirmed by PCR of cDNA prepared from *R. chinensis* ‘Mutabilis’. Specific primers were designed on exon3. Primer information is available from Supplementary data **Table D1**.


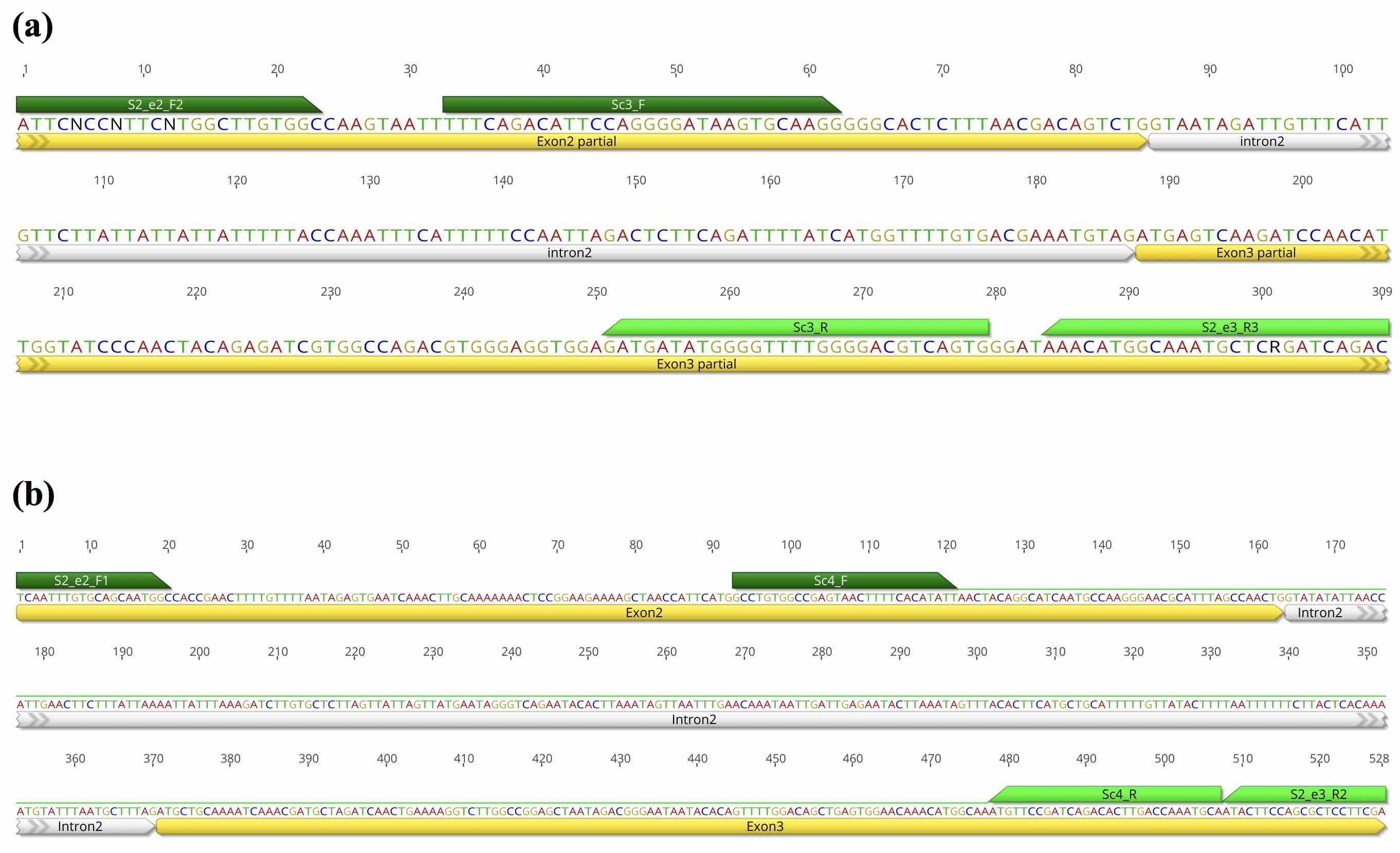


**(c)**


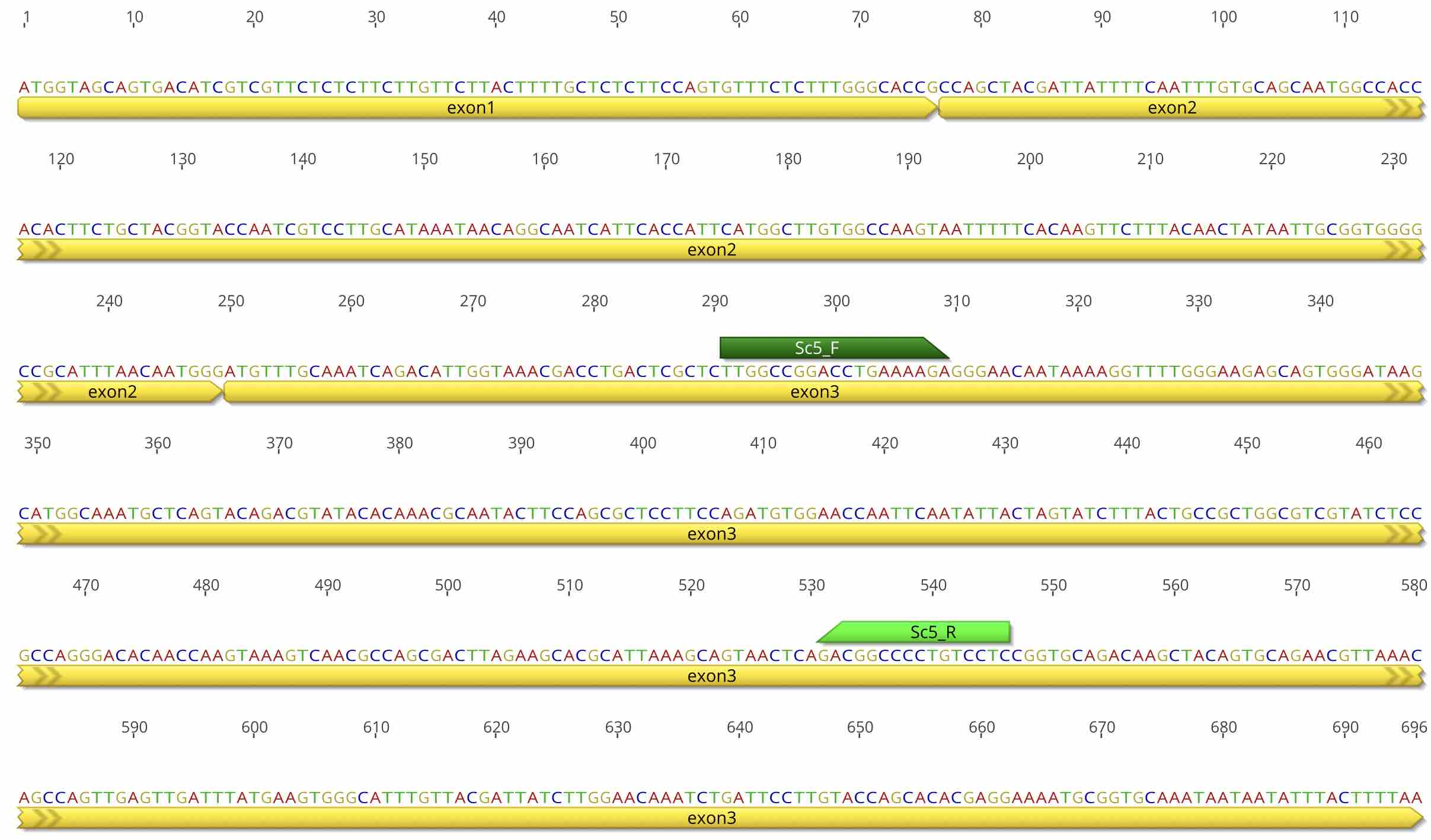


**Figure S10-1**. Partial or full sequences of *S-RNase* of Chinese old CF cultivars. **(a)** Partial sequence of *S_C3_* *S-RNase* isolated from Slater’s Crimson China. **(b)** Partial sequence of *S_C4_* *S-RNase* isolated from *R. chinensis*. **(c)** Full cDNA sequence of *S_C5_* *S-RNase* isolated from *R. chinensis* ‘Mutabilis’. Sequence data are available from **Table D7**.

In order to confirm the isolated new *S_C3-5_ S-RNase* genes are homologous to 3D*_,_* 0A *S-RNase* in Old Blush, blastx search was performed on the S-RNase and other non S-RNase proteins identified in Old Blush genome databases (**Fig.1** in main text) using the partial (*S_C3-4_*) and full (*S_C5_*) cDNA sequences as queries. The results show that all of *S_C3-5_ S-RNase* genes are closest to 0A *S-RNase.* **Fig.S10-2** shows an alignment of the cDNA sequences of *S_C3-5_ S-RNase* genes with other 0A *S-RNase* genes isolated from Old Blush (Rc0A_S_C2_), *R.rugosa* (Rg0A_S_19_), and *R.multiflora* (Rm0A_S_6_, S_8_, S_10_, S_12_). Conserved sites in Rosaceae S-RNase (Ushijima et al., 1998) are shown below the consensus sequences.

*
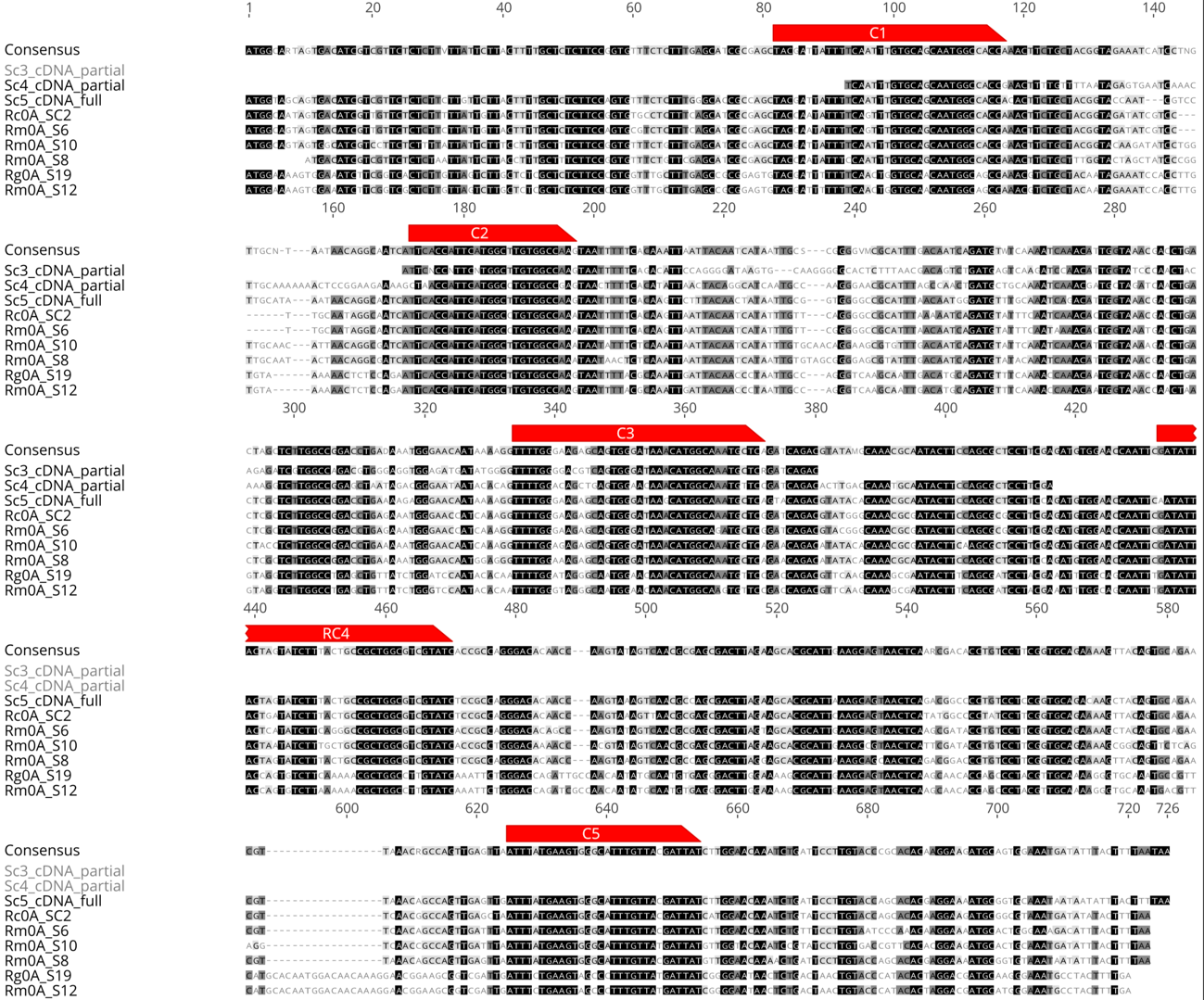
*

**Figure S10-2.** Alignment of S_C3-5_ *S-RNase* cDNA sequences togher with 0A *S-RNase* genes. Conserved sites in Rosaceae S-RNase (Ushijima *et al*., 1998) are shown above the consensus sequences.

*Primer design for specific amplification of ksn^null^*

**Figure S10-3** shows chromosome-scale inversion and deletion of *KSN* in *Old Blush.* There is a large inversion from the 28.8 to the 33.1 Mbp segment of RC3 corresponding to the segment of the 14.7 to the 19.0 Mbp of Chr3 (**Fig.S10-3a**). In a boundary region of the inversion, there is the copia retrotransposon-inserted *KSN* (*ksn^copia^*) at the 19.0 Mbp of Chr3, which is annotated by two separate genes (Chr3g0473011 and Chr3g0473021) (**Fig.S10-3b**). The dot plot shows that there is no sequence in RC3 corresponding to the region of *ksn^copia^*, indicating the deletion of *KSN* from RC3 (= *ksn^null^*). Primers for the *ksn^null^* allele were designed upon the boundary region of inversion in RC3 (from 33,087,543 to 33,088,103) (**Fig. S10-3c**). Primer information is available from Supplementary data **Table D1**.

**
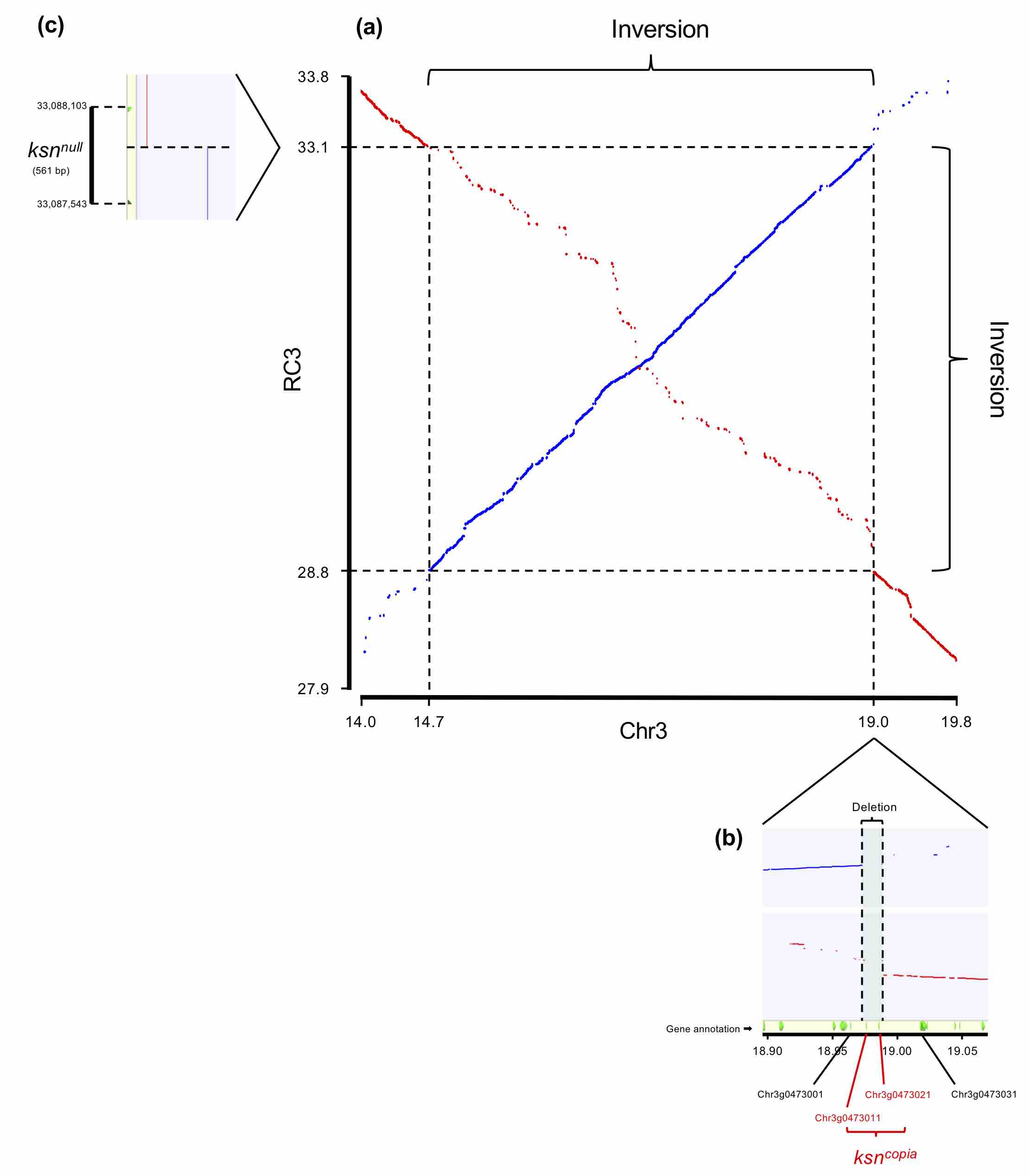
**

**Figure S10-3**. Chromosome-scale inversion and deletion of *KSN* in *R. chinensis* ‘Old Blush’*.* **(a)** Dot plot of inverted part of chromosome 3 in *Rosa chinensis* ‘Old Blush’. Horizontal axis shows Chr3 (14.0-19.8 Mbp) of the genome of Raymond *et al.* (2018), and vertical axis is RC3 (27.9-33.8 Mbp) of the genome of Hibrand-Saint Oyant *et al.* (2018). The genomic data of Chr3 and RC3 was aligned by LASTZ ver 1.02 (Harris 2007), with step length 20. Blue and red dots show forward and reverse alignment, respectively.

*Ploidy level estimation*

Ploidy level was estimated for some roses using the following methods:

1. Young leaves (1.0～1.3 g) were cut and placed in a petri dish on ice, with a 5～6 ml chopping buffer^†^.
2. The chopped buffer was filtrated with a 40 µm cell strainer.
3. The filtrate was centrifuged at 300 rcf, 4℃ for 5 min, the supernatant was removed, and the precipitate was resolved in a new 500 µL chopping buffer.
4. A 198 µL of the sample solution was stained with 2 µL of DAPI solution (1 mg/mL dissolved in H_2_O) for 5 min at 4℃ under darkness.
5. The sample solution was mixed with a standard solution made from *R. chinensis* ‘Old Blush’.
6. Peak fluorescence intensity of DAPI-stained DNA was measured by VL-1 channel (415-465nm with 405-nm excitation laser) of Attune flow cytometer (Thermo Fisher Scientific). Relative fluorescence intensity of sample to standard (diploid Old Blush) was calculated as ‘Ploidy level’.
7. Based on the ploidy level, ploidy (×2, 3, 4, ...) was determined.

^†^, 500mL of chopping buffer contains:

- 5 mL of Triton X-100 (1 %)
- 4.88 mL of 2-Mercaptoethanol（0.14 M)
- 3.15 g of Na_2_SO_3_（0.05 M)
- 3.03 g of Tris(hydroxymethyl)aminomethane (0.05 M)
- pH was adjusted to 7.5 by HCl.

**Results & Discussion**

*Genotyping results output format in Supplementary data* ***Table D6***

Genotyping results are available from **Table D6** and scored as the presence (●) or the absence (×) of specific alleles. Empty cells indicate no PCR test.


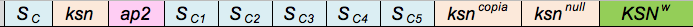


*S_C_* ● = Presence of either *S_C1_*, *S_C2_*, *S_C3_*, *S_C4_*, or *S_C5_*

× = Absence of all *S_C1_*, *S_C2_*, *S_C3_*, *S_C4_*, and *S_C5_*

*ksn* ● = Presence of either *ksn^copia^* or *ksn^null^*

× = Absence of both *ksn^copia^* and *ksn^null^*

*KSN^W^* ● = Presence of wild *KSN* allele without insertion

● (LTR) = Presence of long terminal repeat (LTR) inserted *KSN* allele

× = No PCR amplification

**Table S10-1** shows the genotyping results of old European cultivars extracted from **Table D6**. The presence of *ksn* allele (●) in some cultivars in the Moss group indicates the onset of introgression from China. The absence of the *ap2* allele in old European roses with double flower phenotypes suggests that their double flower phenotypes have a different genetic origin from Chinese cultivars.

**Table S10-1**. Genotyping results of old European cultivars. Data is extracted from **Table D6**.


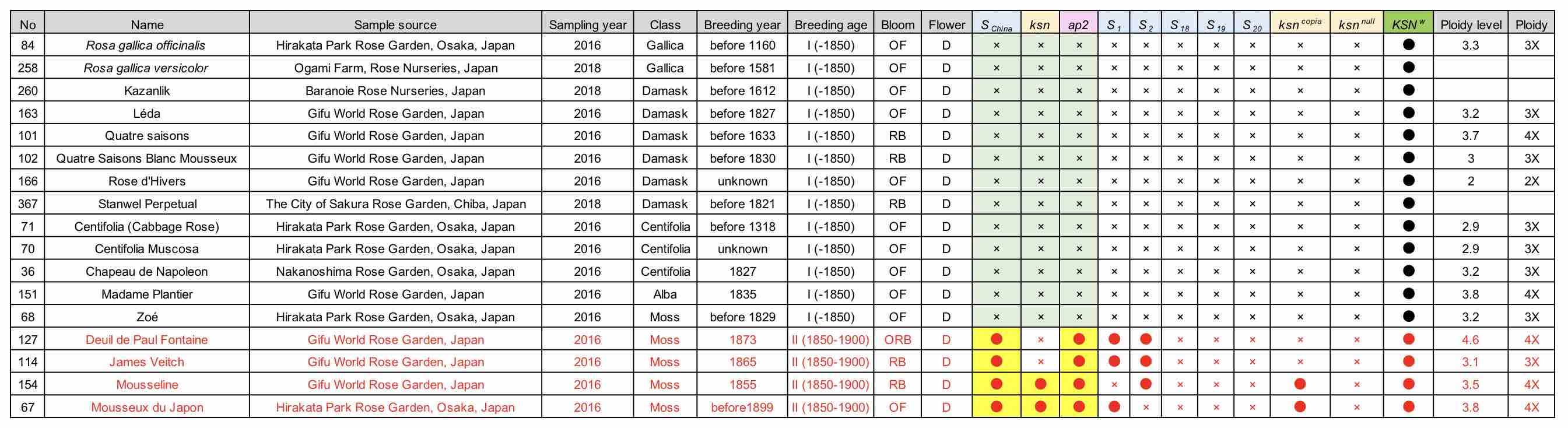


● = presence, × = absence of gene. Red-colored cultivars show introgression of the Chinese *S*-alleles, *ksn*, or *ap2*. *S_C_* indicates the presence of at least one Chinese *S*-alleles (*S_C1_*, *S_C2_*, *S_C3_*, *S_C4_*, or *S_C5_*), and *ksn* indicates the presence of either *ksn^copia^* or *ksn^null^*.

**References**

Harris, R.S. *Improved pairwise alignment of genomic DNA*. PhD thesis, Penn State University, Computer Science and Engineering (2007).

Hibrand Saint-Oyant L *et al*. A high-quality genome sequence of Rosa chinensis to elucidate ornamental traits. *Nature Plants* **4**: 473-484 (2018).

Raymond O. *et al*. The Rosa genome provides new insights into the domestication of modern roses. *Nature Genet.* **50**: 772-777 (2018).

Ushijima, K. *et al.* Cloning and characterization of cDNAs encoding S-RNases from almond (*Prunus dulcis*): primary structural features and sequence diversity of the S-RNases in Rosaceae. *Mol. General Genet.* **260**, 261-268 (1998).
