# Supplementary material for: The identification of the Rosa S-locus provides new insights into the breeding and wild origins of continuous-flowering roses
Source: Hortic Res. 2022 Oct 1;9:uhac155. doi: 10.1093/hr/uhac155 (PMC9527601; doi:10.1093/hr/uhac155)
Supplement: Web_Material_uhac155 [file web_material_uhac155.zip › Supplementary Information 11.docx]

**Supplementary information 11**

**The identification of the *Rosa* *S*-locus provides new insights into the breeding and wild origins of continuous-flowering roses**

Koji Kawamura^1*^, Yoshihiro Ueda^2,3^, Shogo Matsumoto^4^, Takanori Horibe^4,5^, Shungo Otagaki^4^, Li Wang^6^, Guoliang Wang^7,8^, Laurence Hibrad-Saint Oyant^9^, Fabrice Foucher^9^, Marcus Linde^10^, Thomas Debener^10^

^1^, Department of Environmental Engineering, Osaka Institute of Technology, Japan

^2^, Gifu International Academy of Horticulture, Japan

^3^, Gifu World Rose Garden, Japan

^4^, Graduate School of Bioagricultural Sciences, Nagoya University, Japan

^5^, College of Bioscience and Biotechnology, Chubu University, Japan

^6^, College of Life Sciences, Sichuan University, China

^7^, Jiangsu Provincial Department of Agriculture and Rural Affairs, China

^8^, Agricultural University of Nanjing, China.

^9^, Univ Angers, INRAE, Institut Agro, IRHS, SFR QUASAV, F-49000 Angers, France

^10^, Leibniz Universität, Hannover, Germany

^*^Corresponding author: Koji Kawamura

E-mail: [koji.kawamura@oit.ac.jp](mailto:koji.kawamura@oit.ac.jp)

Tel: +81-(0)6-4300-6848

Affiliation: Department of Environmental Engineering, Osaka Institute of Technology

Address: 5-16-1 Ohmiya, Asahi-ku, Osaka, 535-8585 JAPAN

***Wild roses carrying the same S_C_-alleles as old Chinese cultivars***

*Sample location of wild roses and sequencing results of PCR products of S_C_-specific primers are described.*

**Materials & Methods**

We performed *S_C_* specific PCR (described in Supplementary information 10) on a total of 95 plants from 25 *Rosa* species (**Table S11-1**), with a focus on wild species in southwestern China (Sichuan and Yunnan Provinces), where the wild type of *R. chinensis*, named *R. chinensis* var. *spontanea,* is naturally distributed.

Fieldworks in southwestern China was conducted in 2018 and 2019 during the flowering seasons. At the three sites (Pingwu, Dujiangyan, Emeishan) in Sichuan province and three sites (Lijian, Dali, and Kunming) of Yunnan province (**Fig. S11-1**), wild rose species are sampled for the DNA analyses. List of sampled rose plants and species is available from Supplementary data **Table D6**.


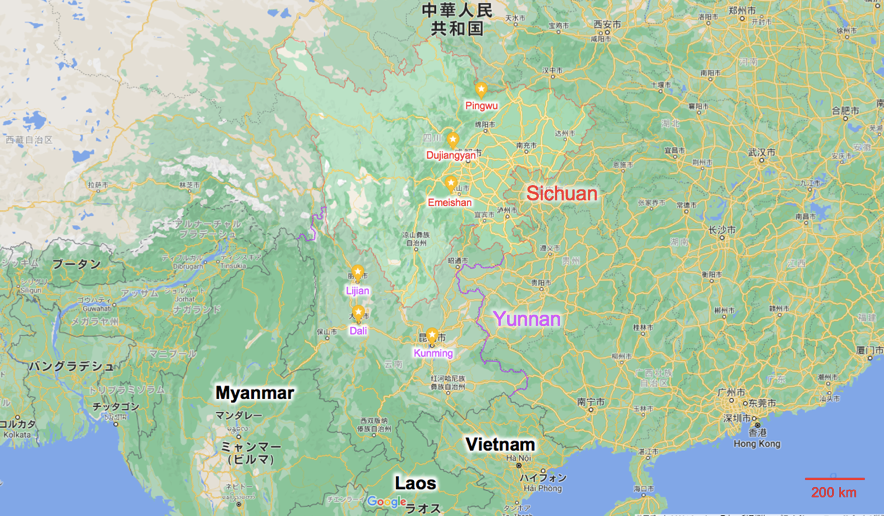


**Figure S11-1.** Locations of the wild rose survey in southwestern China, Sichuan and Yunnan Provinces. Map data was extracted from Goolgle Japan©.

**Table S11-1**. A list of wild *Rosa* species studied.

**Results & Discussion**

*Wild ancestral origin of S_C1_ , S_C2_ (Old Blush)*

**Table S11-2** shows the list of wild species and old cultivars with positive PCR amplifications of *S_C1_*-specific primers. Except for *R. brunonii* (Nos. 600, 607) with eight SNPs per a total 500 bp sequence, *R. multiflora* (Thornless) collected in Lijian and some old cultivars have 100% identical sequences to the sequence of Old Blush.

**Table S11-2.** Wild species and old cultivars with positive PCR amplifications of *S_C1_*-specific primers. Data were extracted from Supplementary data **Table D6**.


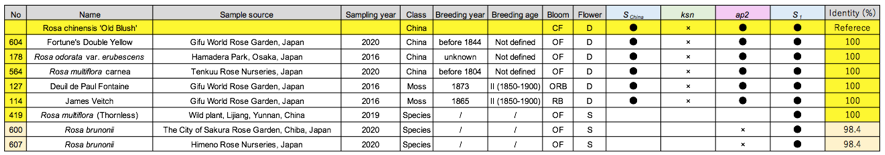


Identity (%) indicates nucleotide sequence identity per a total 188 bp sequence of the PCR products to the *S_C3_* original sequence of Slater’s Crimson China (No.613).

**Figure S11-2** shows the result of mapping NGS reads of the *R. gigantea* genome re-sequencing data (SRR6175515) onto the *S_C2_* *S-RNase*. The entire region of *S_C2_* *S-RNase* (23,924 bp) of Old Blush was covered by 100% identical sequences (with no gap), indicating that this individual of *R. gigantea* has the same *S_C2_* allele with Old Blush.


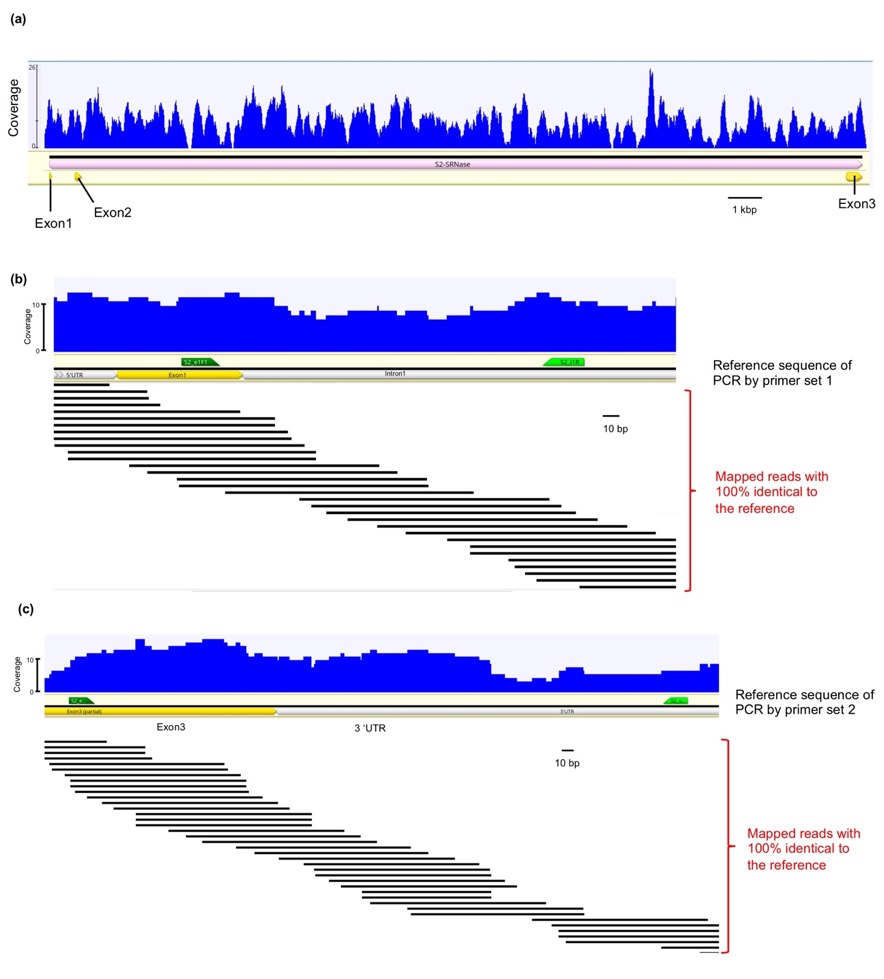


**Figure S11-2.** Coverage of the mapping NGS reads of the *R. gigantea* genome re-sequencing data (SRR6175515) onto the *S_C2_* *S-RNase*. Only reads with 100% identical sequences (with no gap) to the reference were mapped. **(a)** Total 1,369 of 133,018,374 reads were mapped to the entire region of *S_C2_* *S-RNase* (23,924 bp) of Old Blush. The reference sequence of *S_C2_* *S-RNase* was obtained from RC0: 27,755,725-27,779,508 in the genome database of Hibrand-Saint Oyant *et al.* (2018). The annotations (Exon) are based on the RNA-seq of the pistil of Old Blush. Detailed view of the region amplified by **(b)** PCR with primer set 1 (S2_e1F, S2_i1R) and **(c)** PCR with primer set 2 (S2_e3F, S2_u3R). Both regions were covered by 100% identical reads mapped.

*Wild ancestral origin of S_C3_ (Slater’s Crimson China)*

**Table S11-3** shows the list of wild species and old cultivars with positive PCR amplifications of *S_C3_*-specific primers. Wild species *R. chinensis* var. *spontanea* has *S_C3_* *S-RNase* identical to those of old cultivars, Slater’s Crimson China and *R. chinensis* ‘Sanguinea’.

**Table S11-3.** Wild species and old cultivars with positive PCR amplifications of *S_C3_*-specific primers. Data were extracted from Supplementary data **Table D6**.


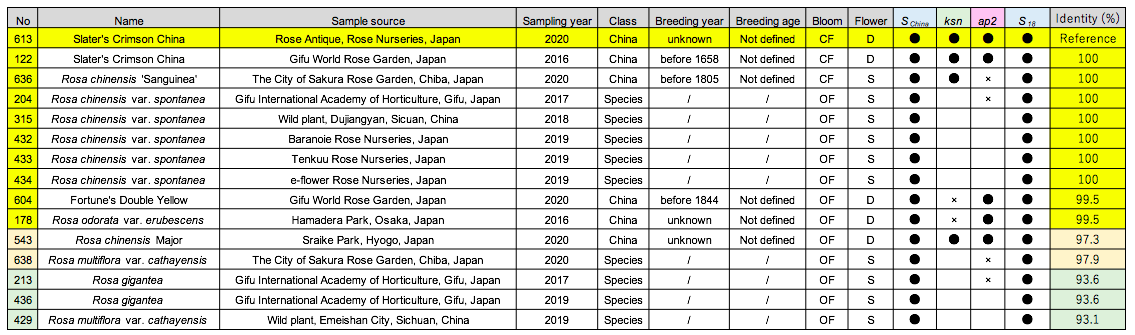


Identity (%) indicates nucleotide sequence identity per a total 188 bp sequence of the PCR products to the *S_C3_* original sequence of Slater’s Crimson China (No.613).

*Wild ancestral origin of S_C4_ (Rosa chinensis)*

**Table S11-4** shows the list of wild species and old cultivars with positive PCR amplifications of *S_C4_*-specific primers. *Rosa gigantea* (pink flower type) has *S_C4_* *S-RNase* identical to those of old CF cultivar *R. chinensis* and once-flowering cultivar *R. chinensis* ‘Major’.

**Table S11-4.** Wild species and old cultivars with positive PCR amplifications of *S_C4_*-specific primers. Data were extracted from Supplementary data **Table D6**.


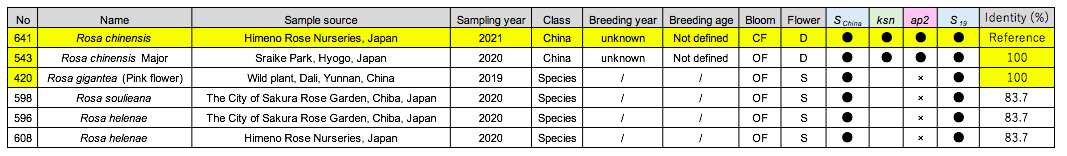


Identity (%) indicates nucleotide sequence identity per a total 361 bp sequence of the PCR products (except for primer regions) to the *S_C4_* original sequence of *Rosa chinensis* (No.641).

*Wild ancestral origin of S_C5_ (Mutabilis)*

**Figure S11-3** shows the alignment of *S_C5_*-like *S-RNase* partial sequence of *R. rubus* with the *S_C5_ S-RNase* reference sequence of *R. chinensis* ‘Mutabilis’. There are four SNPs, and we did not find any other wild species with positive PCR amplification with the *S_C5_* specific primers (**Table D6**).


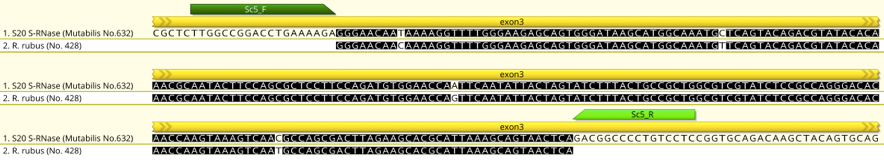


**Figure S11-3.** Alignment of *S_C5_*-like *S-RNase* partial sequence of *R. rubus* with the *S_C5_ S-RNase* reference sequence of *R. chinensis* ‘Mutabilis’.
