# Supplementary material for: The identification of the Rosa S-locus provides new insights into the breeding and wild origins of continuous-flowering roses
Source: Hortic Res. 2022 Oct 1;9:uhac155. doi: 10.1093/hr/uhac155 (PMC9527601; doi:10.1093/hr/uhac155)
Supplement: Web_Material_uhac155 [file web_material_uhac155.zip › Supplementary Information 2.docx]

**Supplementary information 2**

**The identification of the *Rosa* *S*-locus provides new insights into the breeding and wild origins of continuous-flowering roses**

Koji Kawamura^1*^, Yoshihiro Ueda^2,3^, Shogo Matsumoto^4^, Takanori Horibe^4,5^, Shungo Otagaki^4^, Li Wang^6^, Guoliang Wang^7,8^, Laurence Hibrad-Saint Oyant^9^, Fabrice Foucher^9^, Marcus Linde^10^, Thomas Debener^10^

^1^, Department of Environmental Engineering, Osaka Institute of Technology, Japan

^2^, Gifu International Academy of Horticulture, Japan

^3^, Gifu World Rose Garden, Japan

^4^, Graduate School of Bioagricultural Sciences, Nagoya University, Japan

^5^, College of Bioscience and Biotechnology, Chubu University, Japan

^6^, College of Life Sciences, Sichuan University, China

^7^, Jiangsu Provincial Department of Agriculture and Rural Affairs, China

^8^, Agricultural University of Nanjing, China.

^9^, Univ Angers, INRAE, Institut Agro, IRHS, SFR QUASAV, F-49000 Angers, France

^10^, Leibniz Universität, Hannover, Germany

^*^Corresponding author: Koji Kawamura

E-mail: [koji.kawamura@oit.ac.jp](mailto:koji.kawamura@oit.ac.jp)

Tel: +81-(0)6-4300-6848

Affiliation: Department of Environmental Engineering, Osaka Institute of Technology

Address: 5-16-1 Ohmiya, Asahi-ku, Osaka, 535-8585 JAPAN

**Spatiotemporal expression patterns of *S-RNase* in the rose**

*The expression-levels of S-RNase in the pistil of Old Blush and R. multiflora were high at immature floral bud stage 4-6 days before anthesis and decreased in the balloon stage 1-day before anthesis.*

**Materials & Methods**

Three to 20 floral buds at different developmental stages (immature, balloon) were sampled from each of the four individuals of *R.chinensis* ‘Old Blush’ and *R. multiflora*. Petals, pistils, and stamens were separated, and their total RNA was extracted. Three young leaves were also sampled for each individual. cDNA synthesis was performed with the PrimeScript RT reagent Kit with genomic DNA Eraser (TaKaRa) according to the manufacturer’s protocols. A real-time PCR assay was performed using PowerUp SYBR Green Master Mix and a StepOne instrument (Life Technologies). A 10-μL mix for each PCR run was prepared as follows: 3 μL water, 0.5 μL of each primer (0.5 μM), 1 μL cDNA template, and 5 μL Fast SYBR Green Master Mix. The reactions were performed using a Fast cycling mode: (1) 50°C, 120 s; (2) 95°C, 120 s; (3) 95°C, 3 s; (4) 60°C, 30 s; (5) Back to (3) 39 times, (6) 95°C, 15 s; (7) 60°C, 60 s; (8) 95°C, 15 s. Average Ct values of two or three repetitions were obtained and used for analyses. *See* Supplementary data **Table D1** for the information on the primers used.

**Results & Discussion**

The expression levels of *S-RNase* in the pistil of *R. multiflora* and *R. chinensis* ‘Old Blush’ were higher in immature buds 4-6 days before anthesis than in buds at the balloon stage, just before anthesis. This suggests that the appropriate materials for identifying *S-RNase* in the rose are not the floral buds just before anthesis (balloon stage), but the immature stages a few days before anthesis. The accumulation of S-RNase proteins in the pistil may lead to a negative feedback on the expression of the *S-RNase*. A similar expression pattern of *S-RNase* in the pistil was observed in *Citrus* (Liang *et al*., 2020).

**
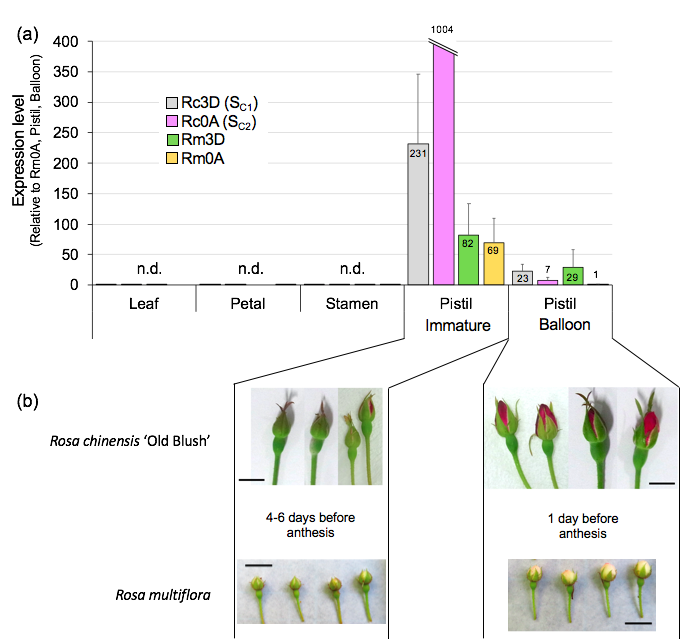
**

**Figure S2-1.** Expression levels of candidate *S-RNase* genes in different organs and developmental stages. **(a)** A housekeeping gene, TCTP was used as an internal control, and standardized expression levels of *S-RNase* are shown as a relative scale (to that of Rm0A in the pistil at balloon stage). Numbers above or in the bar indicate averages of the relative expression levels of four individuals, and error bars indicate standard error. **(b)** Picture of floral buds at different developmental stages. Bars show 1cm scale. At the balloon stage, the floral buds are swollen, and petals are visible.

**References**

Liang, M. *et al.* Evolution of self-compatibility by a mutant *S_m_-RNase* in citrus. *Nat.Plants* **6**, 131–142 (2020).
