# Supplementary material for: The identification of the Rosa S-locus provides new insights into the breeding and wild origins of continuous-flowering roses
Source: Hortic Res. 2022 Oct 1;9:uhac155. doi: 10.1093/hr/uhac155 (PMC9527601; doi:10.1093/hr/uhac155)
Supplement: Web_Material_uhac155 [file web_material_uhac155.zip › Supplementary Information 3.docx]

**Supplementary information 3**

**The identification of the *Rosa* *S*-locus provides new insights into the breeding and wild origins of continuous-flowering roses**

Koji Kawamura^1*^, Yoshihiro Ueda^2,3^, Shogo Matsumoto^4^, Takanori Horibe^4,5^, Shungo Otagaki^4^, Li Wang^6^, Guoliang Wang^7,8^, Laurence Hibrad-Saint Oyant^9^, Fabrice Foucher^9^, Marcus Linde^10^, Thomas Debener^10^

^1^, Department of Environmental Engineering, Osaka Institute of Technology, Japan

^2^, Gifu International Academy of Horticulture, Japan

^3^, Gifu World Rose Garden, Japan

^4^, Graduate School of Bioagricultural Sciences, Nagoya University, Japan

^5^, College of Bioscience and Biotechnology, Chubu University, Japan

^6^, College of Life Sciences, Sichuan University, China

^7^, Jiangsu Provincial Department of Agriculture and Rural Affairs, China

^8^, Agricultural University of Nanjing, China.

^9^, Univ Angers, INRAE, Institut Agro, IRHS, SFR QUASAV, F-49000 Angers, France

^10^, Leibniz Universität, Hannover, Germany

^*^Corresponding author: Koji Kawamura

E-mail: [koji.kawamura@oit.ac.jp](mailto:koji.kawamura@oit.ac.jp)

Tel: +81-(0)6-4300-6848

Affiliation: Department of Environmental Engineering, Osaka Institute of Technology

Address: 5-16-1 Ohmiya, Asahi-ku, Osaka, 535-8585 JAPAN

**Alignments and conserved sites of the S-RNase protein in the rose**

*Five conserved sites of the S-RNase proteins isolated from Old Blush, R. multiflora, and R. rugosa are shown.*

We identified 21 *S-RNase* alleles from (i) genome databases of *R. chinensis*, *R. multiflora*, and *R. rugosa*, (ii) our original RNA-seq data (**Table D3**), and (iii) RT-PCR and sequencing (**Table S3-1**). They were classified either into the clade of 3D S-RNase (3D) or 0A S-RNase (0A) based on the molecular phylogenetic tree of S-RNase proteins (**Fig.1c**).

**Table S3-1.** *S-RNase* alleles identified in this study.

| *S*-allele | Clade^1^ | Identification from^2^ |
| --- | --- | --- |
| *S_C1_* | 3D | Genome database (Raymond *et al*. 2018), RNA-seq of OB and Rm1-3 (**Table D3**) |
| *S_C2_* | 0A | Genome database (Hibrand Saint-Oyant *et al*. 2018), RNA-seq of OB (**Table D3**) |
| *S_C3_* | 0A | RT-PCR of Slater's Crimson China (**Fig. S10-1**) (parcial sequence) |
| *S_C4_* | 0A | RT-PCR of *Rosa chinensis* (**Fig. S10-1**) (partial sequence) |
| *S_C5_* | 0A | RT-PCR of *Rosa chinensis* 'Mutabilis' (**Fig. S10-1**) |
| *S_6_* | 0A | RNA-seq of Rm08, Rm13 (**Table D3**) |
| *S_7_* | 3D | RNA-seq of Rm08, Rm09 (**Table D3**) |
| *S_8_* | 0A | RNA-seq of Rm09 (**Table D3**) |
| *S_9_* | 3D | RNA-seq of Rm27, Rm33 (**Table D3**) |
| *S_10_* | 0A | RNA-seq of Rm28 (**Table D3**) |
| *S_11_* | 3D | RNA-seq of Rm27 (**Table D3**) |
| *S_12_* | 0A | RNA-seq of Rm33 (**Table D3**) |
| *S_13_* | 3D | RNA-seq of Rm28 (**Table D3**) |
| *S_14_* | 3D | RNA-seq of Rm1 (**Table D3**) |
| *S_15_* | 3D | Genome database (Nakamura *et al*. 2018), RNA-seq of Rm2, Rm3 (**Table D3**) |
| *S_16_* | 3D | Genome database (Nakamura *et al*. 2018) |
| *S_17_* | 3D | CAPS marker characterization of Rm24 (unpublished) |
| *S_18_* | 3D | Genome database (Chen *et al*. 2021), RNA-seq of Rg46 (**Table D3**) |
| *S_19_* | 0A | RNA-seq of Rg46 (**Table D3**) |
| *S_20_* | 3D | Genome database (Zang *et al*. 2021) |
| *S_21_* | 0A | RT-PCR of The Fairy (TF) (**Fig. S4-1**) |

^1,^ Molecular phylogenetic positions of S-RNase protein (**Fig.1c**).

^2,^ OB = Old Blush; Rm = *Rosa multiflora*; Rg = *Rosa rugosa*.

**Table S3-2**. Summary of *S*-genotypes of the roses used for the identification of *S-RNase* alleles.

| Species or cultivar name | Plant ID | S-genotype^1^ |
| --- | --- | --- |
| *Rosa chinensis* 'Old Blush' | OB | S_C1_/S_C2_ |
| *Rosa multiflora* | Genome database (Nakamura *et al*. 2018) | S_15_/S_16_ |
| *Rosa multiflora* | Rm1 | S_C1_/S_14_ |
| *Rosa multiflora* | Rm2 | S_C1_/S_15_ |
| *Rosa multiflora* | Rm3 | S_C1_/S_15_ |
| *Rosa multiflora* | Rm4 | S_C1_/S_12_ |
| *Rosa multiflora* | Rm08 | S_6_/S_7_ |
| *Rosa multiflora* | Rm09 | S_7_/S_8_ |
| *Rosa multiflora* | Rm13 | S_6_/S_11_ |
| *Rosa multiflora* | Rm24 | S_9_/S_17_ |
| *Rosa multiflora* | Rm27 | S_9_/S_11_ |
| *Rosa multiflora* | Rm28 | S_10_/S_13_ |
| *Rosa multiflora* | Rm32 | S_12_/S_13_ |
| *Rosa multiflora* | Rm33 | S_9_/S_12_ |
| *Rosa multiflora* | Rm50 | S_9_/S_10_ |
| *Rosa rugosa* | Genome database, GD1 (Chen *et al*. 2021) | S_18_/S_x_ |
| *Rosa rugosa* | Genome database, GD2 (Zang *et al*. 2021) | S_20_/S_x_ |
| *Rosa rugosa* | Rg46 | S_18_/S_19_ |

^1^, S_x_ = Unknown

**
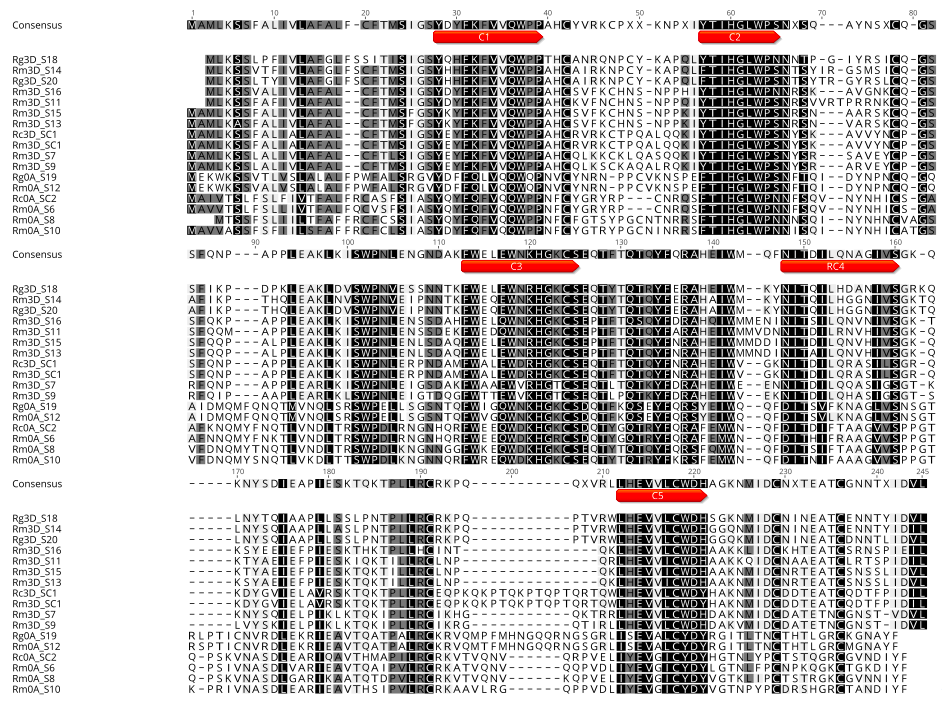
**

**Figure S3-1**. An alignment of the S-RNase protein in the rose. Conserved sites in Rosaceae S-RNase (Ushijima *et al*., 1998) are shown below the consensus sequences. Sequence data are available from Supplementary data **Table D4**.

**
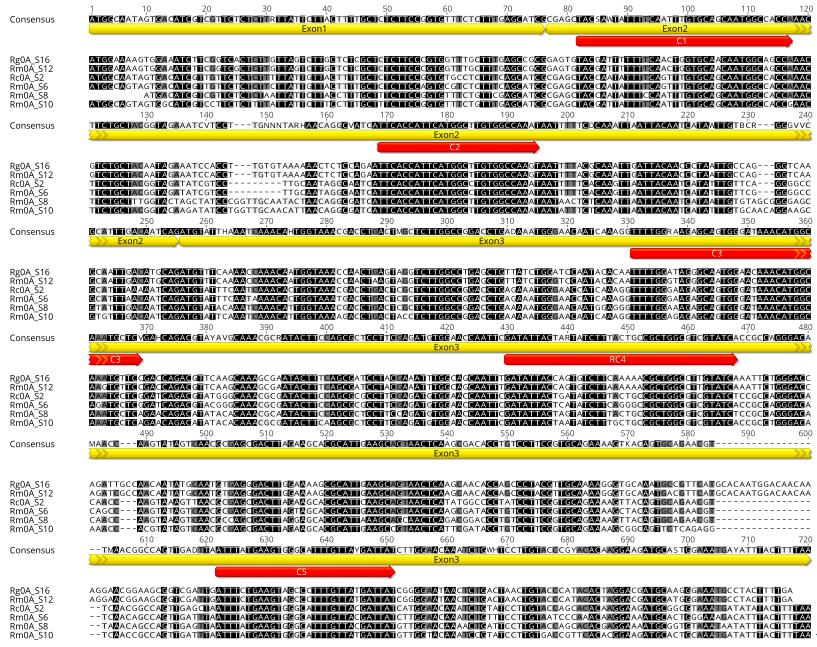
**

**Figure S3-2.** Alignment of 0A *S-RNase* cDNA sequences. Sequence data are available from Supplementary data **Table D4**.

**Figure S3-3.** Alignment of 3D *S-RNase* cDNA sequences. Sequence data are available from Supplementary data **Table D4**.

**References**

Chen, F. *et al.* A chromosome-level genome assembly of rugged rose (*Rosa rugosa*) provides insights into its evolution, ecology, and floral characteristics. *Hortic Res* **8,**141 (2021).

Nakamura N. *et al*. Genome structure of *Rosa multiflora*, a wild ancestor of cultivated roses. *DNA Res*. **25**: 113-121 (2018).

Hibrand Saint-Oyant L *et al*. A high-quality genome sequence of Rosa chinensis to elucidate ornamental traits. *Nature Plants* **4**: 473-484 (2018).

Raymond O. *et al*. The Rosa genome provides new insights into the domestication of modern roses. *Nature Genet.* **50**: 772-777 (2018).

Ushijima, K. *et al.* Cloning and characterization of cDNAs encoding S-RNases from almond (*Prunus dulcis*): primary structural features and sequence diversity of the S-RNases in Rosaceae. *Mol. General Genet.* **260**, 261-268 (1998).

Zang, F. *et al*. A high-quality chromosome-level genome of wild *Rosa rugosa*, *DNA Res.* **28**: dsab017 (2021).
