# Supplementary material for: The identification of the Rosa S-locus provides new insights into the breeding and wild origins of continuous-flowering roses
Source: Hortic Res. 2022 Oct 1;9:uhac155. doi: 10.1093/hr/uhac155 (PMC9527601; doi:10.1093/hr/uhac155)
Supplement: Web_Material_uhac155 [file web_material_uhac155.zip › Supplementary Information 4.docx]

**Supplementary information 4**

**The identification of the *Rosa* *S*-locus provides new insights into the breeding and wild origins of continuous-flowering roses**

Koji Kawamura^1*^, Yoshihiro Ueda^2,3^, Shogo Matsumoto^4^, Takanori Horibe^4,5^, Shungo Otagaki^4^, Li Wang^6^, Guoliang Wang^7,8^, Laurence Hibrad-Saint Oyant^9^, Fabrice Foucher^9^, Marcus Linde^10^, Thomas Debener^10^

^1^, Department of Environmental Engineering, Osaka Institute of Technology, Japan

^2^, Gifu International Academy of Horticulture, Japan

^3^, Gifu World Rose Garden, Japan

^4^, Graduate School of Bioagricultural Sciences, Nagoya University, Japan

^5^, College of Bioscience and Biotechnology, Chubu University, Japan

^6^, College of Life Sciences, Sichuan University, China

^7^, Jiangsu Provincial Department of Agriculture and Rural Affairs, China

^8^, Agricultural University of Nanjing, China.

^9^, Univ Angers, INRAE, Institut Agro, IRHS, SFR QUASAV, F-49000 Angers, France

^10^, Leibniz Universität, Hannover, Germany

^*^Corresponding author: Koji Kawamura

E-mail: [koji.kawamura@oit.ac.jp](mailto:koji.kawamura@oit.ac.jp)

Tel: +81-(0)6-4300-6848

Affiliation: Department of Environmental Engineering, Osaka Institute of Technology

Address: 5-16-1 Ohmiya, Asahi-ku, Osaka, 535-8585 JAPAN

**Genetic mapping of *S-RNase* in chromosome zero by using an *F_1_* diploid rose population**

*The F_1_-hybrid population FW was used for genetic mapping of the Rc0A (S_C2_) S-RNase of Old Blush identified in chromosome 0. Methods of S-genotyping the parental roses and development of three additional markers linked to the S gene are described.*

**Materials & Methods**

The mapping population FW (Kawamura *et al.*, 2011), which consists of 97 *F_1_* hybrids derived from the female parent TF (The Fairy) and the male parent RW (*R. wichuraiana*), was used for genetic mapping of the newly-identified *S-RNase* of Old Blush.

*Methods for mapping the candidate S-RNase gene in TF*

Partial sequences of *S-RNase* were amplified from the gDNA of TF by using universal primers designed on the conserved sites of *S_C2_*-like *S-RNase* exon3 (S2_e3_F1, S2_e3_R1; **Table D1**). Direct sequencing of the PCR products suggested that TF has *S_C2_* and a *S_C2_*-like new allele (named *S_21_*). Whole CDSs were subsequently determined by RT-PCR of the mRNA extracted from the pistil of TF, and confirmed that the TF has the identical *S_C2_* *S-RNase* to Old Blush (**Fig. S4-1**).

The *S_C2_*/*S_21_* *S-RNase* of TF was mapped by the High Resolution Melting (HRM) method. A real-time PCR assay was performed using PowerUp SYBR Green Master Mix with primers S2L_F and S2L_R (**Table D1**) and a StepOne instrument (Life Technologies). A 10-μL mix for each PCR run was prepared as follows: 3 μL water, 0.5 μL of each primer (0.5 μM), 1 μL DNA template, and 5 μL Fast SYBR Green Master Mix. The reactions were performed using a Fast cycling mode: (1) 50°C, 120 s; (2) 95°C, 120 s; (3) 95°C, 3 s; (4) 60°C, 30 s; (5) Back to (3) 39 times; (6) 95°C, 15 s; (7) 60°C, 60 s; (8) 95°C, 15 s. Steps (6) to (8) are for HRM analysis. A derivative melt curve plot of PCR products determines the temperature of melt peak (T_m_). The HRM analysis of the FW population classifies *F_1_* hybrids either into the class of T_m_ = 83.4 corresponding to *S_C2_*, or into the class of T_m_ = 82.3 corresponding to *S_21_*. The T_m_ of TF (*S_C2_*/*S_21_*) is the intermediate (T_m_ = 82.9) between the two classes. Based on the HRM genotyping, the *S_C2_*/*S_21_* locus was mapped on the TF3 map.

To assess the accuracy of the HRM genotyping, a CAPS marker was also developed. A PCR was performed using EmeraldAmp PCR Master Mix (TaKaRa), the primers S2L_F and S2L_R, and a DNA template. Thermal cycling was as follows: (1) 2 min of 95℃; (2) 30 s of 95℃; (3) 30 s of 61℃; (4) 20 s of 72℃; and (5) go back to step (2) 39 times. 5μL of the PCR product was digested with 0.7 μL HaeIII (BioLabs, 50 U/μL). The genotyping result of CAPS perfectly matched the HRM (**Fig.S4-2**).

**
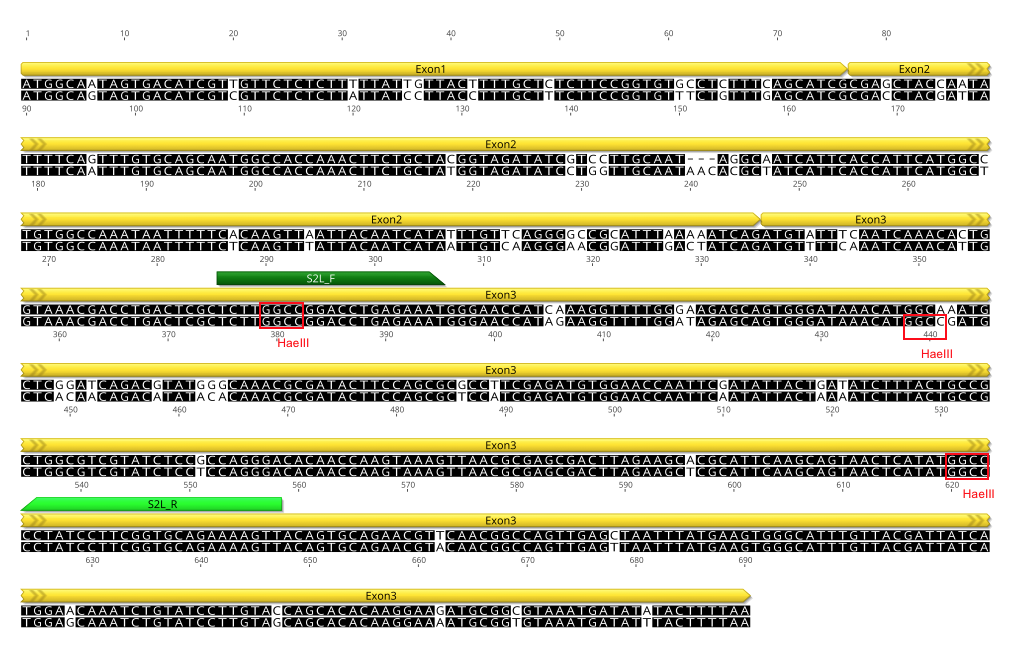
**

**Figure S4-1**. Alignment of the CDS of *S_C2_* and *S_21_* *S-RNase* of TF. The upper sequence is *S_C2_* (=Rc0A) and the lower one is *S_21_*. The restriction sites of HaeIII in the PCR products of primers S2L_F and S2L_R are also indicated.


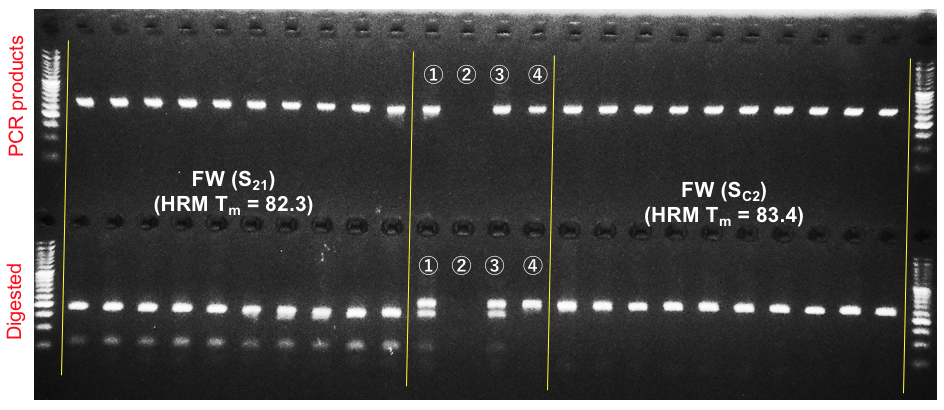


**Figure S4-2**. CAPS maker analysis for genotyping *S_C2_* and *S_21_ S-RNase* in the FW mapping population. The upper line shows undigested PCR products, and the lower line shows the results of digested samples with HaeIII. Two groups of *F_1_* hybrids with different T_m_ by HRM analysis show consistently different CAPS results, demonstrating the accuracy of the HRM analysis. Lane ①, ③ = TF, ② = RW (null allele), ④ = OB (*S_C2_*)

*Method for mapping the candidate S-RNase gene in RW*

*S_C1_*-like *S-RNase* was identified from the genome sequence of RW (SRR6175520) and named *S_1w_* (**Fig.S4-3**). Specific primers (**Table D1**) were designed on the exon3, and PCR amplification of *S_1w_* was performed using EmeraldAmp PCR Master Mix (TaKaRa) with thermal cycling: (1) 2 min of 95°C; (2) 3 0s of 95°C; (3) 30 s of 60°C; (4) 20 s of 72°C; and (5) go back to step (2) 34 times. The PCR products were visualized in a 2% Agarose gel.


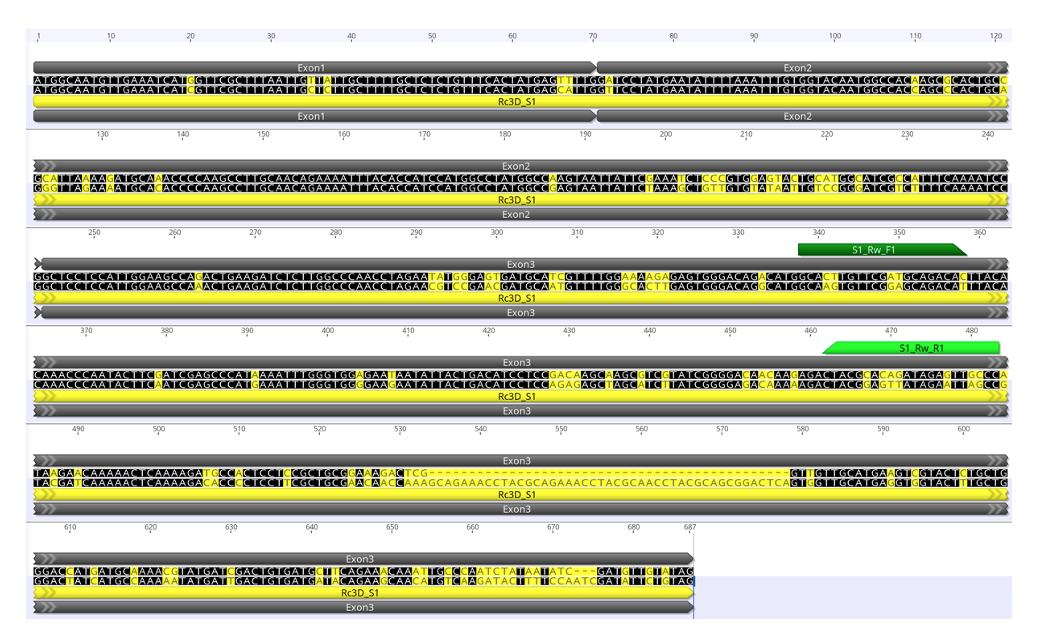


**Figure S4-3**. Alignment of the CDS of *S_1w_* and *S_C1_ S-RNase*. The upper sequence is *S_1w_* and the lower one is *S_C1_* (=Rc3D). The specific primers (S1_Rw_F1, S1_Rw_R1) were designed on the sequence of *S_1w_*.

*Methods for mapping the other genomic markers RoKN1, Ro227, and Ro224*

Three additional markers surrounding the *S*-*RNase* were added to the previous map to estimate genomic position of the *S-RNase*. *RoKN1* is a Class I Knotted1-like homeobox gene (RC3G0335800, Chr3g0457121), analyzed as a candidate gene controlling thorn formation in the rose (Matsumoto unpublished data). *Ro227* and *Ro224* are markers closely linked to the 3C_1_ (Chr3g0457471) and 3C_2_ (Chr3g0457441*) S-RNase*, respectively.


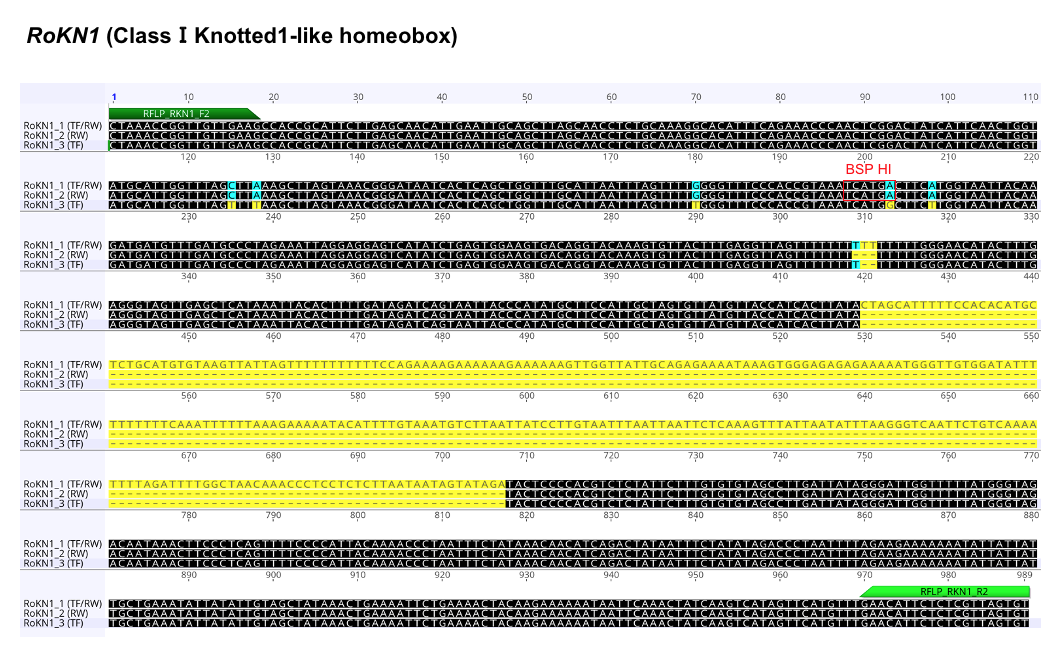


**Figure S4-4**. Alignment of partial sequences of three alleles of *RoKN1* in TF and RW. The partial sequences of *RoKN1* were amplified by the primers RFLP_RKN_F2 and RFLP_RKN_R2. The allele RoKN1_1 was scored by the presence of a 288 bp insertion, and the alleles RoKN1_2 and RoKN1_3 were distinguished by the restriction fragment length polymorphism with the enzyme BSP HI.


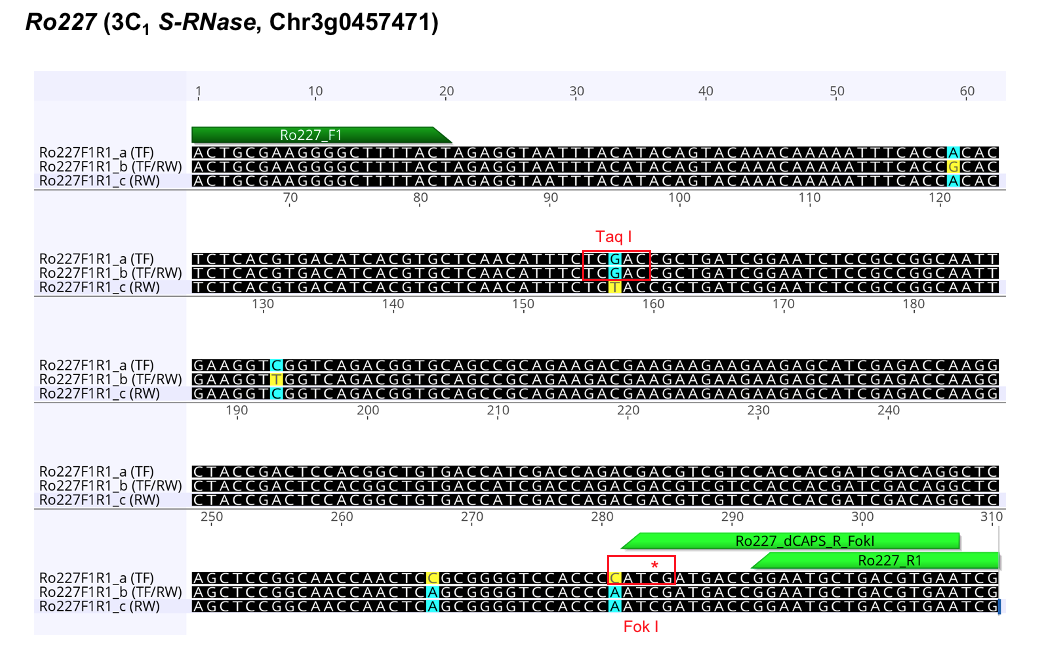


**Figure S4-5**. Alignment of partial sequences of three alleles of *Ro227* in TF and RW. dCAPS marker with Fok I, and CAPS marker with Taq I, were developed to score the presence of allele ***a*** and allele ***c***, respectively.


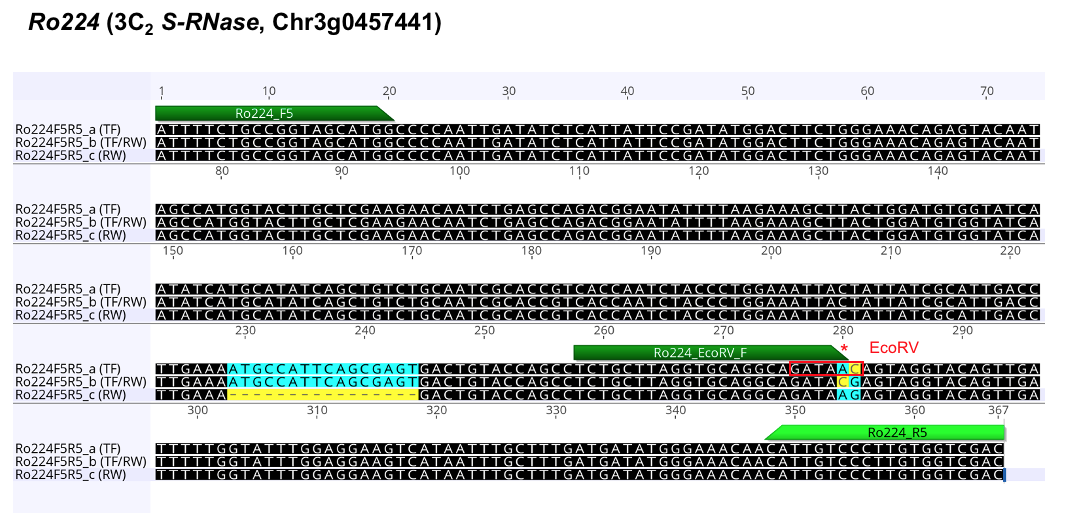


**Figure S4-6**. Alignment of partial sequences of three alleles of *Ro224* in TF and RW. dCAPS marker with EcoRV was developed to score the presence of allele ***a***.

**References**

Kawamura, K. *et al.* Quantitative trait loci for flowering time and inflorescence architecture in rose. *Theor. Appl. Genet.* **122**, 661-75 (2011).
