# Supplementary material for: The identification of the Rosa S-locus provides new insights into the breeding and wild origins of continuous-flowering roses
Source: Hortic Res. 2022 Oct 1;9:uhac155. doi: 10.1093/hr/uhac155 (PMC9527601; doi:10.1093/hr/uhac155)
Supplement: Web_Material_uhac155 [file web_material_uhac155.zip › Supplementary Information 5.docx]

**Supplementary information 5**

**The identification of the *Rosa* *S*-locus provides new insights into the breeding and wild origins of continuous-flowering roses**

Koji Kawamura^1*^, Yoshihiro Ueda^2,3^, Shogo Matsumoto^4^, Takanori Horibe^4,5^, Shungo Otagaki^4^, Li Wang^6^, Guoliang Wang^7,8^, Laurence Hibrad-Saint Oyant^9^, Fabrice Foucher^9^, Marcus Linde^10^, Thomas Debener^10^

^1^, Department of Environmental Engineering, Osaka Institute of Technology, Japan

^2^, Gifu International Academy of Horticulture, Japan

^3^, Gifu World Rose Garden, Japan

^4^, Graduate School of Bioagricultural Sciences, Nagoya University, Japan

^5^, College of Bioscience and Biotechnology, Chubu University, Japan

^6^, College of Life Sciences, Sichuan University, China

^7^, Jiangsu Provincial Department of Agriculture and Rural Affairs, China

^8^, Agricultural University of Nanjing, China.

^9^, Univ Angers, INRAE, Institut Agro, IRHS, SFR QUASAV, F-49000 Angers, France

^10^, Leibniz Universität, Hannover, Germany

^*^Corresponding author: Koji Kawamura

E-mail: [koji.kawamura@oit.ac.jp](mailto:koji.kawamura@oit.ac.jp)

Tel: +81-(0)6-4300-6848

Affiliation: Department of Environmental Engineering, Osaka Institute of Technology

Address: 5-16-1 Ohmiya, Asahi-ku, Osaka, 535-8585 JAPAN

**Validation of the *S-RNase* based *S*-genotyping by pollination experiments**

*Protocol for S-genotyping of seed and the outline of the pollination experiments using 20 pairs of diploid roses sharing one S-allele (i.e., half-compatible) are described.*

***Materials & Methods***

Old Blush (*S_C1_*/*S_C2_*), The Fairy (*S_C2_*/*S_21_*), *R. chinensis* ‘Single white-eye’ (*S_C1_*/*S_12_*), and 11 wild individuals of *R. multiflora* were selected based on their *S*-genotypes. Pairs of pollination are described in **Figure S5-2**. Before anthesis, flower buds were bagged to prevent open pollination. Petals and anthers were removed at the balloon stage, and outcross pollen grains were put on the exposed stigma. Pollinations were carried out from April to May in 2018 and 2020, and matured fruits were collected from September to October of the same years. The fruits were opened in the laboratory, and the achenes (seeds) were collected (**Fig. S5-1**). The protocol of DNA extraction from seed, and PCR method of *S*-genotyping were performed as follows.


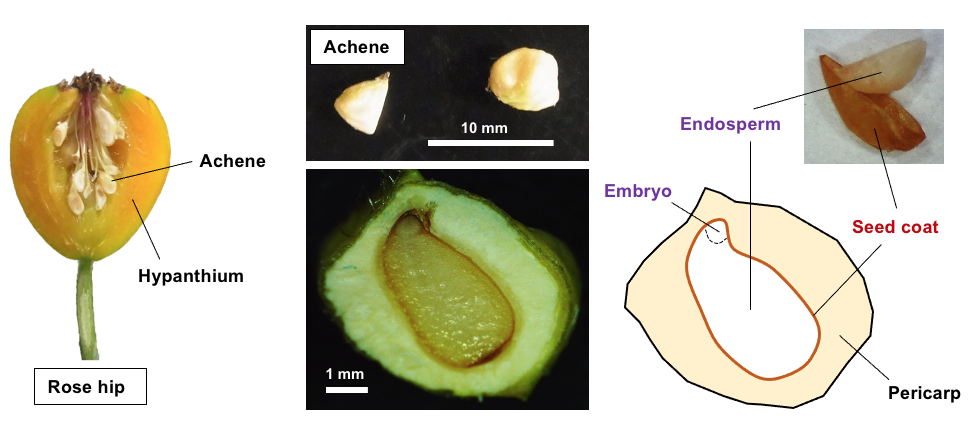


**Figure S5-1.** Structure of rose hip and seed.

*Protocol of DNA extraction from seed*

1. Pericarp of the seed (achene ) was incised by a razor, and the endosperm of the seed was carefully extracted　(**note**: seed coat must be removed, as it is a mother tissue).
2. Endosperm was frozen with liquid nitrogen and crushed by beads-beating equipment.
3. 300μL CTAB buffer was added and heated to 65℃ for 10 min.
4. Centrifuge at 11,000 ×g for 1 min at room temperature; transfer the supernatant to a new tube.
5. Add 250μL TE-saturated phenol, shake 3 min, and freeze at −80℃, >30 min.
6. Melt the frozen sample at room temperature and centrifuge at 20,000 ×g for 5 min at room temperature.
7. Remove the layer of phenol (yellow), add 125μL TE-saturated phenol and 125μL CIA, and mix well.
8. Centrifuge at 20,000 ×g for 5 min at room temperature.
9. Transfer the layer of water (colorless) to a new tube, and purify by ethanol precipitation.

*Protocol of S-genotyping by PCR*

The DNA solution was diluted to be approximately 0.25 ng μL^-1^ to prepare the PCR solution using EmeraldAmp PCR Master mix (TaKaRa), and PCR was performed with the following thermal cycle. *See* **Table D1** for primer information.


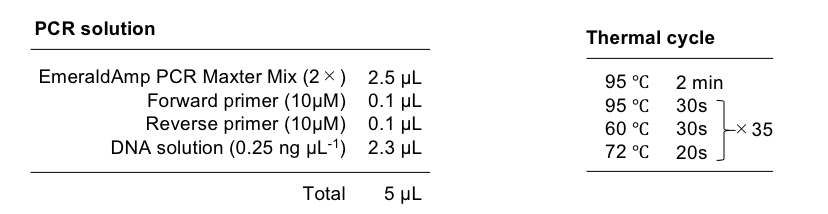


**
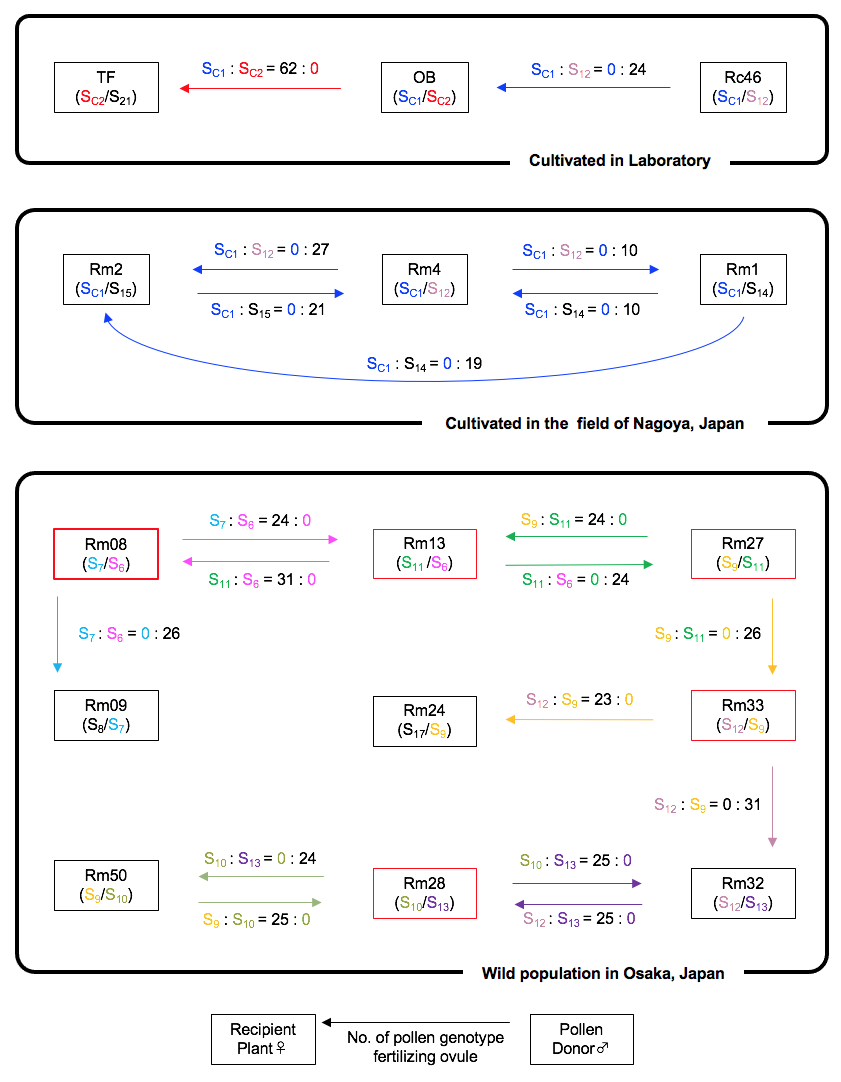
**

**Figure S5-2.** Summary of pollination experiments and roses used for the experiments. TF = The Fairy, OB = Old Blush, Rc46 = *Rosa chinensis* ‘Single white-eye’ No.46, and Rm = *Rosa multiflora*. The *S*-genotype is shown in parentheses. Numbers of pollen *S*-genotypes fertilizing ovules are shown above or below the arrows. The arrows indicate the direction of pollination.

**Results**

**Table S5-1** shows the results of *S*-genotyping of seeds produced by half-compatible pairs of roses. A total of 469 seeds derived from 20 half-compatible pairs of roses were analyzed. For the test of *S_C1_ S-RNase*-based *S*-genotyping, five roses sharing the *S_C1_* were used for the pollination experiment: Old Blush (*S_C1_*/*S_C2_*), *R. chinensis* Rc46 (*S_C1_*/*S_12_*), *R. multiflora* Rm1 (*S_C1_*/*S_14_*), Rm2 (*S_C1_*/*S_15_*), and Rm4 (*S_C1_*/*S_12_*). A total 111 seeds were analyzed, and the results showed that no S_C1_ pollen fertilized the ovule. The pollination tests were also performed for *S_C1_*-like *S-RNase* (*S_7_*, *S_9_*, *S_11_*, *S_13_*) identified in *R. multiflora* by using 6 plants: Rm13, Rm24, Rm27, Rm28, Rm32, Rm33 (*See* also **Fig.S5-2** for their *S*-genotypes). A total 174 seeds (=26 + 50 + 48 + 50) were analyzed, and all results are well agreement with the *S-RNase* based *S*-genotyping. The same pollination tests were conducted for the newly-identified *S_C2_* allele of Old Blush. TF (*S_C2_*/*S_21_*) was pollinated by pollen of Old Blush (*S_C1_*/*S_C2_*), and *S*-genotypes of 60 seeds were determined. The result shows that no *S_C2_* pollen fertilized the ovule. The pollination tests for the *S_C2_*-like *S-RNase* (*S_6_*, *S_10_*, *S_12_*) identified in *R. multiflora* resulted in the same consistent results of *S*-genotyping of total 124 seeds (= 49 + 47 + 28).

Additional results of *S*-genotyping for a *F_1_* hybrid population, 94/1 (Debener & Mattiesch, 1999) and some full-compatible pairs are also added in **Table S5-1**. The S-genotyping results of the 94/1 population are shown in **Fig.S5-3**.

**Table S5-1**. Frequency of *S*-genotypes in seeds (or *F_1_* hybrids) produced by hand pollinations. Each row shows different pairs of roses.


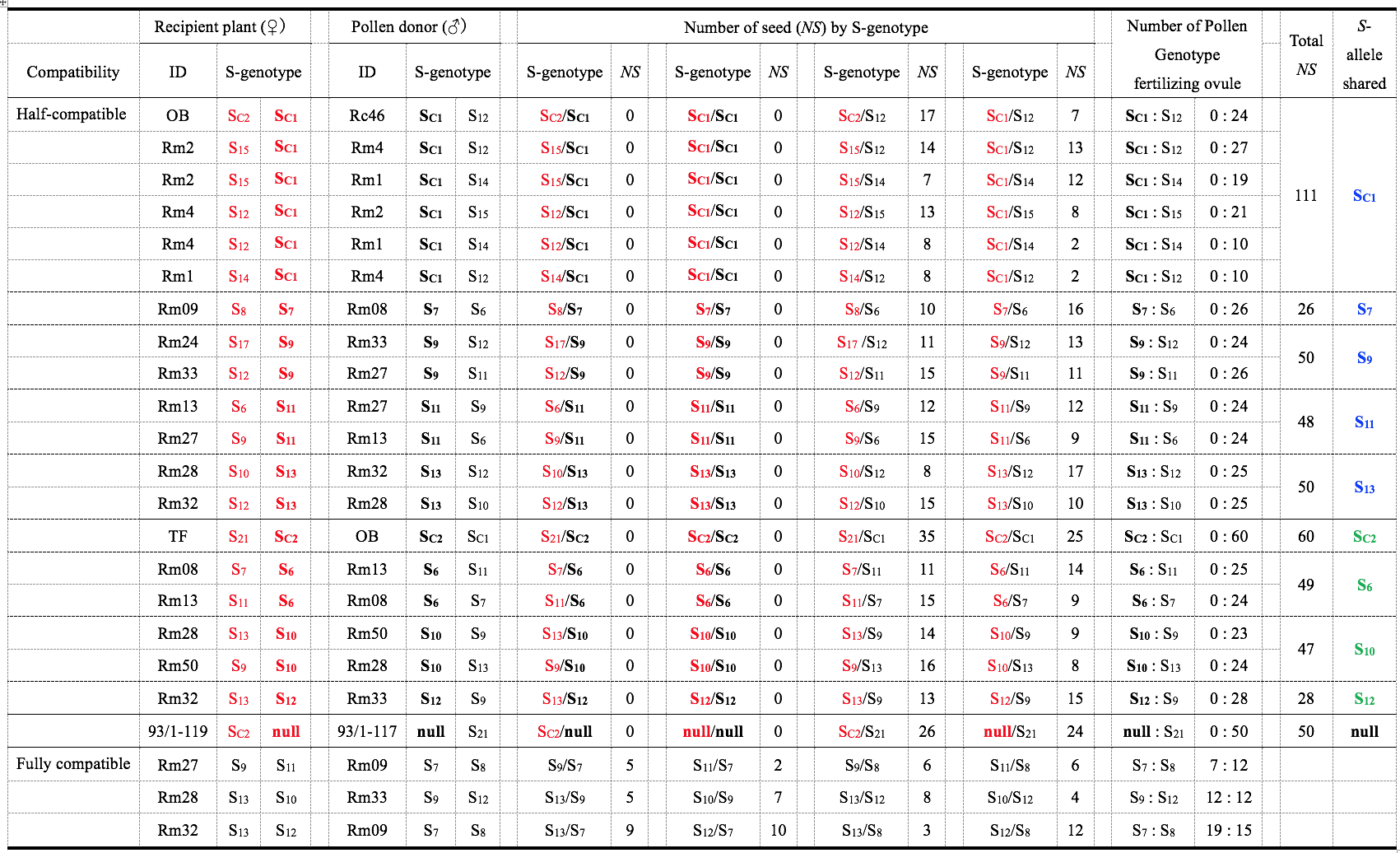


**
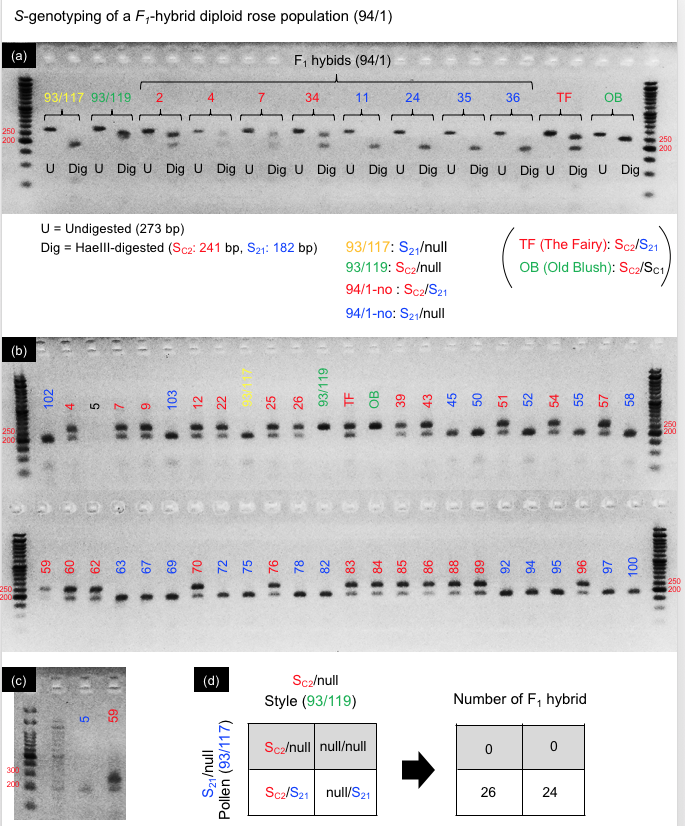
**

**Figure S5-3.** Agarose gel images for determining *S*-genotypes of 94/1 *F_1_* hybrids. 94/1 hybrids are derived from the cross between 93/117 and 93/119 (Debener & Mattiesch, 1999) **(a)** Test of the CAPS marker. **(b)-(c)** *S*-genotyping of 94/1 hybrids by the CAPS. **(d)** A hypothesis: null allele may be the same allele between the parents, and the pollen grains of the null allele were rejected by the style.

**References**

Debener, T., & Mattiesch, L. Construction of a genetic linkage map for roses using RAPD and AFLP markers. *Theor. Appl. Genet.* **99**, 891-899 (1999).
