# Supplementary material for: The identification of the Rosa S-locus provides new insights into the breeding and wild origins of continuous-flowering roses
Source: Hortic Res. 2022 Oct 1;9:uhac155. doi: 10.1093/hr/uhac155 (PMC9527601; doi:10.1093/hr/uhac155)
Supplement: Web_Material_uhac155 [file web_material_uhac155.zip › Supplementary Information 6.docx]

**Supplementary information 6**

**The identification of the *Rosa* *S*-locus provides new insights into the breeding and wild origins of continuous-flowering roses**

Koji Kawamura^1*^, Yoshihiro Ueda^2,3^, Shogo Matsumoto^4^, Takanori Horibe^4,5^, Shungo Otagaki^4^, Li Wang^6^, Guoliang Wang^7,8^, Laurence Hibrad-Saint Oyant^9^, Fabrice Foucher^9^, Marcus Linde^10^, Thomas Debener^10^

^1^, Department of Environmental Engineering, Osaka Institute of Technology, Japan

^2^, Gifu International Academy of Horticulture, Japan

^3^, Gifu World Rose Garden, Japan

^4^, Graduate School of Bioagricultural Sciences, Nagoya University, Japan

^5^, College of Bioscience and Biotechnology, Chubu University, Japan

^6^, College of Life Sciences, Sichuan University, China

^7^, Jiangsu Provincial Department of Agriculture and Rural Affairs, China

^8^, Agricultural University of Nanjing, China.

^9^, Univ Angers, INRAE, Institut Agro, IRHS, SFR QUASAV, F-49000 Angers, France

^10^, Leibniz Universität, Hannover, Germany

^*^Corresponding author: Koji Kawamura

E-mail: [koji.kawamura@oit.ac.jp](mailto:koji.kawamura@oit.ac.jp)

Tel: +81-(0)6-4300-6848

Affiliation: Department of Environmental Engineering, Osaka Institute of Technology

Address: 5-16-1 Ohmiya, Asahi-ku, Osaka, 535-8585 JAPAN

***Colchicine-induced chromosome doubling resulted in self-compatible tetraploid***

*Data showing that polyploidization breaks down the SI of diploid rose is described.*

Pollination experiments were performed to test the hypothesis that polyploidization breaks down the SI of diploid rose. A tetraploid genotype (CT40) was obtained by the colchicine treatment on a diploid rose, 88/124-46 (Von Malek & Debener, 1998). Pollination experiments were conducted as described in **Table S6-1**. The results show that the diploid genotype (88/124-46) was SI, and the tetraploid genotype (CT40) was self-compatible.

**Table S6-1**. Pollination experiments on a diploid rose and its colchicine-induced tetraploid

| Genotype | Treatment | Result |
| --- | --- | --- |
| 88/124-46 | 14 hand-pollinated selfed flowers | 14 without seed-set |
| 88/124-46 | 10 hand-pollinated selfed flowers under heat stress of 35-40^o^C | 10 without seed-set |
| 88/124-46 | 10 flowers pollinated with pollen from 93/1-119 | 8 with seed-set |
| 88/124-46 | 14 flowers pollinated with pollen from 93/1-117 | 9 with seed set |
| CT40 | 10 flowers from 6 different plants hand-pollinated selfed | 10 flowers with seed-set |
| CT40 | 51 flowers self-pollinated without manual pollination | 51 flowers with seed-set |

All flowers were bagged before opening and in addition kept in insect proof greenhouse to exclude contamination. Greenhouse conditions: semi controlled with airation set to 20℃. Plants were kept in 3L pots.

**References**

Von Malek, B., & Debener, T. Genetic analysis of resistance to blackspot (*Diplocarpon rosae*) in tetraploid roses. *Theor. Appl. Genet.* **96**, 228-231 (1998).
