# Supplementary material for: The identification of the Rosa S-locus provides new insights into the breeding and wild origins of continuous-flowering roses
Source: Hortic Res. 2022 Oct 1;9:uhac155. doi: 10.1093/hr/uhac155 (PMC9527601; doi:10.1093/hr/uhac155)
Supplement: Web_Material_uhac155 [file web_material_uhac155.zip › Supplementary Information 7.docx]

**Supplementary information 7**

**The identification of the *Rosa* *S*-locus provides new insights into the breeding and wild origins of continuous-flowering roses**

Koji Kawamura^1*^, Yoshihiro Ueda^2,3^, Shogo Matsumoto^4^, Takanori Horibe^4,5^, Shungo Otagaki^4^, Li Wang^6^, Guoliang Wang^7,8^, Laurence Hibrad-Saint Oyant^9^, Fabrice Foucher^9^, Marcus Linde^10^, Thomas Debener^10^

^1^, Department of Environmental Engineering, Osaka Institute of Technology, Japan

^2^, Gifu International Academy of Horticulture, Japan

^3^, Gifu World Rose Garden, Japan

^4^, Graduate School of Bioagricultural Sciences, Nagoya University, Japan

^5^, College of Bioscience and Biotechnology, Chubu University, Japan

^6^, College of Life Sciences, Sichuan University, China

^7^, Jiangsu Provincial Department of Agriculture and Rural Affairs, China

^8^, Agricultural University of Nanjing, China.

^9^, Univ Angers, INRAE, Institut Agro, IRHS, SFR QUASAV, F-49000 Angers, France

^10^, Leibniz Universität, Hannover, Germany

^*^Corresponding author: Koji Kawamura

E-mail: [koji.kawamura@oit.ac.jp](mailto:koji.kawamura@oit.ac.jp)

Tel: +81-(0)6-4300-6848

Affiliation: Department of Environmental Engineering, Osaka Institute of Technology

Address: 5-16-1 Ohmiya, Asahi-ku, Osaka, 535-8585 JAPAN

***S*-locus *F*-box genes and their evolutionary divergence analyses**

*F-box genes surrounding the S-RNase genes were extracted from the genome database of Old Blush, R. multiflora, and R.rugosa. By using the F-box genes, homologous genes were also identified from RNA-seq data prepared from anthers of eight individuals of R. multiflora and one individual of R. rugosa. To assess the sequence divergence of the F-box genes compared to the S-RNase, the numbers of synonymous (Ks) and nonsynonymous (Ka) substitutions* *per site were estimated.*

**Materials & Methods**

ORFs longer than 1kbp were extracted from the 1Mbp genomic regions surrounding the *S-RNase* in the Old Blush, *R. multiflora*, and *R.rugosa* genome databases, and the *S*-locus *F*-box genes (*SLF*s) were identified by a Blast search of the ORFs. The *SLF* classified as type *k*, linked to the *S_x_* *S-RNase*, was termed *S_x_*-*SLF_k_*, according to the nomenclature of Kubo *et al*. (2015). RNA-seq data prepared from anthers and pistils of Old Blush, *R. multiflora*, and *R.rugosa* (**Table D3**) were used to calculate FPKM of the *SLF*s. By using the *SLFs* identified in the genome databases as queries, assembled RNA-seq data obtained from the anthers of eight individuals of *R. multiflora* and one individual of *R. rugosa* were blasted, and homologous *F-box* genes were also identified.

To assess the sequence divergence of *SLF*s, the numbers of synonymous (*Ks*) and nonsynonymous (*Ka*) substitutions per site were estimated using the Nei-Gojobori model (Nei & Gojobori, 1986) and the software MEGA 7 (Kumar *et al*., 2016). The analyses were conducted for *SLF* genes in the *S_C1_* haplotype (*S_1_*-*SLF*s) and *S_C2_* haplotype (*S_2_*-*SLF*s) of Old Blush genome, in the *S_15_* haplotype (*S_15_*-*SLF*s) and *S_16_* haplotype (*S_16_*-*SLF*s) of *R. multiflora* genome, and in the *S_18_* haplotype (*S_18_*-*SLF*s) and *S_20_* haplotype (*S_20_*-*SLF*s) of *R. rugosa* genome to estimate the intra-haplotypic divergences of *SLF*s. The inter-allelic divergence of each *SLF*s was also assessed by calculating the *Ks* and *Ka* values from *S_C1_-, S_C2_-, S_15_-, S_16_-, S_18_-,* and *S_20_*-*SLF_k_* (*k* indicates types of *SLF*, *k* = 1, 2, …11). The results were compared to the *Ks* and *Ka* values from the six *S-RNase* (*S_C1_, S_C2_, S_15_, S_16_, S_18_, S_20_*) and all 16 *S-RNase*, of which full lengths of cDNA sequences were determined in this study (**Table S3-1**).

**Results**

In the genome databases, 10-15 *F-box* genes were identified in 500kbp regions surrounding the *S-RNase*. All *F-box* genes were expressed in the stamen but not in the pistil (**Table** **S7-1**). Eleven SLFs were identified (**Fig.S7-1**). The SLF2s were separated in two clades but were defined as the same gene due to the homologous positions of the genes between the four haplotypic genomes (**Table S7-1a,b**). The evolutionary sequence divergence analysis of SLFs is well in agreement with the prediction from the collaborative non-self recognition model of SLF (Kubo *et al*., 2010). The *K_s_* and *K_a_* substitution rates of intra-haplotypic *SLFs* for each of the *S_C1_* to *S_20_* haplotypes (average *K_s_* = 0.83-0.89, *K_a_* = 0.43-0.46) are comparable to those of the *S_C1_* to *S_20_* *S-RNase*s (*K_s_* = 0.55, *K_a_* = 0.33; Fig.**S7-2**). This indicates that the sequences are highly diverged between SLFs in each haplotype, and the degrees of divergences are comparable to that of S-RNase. In contrast, the inter-allelic *K_s_* and *K_a_* values estimated for each *SLF* type are very low (average *K_s_* = 0.01-0.3; *K_a_* = 0.01-0.1). This indicates that the sequences are well conserved in each SLF between the six haplotypes.

**Table S7-1a**. List of candidate *S*-locus *F-box* genes identified by in silico blast search on *R. multiflora* genome database.

|  |  |  | Genomic position^2^ | | |  |  | FPKM^4^ | |
| --- | --- | --- | --- | --- | --- | --- | --- | --- | --- |
| Type^1^ | Gene name | S-genotype | Chromosome / Contig | Start | End | Length | Annotation^3^ | Stamen | Pistil |
| SLF7 | S_15__SLF7 | *S_15_* | Rmu_sc0001861.1 | 47382 | 48596 | 1215 | Rmu_sc0001861.1_g000008 | 6 | 0 |
| SLF4 | S_15__SLF4 | *S_15_* | Rmu_sc0001861.1 | 142485 | 141247 | 1239 | Rmu_sc0001861.1_g000044 | 1 | 0 |
| FBX6 | S_15__FBX6 | *S_15_* | Rmu_sc0001861.1 | 227599 | 226346 | 1254 | Rmu_sc0001861.1_g000063 | 19 | 1 |
| SLF2 | S_15__SLF2 | *S_15_* | Rmu_sc0001861.1 | 253944 | 255152 | 1209 | Rmu_sc0001861.1_g000073 | 31 | 1 |
| **S-RNase** | **S_15__SRNase** | ***S_15_*** | **Rmu_sc0001861.1** | **334742** | **302637** | **32106** | **NA** | **0** | **42** |
| SLF3 | S_15__SLF3 | *S_15_* | Rmu_sc0001861.1 | 383393 | 384625 | 1233 | Rmu_sc0001861.1_g000106 | 9 | 0 |
| FBX12 | S_15__FBX12 | *S_15_* | Rmu_sc00000046.1 | 9693 | 8374 | 1320 | Rmu_ssc0000046.1_g000006 | 4 | 0 |
| SLF1 | S_15__SLF1 | *S_15_* | Rmu_ssc0000046.1 | 60511 | 59264 | 1248 | Rmu_ssc0000046.1_g000013 | 10 | 1 |
| SLF11 | S_15__SLF11 | *S_15_* | Rmu_ssc0002607.1 | 161600 | 162820 | 1221 | Rmu_ssc0002607.1_g000025 | 3 | 0 |
| SLF10 | S_15__SLF10 | *S_15_* | Rmu_ssc0002607.1 | 189005 | 190276 | 1272 | Rmu_ssc0002607.1_g000032 | 7 | 0 |
| SLF9 | S_15__SLF9 | *S_15_* | Rmu_ssc0002607.1 | 200106 | 201332 | 1227 | Rmu_ssc0002607.1_g000034 | 15 | 0 |
| SLF8 | S_15__SLF8 | *S_15_* | Rmu_ssc0002607.1 | 204127 | 202871 | 1257 | Rmu_ssc0002607.1_g000035 | 3 | 0 |
| SLF6 | S_15__SLF6 | *S_15_* | Rmu_ssc0002607.1 | 220671 | 219403 | 1269 | Rmu_ssc0002607.1_g000042 | 10 | 1 |
| SLF5 | S_15__SLF5 | *S_15_* | Rmu_ssc0002607.1 | 248625 | 249896 | 1272 | Rmu_ssc0002607.1_g000051 | 9 | 1 |
| FBX7 | S_15__FBX7 | *S_15_* | Rmu_ssc0002607.1 | 274767 | 273526 | 1242 | Rmu_ssc0002607.1_g000058 | 4 | 0 |
| SLF7 | S_16__SLF7 | *S_16_* | Rmu_sc0000279.1 | 68523 | 67294 | 1230 | Rmu_sc0000279.1_g000014 | ND | ND |
| SLF5 | S_16__SLF5 | *S_16_* | Rmu_sc0000279.1 | 174741 | 176000 | 1260 | Rmu_sc0000279.1_g000046 | ND | ND |
| SLF6 | S_16__SLF6 | *S_16_* | Rmu_sc0000279.1 | 221176 | 222426 | 1251 | Rmu_sc0000279.1_g000061 | ND | ND |
| SLF1 | S_16__SLF1 | *S_16_* | Rmu_sc0006888.1 | 16472 | 15225 | 1248 | Rmu_sc0006888.1_g000002 | ND | ND |
| FBX1 | S_16__FBX1 | *S_16_* | Rmu_sc0006888.1 | 63515 | 62160 | 1356 | Rmu_sc0006888.1_g000015m | ND | ND |
| SLF2 | S_16__SLF2 | *S_16_* | Rmu_sc0006888.1 | 124146 | 122932 | 1215 | Rmu_sc0006888.1_g000026m | ND | ND |
| **S-RNase** | **S_16__SRNase** | ***S_16_*** | **Rmu_sc0006888.1** | **163515** | **146510** | **17006** | **NA** | **ND** | **ND** |
| SLF3 | S_16__SLF3 | *S_16_* | Rmu_sc0006888.1 | 210234 | 211466 | 1233 | NA | ND | ND |
| SLF4 | S_16__SLF4 | *S_16_* | Rmu_sc0006888.1 | 310084 | 311322 | 1239 | Rmu_sc0006888.1_g000084 | ND | ND |

^1^, Type was defined as either SLF (*S*-locus linked F-box) or FBX, with numbers indicating groups with similar protein sequences. We termed SLF when at least three *S*-alleles out of four (*S_C1_, S_C2_, S_15_, S_16_*) had the *F-box* genes, whereas we termed FBX only when one or two *S*-alleles had the *F-box* genes.

^2^, Genome data source is Nakamura *et al*. (2018) for *S_15_* and *S_16_*.

^3^, NA = No Annotation in original database. The sufflex ‘m’ indicates ‘modified’ annotation of original one.

^4^, FPKM (Fragments per kilobase of exon per million reads mapped) was calculated from the RNA-seq data of of two individuals of *R. multiflora* (Rm2, Rm3 for *S_15_*). Average FPKM of two individuals is shown for *R. multiflora* *S_15_*. ND = No Data.

**Table S7-1b**. List of candidate *S*-locus *F-box* genes identified by in silico blast search on *R. rugosa* genome databases.

|  |  |  | Genomic position^2^ | | |  |  | FPKM^4^ | |
| --- | --- | --- | --- | --- | --- | --- | --- | --- | --- |
| Type^1^ | Gene name | S-genotype | Chromosome | Start | End | Length | Annotation^3^ | Stamen | Pistil |
| FBX10 | S_18__FBX10 | *S_18_* | Chr4 | 5110820 | 5112046 | 1227 | Chr4.694 | 5 | 0 |
| FBX11 | S_18__FBX11 | *S_18_* | Chr4 | 5120542 | 5121786 | 1245 | Chr4.697 | 13 | 0 |
| FBX9 | S_18__FBX9 | *S_18_* | Chr4 | 5129847 | 5128585 | 1263 | Chr4.700 | 11 | 0 |
| FBX12 | S_18__FBX12 | *S_18_* | Chr4 | 5133695 | 5132364 | 1332 | Chr4.701 | 3 | 0 |
| FBX5 | S_18__FBX5 | *S_18_* | Chr4 | 5150734 | 5152053 | 1320 | Chr4.705 | 7 | 0 |
| SLF1 | S_18__SLF1 | *S_18_* | Chr4 | 5202666 | 5201407 | 1260 | Chr4.710 | 10 | 0 |
| FBX13 | S_18__FBX13 | *S_18_* | Chr4 | 5252414 | 5253670 | 1257 | Chr4.712 | 34 | 0 |
| FBX14 | S_18__FBX14 | *S_18_* | Chr4 | 5307122 | 5305889 | 1234 | Chr4.716 | 5 | 0 |
| **S-RNase** | **S_18__SRNase** | ***S_18_*** | **Chr4** | **5312675** | **5311423** | **1253** | **Chr4.718** | **0** | **372** |
| SLF3 | S_18__SLF3 | *S_18_* | Chr4 | 5328503 | 5329738 | 1236 | Chr4.719 | 17 | 0 |
| SLF4 | S_18__SLF4 | *S_18_* | Chr4 | 5494895 | 5496133 | 1239 | Chr4.727 | 13 | 0 |
| FBX8 | S_18__FBX8 | *S_18_* | Chr4 | 5565672 | 5564431 | 1242 | Chr4.733 | 62 | 0 |
| SLF7 | S_18__SLF7 | *S_18_* | Chr4 | 5611906 | 5610677 | 1230 | Chr4.736 | 17 | 0 |
| SLF5 | S_18__SLF5 | *S_18_* | Chr4 | 5618483 | 5617224 | 1260 | Chr4.738 | 8 | 0 |
| SLF6 | S_18__SLF6 | *S_18_* | Chr4 | 5625337 | 5626593 | 1257 | Chr4.740 | 8 | 0 |
| SLF8 | S_18__SLF8 | *S_18_* | Chr4 | 5657734 | 5658990 | 1257 | Chr4.742 | 8 | 0 |
| SLF9 | S_18__SLF9 | *S_18_* | Chr4 | 5663086 | 5661860 | 1227 | Chr4.743 | 8 | 0 |
| SLF10 | S_18__SLF10 | *S_18_* | Chr4 | 5669856 | 5668585 | 1272 | Chr4.744 | 1 | 0 |
| SLF11 | S_18__SLF11 | *S_18_* | Chr4 | 5709110 | 5707890 | 1221 | Chr4.748 | 5 | 0 |
| FBX4 | S_18__FBX4 | *S_18_* | Chr4 | 5712088 | 5710850 | 1239 | Chr4.749 | 17 | 0 |
| FBX4 | S_20__FBX4 | *S_20_* | Chr3 | 31895421 | 31896659 | 1239 | NA | ND | ND |
| FBX15 | S_20__FBX15 | *S_20_* | Chr3 | 31902999 | 31904207 | 1209 | NA | ND | ND |
| SLF10 | S_20__SLF10 | *S_20_* | Chr3 | 31944289 | 31945560 | 1272 | NA | ND | ND |
| SLF9 | S_20__SLF9 | *S_20_* | Chr3 | 31948931 | 31950145 | 1215 | NA | ND | ND |
| SLF8 | S_20__SLF8 | *S_20_* | Chr3 | 31954907 | 31953651 | 1257 | NA | ND | ND |
| SLF6 | S_20__SLF6 | *S_20_* | Chr3 | 31973998 | 31972943 | 1056 | NA | ND | ND |
| SLF5 | S_20__SLF5 | *S_20_* | Chr3 | 31983520 | 31984779 | 1260 | NA | ND | ND |
| SLF7 | S_20__SLF7 | *S_20_* | Chr3 | 32015831 | 32017060 | 1230 | NA | ND | ND |
| FBX8 | S_20__FBX8 | *S_20_* | Chr3 | 32026374 | 32027609 | 1236 | NA | ND | ND |
| SLF4 | S_20__SLF4 | *S_20_* | Chr3 | 32146540 | 32147778 | 1239 | NA | ND | ND |
| **S-RNase** | **S_20__SRNase** | ***S_20_*** | **Chr3** | **32191933** | **32176791** | **15143** | **NA** | **ND** | **ND** |
| SLF3 | S_20__SLF3 | *S_20_* | Chr3 | 32233973 | 32235205 | 1233 | NA | ND | ND |
| FBX14 | S_20__FBX14 | *S_20_* | Chr3 | 32275645 | 32276889 | 1245 | NA | ND | ND |
| SLF1 | S_20__SLF1 | *S_20_* | Chr3 | 32279789 | 32281039 | 1251 | NA | ND | ND |
| FBX5 | S_20__FBX5 | *S_20_* | Chr3 | 32313608 | 32312289 | 1320 | NA | ND | ND |
| FBX12 | S_20__FBX12 | *S_20_* | Chr3 | 32326033 | 32327367 | 1335 | NA | ND | ND |
| FBX9 | S_20__FBX9 | *S_20_* | Chr3 | 32329875 | 32331137 | 1263 | NA | ND | ND |
| FBX11 | S_20__FBX11 | *S_20_* | Chr3 | 32339137 | 32337851 | 1287 | NA | ND | ND |
| FBX10 | S_20__FBX10 | *S_20_* | Chr3 | 32344586 | 32343360 | 1227 | NA | ND | ND |

^1^, Type was defined as either SLF (*S*-locus linked F-box) or FBX, with numbers indicating groups with similar protein sequences. We termed SLF when at least three *S*-alleles out of four (*S_C1_, S_C2_, S_15_, S_16_*) had the *F-box* genes, whereas we termed FBX only when one or two *S*-alleles had the *F-box* genes.

^2^, Genome data source is Chen *et al*. (2021) for *S_18_*, Zang *et al*. (2021) for *S_20_*. Note that Chr4 of Chen *et al*. (2021) is homologous to the Chromosome 3 in the Old Blush genome.

^3^, NA = No Annotation in original database.

^4^, FPKM (Fragments per kilobase of exon per million reads mapped) was calculated from the RNA-seq data of one individuals of *R. rugosa* (Rg46 for *S_18_*). ND = No Data.

**Table S7-2.** Number of *S*-locus *F-box* genes identified from the genome databases and RNA-seq data of stamen of eight *R. multiflora* and one *R. rugosa*. ● = 1 allele, ●● = 2 alleles, ●●● = 3 alleles identified; × = Pseudogene; ○ = Partial sequence identified.

|  | Genome databases^1^ | | | | |  | RNA-seq of stamen (**Table D3**) | | | | | | | | |
| --- | --- | --- | --- | --- | --- | --- | --- | --- | --- | --- | --- | --- | --- | --- | --- |
|  | *R. chinensis* | | *R. multiflora* | *R. rugosa* | |  | *R. multiflora* | | | | | | | | *R. rugosa* |
|  |  |  |  |  |  |  | Rm1 | Rm2 | Rm3 | Rm08 | Rm09 | Rm27 | Rm28 | Rm33 | Rg46 |
|  | *S_C1_* | *S_C2_* | *S_15_/S_16_* | *S_18_* | *S_20_* |  | *S_C1_/S_14_* | *S_C1_/S_15_* | *S_C1_/_S15_* | *S_6_/S_7_* | *S_7_/S_8_* | *S_9_/S_11_* | *S_10_/S_13_* | *S_9_/S_12_* | *S_18_/S_19_* |
| *SLF1* | ● | ● | ●● | ● | ● |  | ●● | ●●● | ●● | ●● | ●● | ●○ | ●● | ●● | ●● |
| *SLF2* | ● | ● | ●● |  |  |  | ●○ | ●● | ●● | ●● | ●● | ●● | ●● | ●○ | ● |
| *SLF3* | ● | ● | ●● | ● | ● |  | ●● | ●● | ●● | ● | ●● | ●● | ●● | ●● | ● |
| *SLF4* | ● | ●● | ●● | ● | ● |  | ●● | ●● | ●● | ●●● | ●●● | ●●● | ●● | ●●● | ●●● |
| *SLF5* | ● | ● | ●● | ● | ● |  | ●● | ●● | ●● | ○○ | ●● | ●○ | ●● | ●● | ●● |
| *SLF6* | ● | × | ●● | ● | ● |  | ●●● | ●● | ●● | ●● | ●●● | ● | ●● | ●● | ● |
| *SLF7* |  | ● | ●● | ● | ● |  | ● | ● | ● | ●● | ●● | ●● | ●● | ●● | ●● |
| *SLF8* | ● | ● | ● | ● | ● |  | ●● | ●● | ●● | ●● | ●● | ●○ | ●● | ●● | ●● |
| *SLF9* | ● | ● | ● | ● | ● |  | ●● | ●● | ●● | ●● | ●● | ●● | ● | ● | ●● |
| *SLF10* | ● | ● | ● | ● | ● |  | ●● | ●● | ●● | ● | ●● | ●● | ● | ● | ●● |
| *SLF11* | ● | ● | ● | ● |  |  | ● | ●● | ●● | ●● | ● | ●● | ●● | ●● | ● |
| *FBX1* | ● |  | ● |  |  |  | ● | ● | ● |  |  |  |  |  |  |
| *FBX2* |  | ● |  |  |  |  |  |  |  | ● | ● |  | ● |  |  |
| *FBX3* |  | ● |  |  |  |  |  |  |  | ● |  |  |  |  |  |
| *FBX4* |  | ● |  | ● | ● |  |  |  |  |  |  |  |  |  | ●● |
| *FBX5* |  | ● |  | ● | ● |  |  |  |  |  |  |  |  |  | ●● |
| *FBX6* |  |  | ● |  |  |  |  | ● | ● |  |  |  | ● |  |  |
| *FBX7* |  |  | ● |  |  |  |  | ● | ● |  |  | ●●● | ● | ●○ |  |
| *FBX8* |  |  |  | ● | ● |  |  |  |  |  | ●● |  |  |  | ●● |
| *FBX9* |  |  |  | ● | ● |  |  |  |  |  |  |  |  |  | ●● |
| *FBX10* |  |  |  | ● | ● |  |  |  |  |  |  |  |  |  | ●● |
| *FBX11* |  |  |  | ● | ● |  |  |  |  |  |  |  |  |  | ●● |
| *FBX12* | × | × | ● | ● | ● |  |  |  |  |  | ● | ● | ●● |  | ● |
| *FBX13* |  |  |  | ● |  |  |  |  |  |  |  |  |  |  | ● |
| *FBX14* |  |  |  | ● | ● |  | ● |  |  |  |  |  |  |  | ● |
| *FBX15* |  |  |  |  | ● |  |  |  |  |  |  |  |  |  |  |

^1^, Genome data source is Raymond *et al.* (2018) for S_C1_, Hibrand Saint-Oyant *et al.* (2018) for S_C2_, Nakamura *et al.* (2018) for S_15_/S_16_, Chen *et al.* (2021) for S_18_, and Zang *et al.* (2021) for S_20_.

**
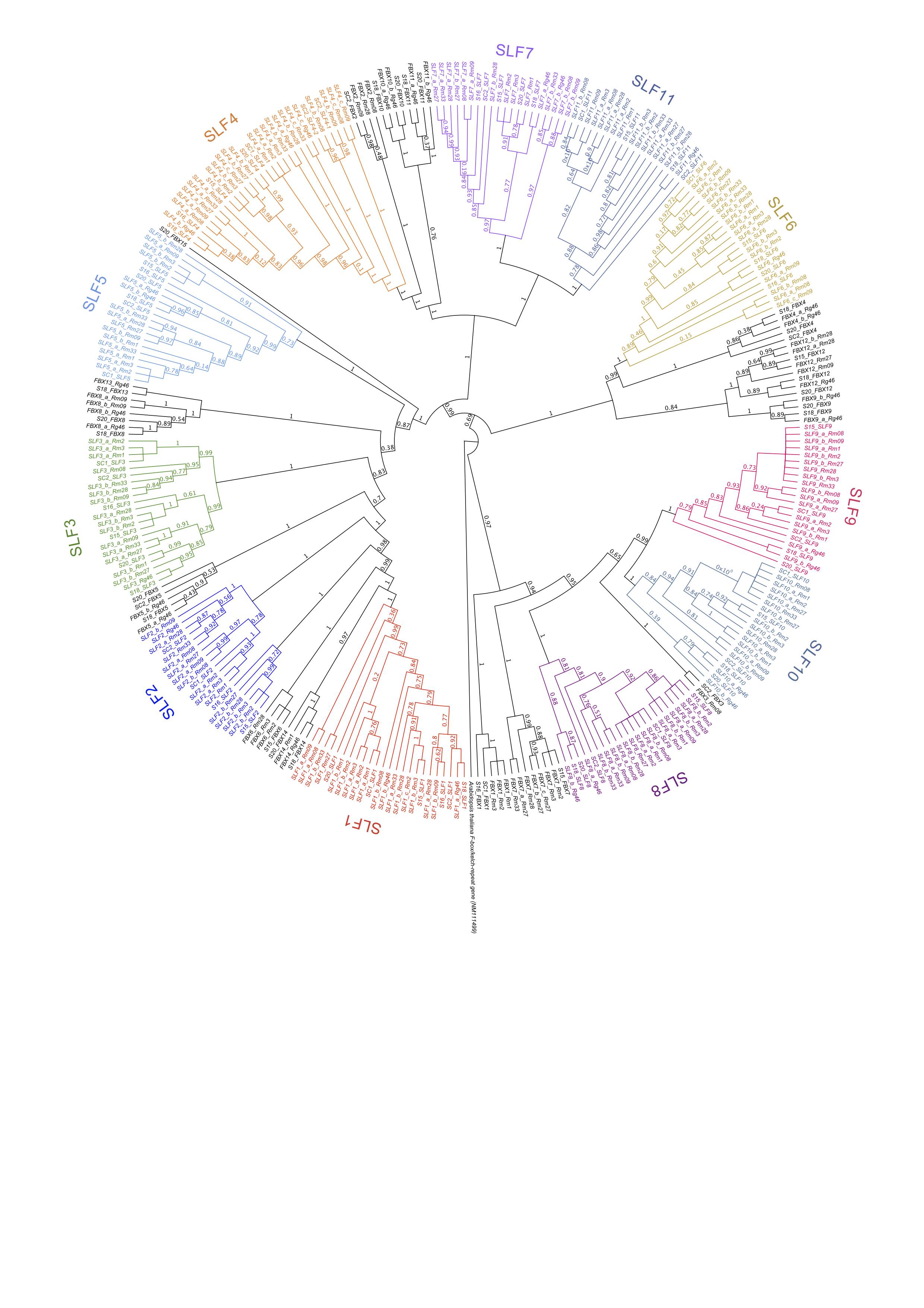
**

**Figure S7-1**. The molecular phylogenetic tree of *S*-locus *F-box* proteins in the rose. Deduced amino acid sequences were aligned using MUSCLE and then converted back to DNA sequences. The maximum-likelihood phylogenetic tree was constructed from the nucleotide protein-coding sequence alignment by FastTree (Price *et al*. 2009, 2010) using the Jukes-Cantor model of nucleotide evolution.The tree was rooted with *Arabidopsis thaliana* *F-box*/kelch-repeat gene (NM111499). Numbers below the branches represent FastTree support values based on 1,000 resamples.The *S*-locus *F*-box genes were identified from the genome databases of Old Blush, *R. multiflora*, and *R. rugosa* (**Table S7-1**) and their homologous genes identified from RNA-seq of stamen of eight *R. multiflora* and one *R. rugosa* (**Table S7-2**) are included in the tree. Newly-identified genes from the RNA-seq were named with the following rules: SLFx-like gene identified from RNA-seq of stamen of plant Y is named as SLFx_Y. If there are different sequences in one gene of a plant, we add a, b, or c before the plant ID, e.g., SLFx_a_Y, SLFx_b_Y. The cDNA sequences are available from Supplementary data **Table D5**. The RNA-seq data used for the analyses are described in Supplementary data **Table D3.**


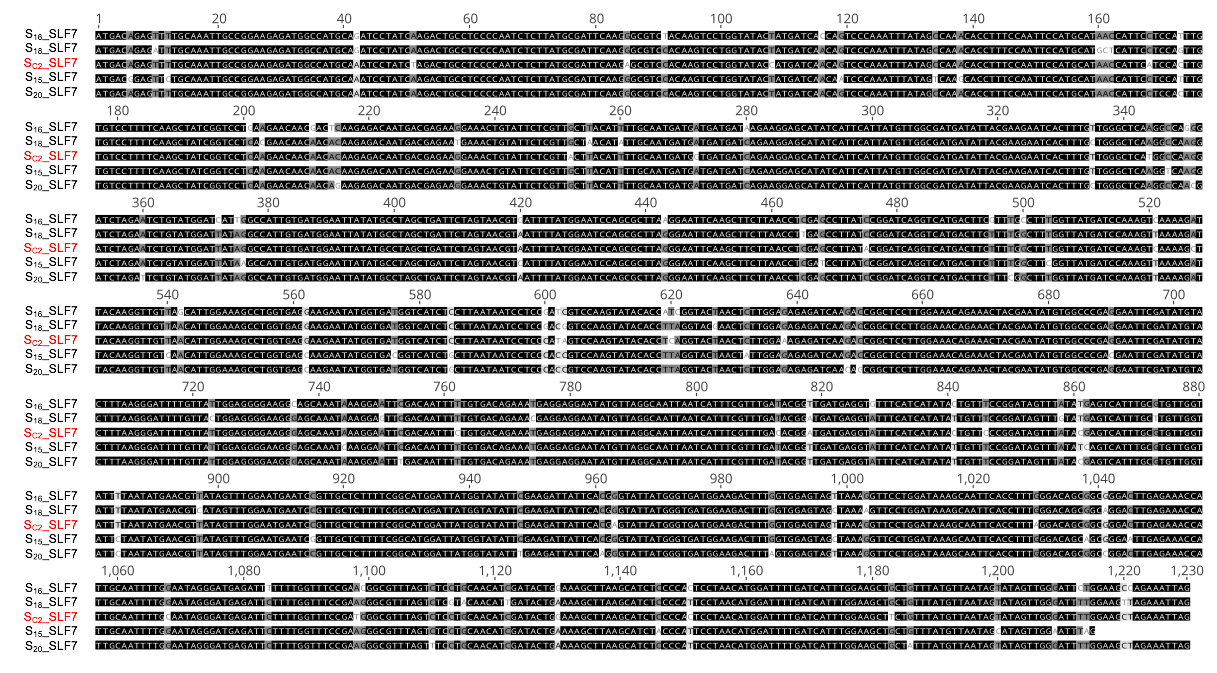


**Figure S7-2**. An alignment of cDNA sequence of *SLF7*. *S_C2__SLF7* is colocated with *S_C2_ S-RNase* on RC0 (chromosome 0) of Old Blush genome database of Hibrand-Saint Oyant *et al* (2018). Homologous genes were identified in the putative *S*-locus regions of *R. multiflora* (*S_15__SLF7*, *S_16__SLF7*; **Table S7-1a**) and *R. rugosa* genomes (*S_18__SLF7*, *S_20__SLF7*; **Table S7-1b**) and showed high sequence similarities with *S_C2__SLF7.*


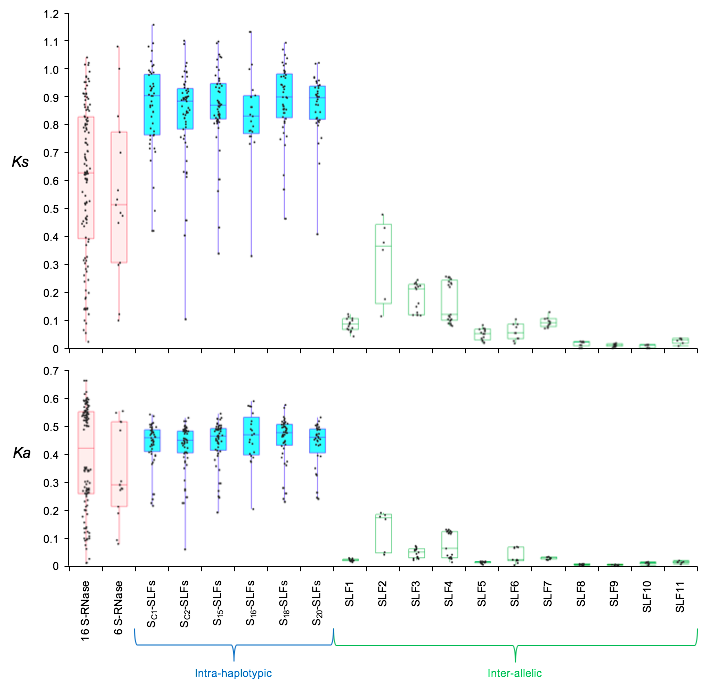


**Figure S7-3**. The numbers of synonymous (*K_s_*) and nonsynonymous (*K_a_*) substitutions per site estimated for *SLF* and *S-RNase*. The analyses were conducted for *SLF* genes in the *S_C1_* haplotype (*S_1_*-*SLF*s), *S_C2_* haplotype (*S_2_*-*SLF*s), *S_15_* haplotype (*S_15_*-*SLF*s), *S_16_* haplotype (*S_16_*-*SLF*s), *S_18_* haplotype (*S_18_*-*SLF*s), and *S_20_* haplotype (*S_20_*-*SLF*s) to estimate intra-haplotypic divergences of *SLF*s. The *Ks* and *Ka* values from the the six S-RNase (*S_C1_*, *S_C2_*, *S_15_*, *S_16_*, *S_18_*, *S_20_*) and those from the 16 S-RNases of which full-length cDNA sequences were determined (**Table S3-1**) are shown for comparison. The *SLF*_k_ (k = 1, 2, ..., 11) shows *K_s_* and *K_a_* values from *S_C1_*-, *S_C2_*-, *S_15_*-, *S_16_*-, *S_18_*-, and *S_20_*-*SLF_k_*, indicating the inter-allelic divergences of *SLF*. Boxes indicate the 25^th^ and 75^th^ percentiles of data, and bars indicate the 10^th^ and 90^th^ percentiles of data. Black dots show individual data points.

**References**

Chen, F. *et al.* A chromosome-level genome assembly of rugged rose (*Rosa rugosa*) provides insights into its evolution, ecology, and floral characteristics. *Hortic Res* **8,**141 (2021).

Hibrand Saint-Oyant L *et al*. A high-quality genome sequence of Rosa chinensis to elucidate ornamental traits. *Nature Plants* **4**: 473-484 (2018).

Kubo, K. *et al.* Gene duplication and genetic exchange drive the evolution of S-RNase-based self-incompatibility in *Petunia*. *Nat. Plants* **8**: 14005 (2015).

Price, MN. *et al.* FastTree: computing large minimum evolution trees with profiles instead of distance matrix. *Mol Biol Evol* **26**: 1641-50 (2009).

Prince, MN. *et al.* FastTree 2 – Approximately maximum-likelihood trees for large alignments. *Plos ONE* **5**: e9490 (2010).

Raymond O. *et al*. The Rosa genome provides new insights into the domestication of modern roses. *Nature Genet.* **50**: 772-777 (2018).

Zang, F. *et al*. A high-quality chromosome-level genome of wild *Rosa rugosa*, *DNA Res.* **28**: dsab017 (2021).
