# Supplementary material for: The identification of the Rosa S-locus provides new insights into the breeding and wild origins of continuous-flowering roses
Source: Hortic Res. 2022 Oct 1;9:uhac155. doi: 10.1093/hr/uhac155 (PMC9527601; doi:10.1093/hr/uhac155)
Supplement: Web_Material_uhac155 [file web_material_uhac155.zip › Supplementary Information 8.docx]

**Supplementary information 8**

**The identification of the *Rosa* *S*-locus provides new insights into the breeding and wild origins of continuous-flowering roses**

Koji Kawamura^1*^, Yoshihiro Ueda^2,3^, Shogo Matsumoto^4^, Takanori Horibe^4,5^, Shungo Otagaki^4^, Li Wang^6^, Guoliang Wang^7,8^, Laurence Hibrad-Saint Oyant^9^, Fabrice Foucher^9^, Marcus Linde^10^, Thomas Debener^10^

^1^, Department of Environmental Engineering, Osaka Institute of Technology, Japan

^2^, Gifu International Academy of Horticulture, Japan

^3^, Gifu World Rose Garden, Japan

^4^, Graduate School of Bioagricultural Sciences, Nagoya University, Japan

^5^, College of Bioscience and Biotechnology, Chubu University, Japan

^6^, College of Life Sciences, Sichuan University, China

^7^, Jiangsu Provincial Department of Agriculture and Rural Affairs, China

^8^, Agricultural University of Nanjing, China.

^9^, Univ Angers, INRAE, Institut Agro, IRHS, SFR QUASAV, F-49000 Angers, France

^10^, Leibniz Universität, Hannover, Germany

^*^Corresponding author: Koji Kawamura

E-mail: [koji.kawamura@oit.ac.jp](mailto:koji.kawamura@oit.ac.jp)

Tel: +81-(0)6-4300-6848

Affiliation: Department of Environmental Engineering, Osaka Institute of Technology

Address: 5-16-1 Ohmiya, Asahi-ku, Osaka, 535-8585 JAPAN

**Co-segregation of *SLF5* in RC3, and *S_C2_* *S-RNase* in RC0**

*Co-segregation of SLF5 in the S-locus region of chromosome 3 (RC3) and S_C2_ S-RNase in chromosome 0 (RC0), was demonstrated by using the 97 F_1_-hybrid population FW.*

**Materials & Methods**

Partial sequences of *SLF5* were obtained from the parent TF and RW, and the CAPS marker was designed to analyze the segregation of *SLF5*. PCR amplification of *SLF5* was performed using *SLF5* primers (**Table D1**) and EmeraldAmp PCR Master Mix (TaKaRa) with thermal cycling: (1) 2 min of 95°C; (2) 30 s of 95°C; (3) 30 s of 60°C; (4) 20 s of 72°C; and (5) go back to step (2) 39 times. The 5μL of the PCR product was digested by 0.5 U of *ScaI* (Promega).

**Figure S8-1.** Partial sequences of *SLF5* of OB, RW, and TF.

**Results**

The *SLF5* alleles (A/G) (**Fig. S8-2**) of TF were perfectly co-segregated with the *S-RNase* alleles (*S_C2_*/*S_21_*) in 97 *F_1_* hybrids, indicating that the *S_C2_* *S-RNase* in RC0 should be located in the *S*-locus region of RC3.


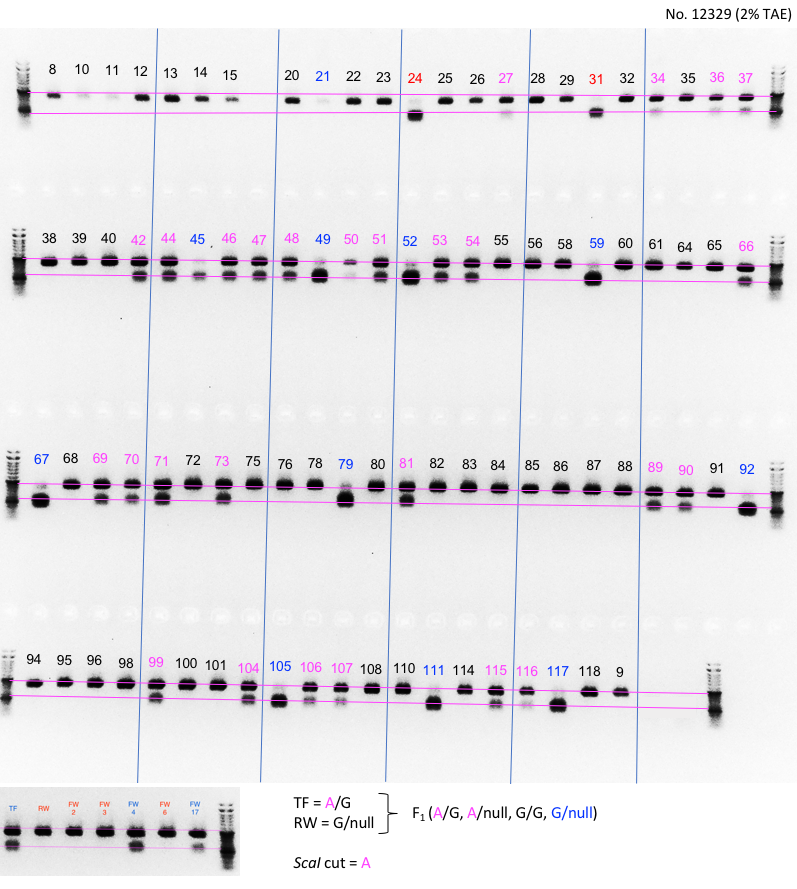


**Figure S8-2.** Agarose gel image for the result of the *ScaI*-CAPS marker of *SLF5* in the FW mapping population.
