# Supplementary material for: The identification of the Rosa S-locus provides new insights into the breeding and wild origins of continuous-flowering roses
Source: Hortic Res. 2022 Oct 1;9:uhac155. doi: 10.1093/hr/uhac155 (PMC9527601; doi:10.1093/hr/uhac155)
Supplement: Web_Material_uhac155 [file web_material_uhac155.zip › Supplementary Information 9.docx]

**Supplementary information 9**

**The identification of the *Rosa* *S*-locus provides new insights into the breeding and wild origins of continuous-flowering roses**

Koji Kawamura^1*^, Yoshihiro Ueda^2,3^, Shogo Matsumoto^4^, Takanori Horibe^4,5^, Shungo Otagaki^4^, Li Wang^6^, Guoliang Wang^7,8^, Laurence Hibrad-Saint Oyant^9^, Fabrice Foucher^9^, Marcus Linde^10^, Thomas Debener^10^

^1^, Department of Environmental Engineering, Osaka Institute of Technology, Japan

^2^, Gifu International Academy of Horticulture, Japan

^3^, Gifu World Rose Garden, Japan

^4^, Graduate School of Bioagricultural Sciences, Nagoya University, Japan

^5^, College of Bioscience and Biotechnology, Chubu University, Japan

^6^, College of Life Sciences, Sichuan University, China

^7^, Jiangsu Provincial Department of Agriculture and Rural Affairs, China

^8^, Agricultural University of Nanjing, China.

^9^, Univ Angers, INRAE, Institut Agro, IRHS, SFR QUASAV, F-49000 Angers, France

^10^, Leibniz Universität, Hannover, Germany

^*^Corresponding author: Koji Kawamura

E-mail: [koji.kawamura@oit.ac.jp](mailto:koji.kawamura@oit.ac.jp)

Tel: +81-(0)6-4300-6848

Affiliation: Department of Environmental Engineering, Osaka Institute of Technology

Address: 5-16-1 Ohmiya, Asahi-ku, Osaka, 535-8585 JAPAN

**Linkage between the *S*-locus and important ornamental traits**

*The degree of genetic linkage was tested between the S-locus and the genes underlying continuous-flowering (KSN) and double flower characteristics (AP2-like) in two F_1_ mapping populations. Details in methods and results of genotyping are described.*

**Materials & Methods**

Two *F_1_* diploid mapping populations were used. One is the FW mapping population (Kawamura *et al*., 2011) and the other is the 94/1 mapping population (Debener & Mattiesch, 1999). The CF characteristics is inherited as a recessive trait controlled by the non-functional allele *ksn* (Iwata *et al*., 2012), whereas the double-flower phenotype is inherited as a dominant trait controlled by the non-functional allele *ap2* (Hibrand Saint-Oyant *et al*., 2018)*.*

*Linkage between ksn^copia^ and the S-locus*

TF is CF with *ksn^copia^*/*ksn^copia^*, and RW is once-flowering (OF) with *KSN^w^*/*ksn^copia^*. The FW population segregates in flowering behavior, and there is no recombination between the phenotype (CF/OF) and the *KSN* genotype (Kawamura *et al*., 2011). We therefore analyzed the segregation patterns of the *KSN^w^*/*ksn^copia^* alleles of RW, and the *S_1w_*/*S_x_* alleles of the RW *S*-locus (Supplementary information 4) and calculated the recombination frequency. Since both the parents of the 94/1 population are CF (Debener & Mattiesch, 1999), we cannot estimate the recombination frequency between *S* and *KSN* in this population.

*Linkage between ap2 and the S-locus*

Both the FW and 94/1 populations segregate into single versus double flower phenotypes. The TF has double flower, and the RW has single flower. The parent of the 94/1 population, 93/1-119 has double flower, and 93/1-117 has single flower. The TF and 93/1-119 have the transposon-inserted allele *ap2*, as well as a wild functional allele of *AP2*, i.e., they have the *ap2*/*AP2* genotype. We therefore analyzed the segregation patterns of *ap2* from the TF and the 93/1-119 in their *F_1_* hybrids, together with the segregation patterns of the *S*-alleles of the TF (Supplementary information 4) and the 93/1-119 (Supplementary information 5), and calculated the recombination frequencies between *ap2* and the *S*-locus. Specific primers (ap2_F, ap2_R) were used to amplify the transposon-inserted allele. PCR amplification was performed using EmeraldAmp PCR Master Mix (TaKaRa) with thermal cycling: (1) 2 min of 95°C; (2) 30 s of 95°C; (3) 30 s of 60°C; (4) 20 s of 72°C; and (5) go back to step (2) 29 times. *See* **Table D1** for primer information.

We have also genotyped the segregation of the wild *AP2* alleles of the RW to calculate its recombination frequency with the *S*-locus. Partial sequences of two wild alleles of *AP2* in the RW, and one wild allele of the TF, were determined, and a CAPS marker with *RsaI* was designed to perform the genotyping of the wild alleles of RW and TF (**Fig.S9-1**).

**Figure S9-1**. Alignments of partial sequences of the wild alleles of the *AP2*-like gene in TF and RW. TF has one wild allele, and RW has two alleles.

**Results & Discussion**

The *KSN* gene has a 13.5Mbp distance from the *S*-locus, and the *AP2*-like gene has a 9.0 Mbp distance from the *S*-locus (**Table 4** in main manuscript). The recombinant frequency between *S* and *AP2*-like is 13% in RW, 40% in TF, and 22% in 93/1-119, and the frequency of *S* and *KSN* is 20% in RW.

In both populations, the co-segregation of phenotype (double flower/single flower) and genotype (the presence of *ap2*) is not perfect, with two recombinants per 97 hybrids (FW) and per 50 hybrids (94/1). This can result from the effects of another minor gene controlling the number of petals in the rose (Roman *et al*., 2015).

**Figure S9-2** shows the relationship between map cM and genomic distance (bp) in the chromosome 3 of the FW map. The recombination appears to be restricted in TF after *AP2* and in RW after *KSN*, although a dense map construction with markers scattered over the whole chromosome is needed to clarify the observed patterns. One possible explanation for this pattern is that the heterozygous alleles with the retrotransposon-inserted allele (i.e., *ap2*/*AP2* in TF, and *ksn^copia^*/*KSN^w^* in RW) restrict normal chromosome pairing and recombination.

**Figure S9-2**. Comparison of two parental maps, TF3 versus RW3. **(a)** Genetic linkage maps (chromosome 3) of TF and RW. **(b)** Plot of map positions versus genomic positions of markers used to construct the genetic map. From the *S*-locus to the *AP2* gene, map position (cM) steeply increases in TF. In contrast, map position gradually approaches a plateau after *KSN* in RW.

**References**

Debener, T., & Mattiesch, L. Construction of a genetic linkage map for roses using RAPD and AFLP markers. *Theor. Appl. Genet.* **99**, 891-899 (1999).

Hibrand Saint-Oyant L *et al*. A high-quality genome sequence of Rosa chinensis to elucidate ornamental traits. *Nature Plants* **4**: 473-484 (2018).

Iwata, H. *et al*. The TFL1 homologue KSN is a regulator of continuous flowering in rose and strawberry. *Plant J.* **69**, 116-25 (2012).

Kawamura, K. *et al.* Quantitative trait loci for flowering time and inflorescence architecture in rose. *Theor. Appl. Genet.* **122**, 661-75 (2011).

Roman, H. *et al.* Genetic analysis of the flowering date and number of petals in rose. *Tree Genet. Genomes* **11**, 85 (2015).
